# Supplementary material for: Longitudinal associations between marine omega-3 supplement users and coronary heart disease in a UK population-based cohort
Source: BMJ Open. 2017 Oct 13;7(10):e017471. doi: 10.1136/bmjopen-2017-017471 (PMC5652534; doi:10.1136/bmjopen-2017-017471)
Supplement: Supplementary file 1 [file bmjopen-2017-017471supp001.pdf]

Appendices

Appendix I: Methods - extended version ... 2
Appendix II: Consistency of and change in supplement use from data collected at three time points in EPIC-Norfolk (1993-2011). ... 13
Appendix III: Fish or Supplement? ... 14
Appendix IV: Multiple Imputation ... 20
Appendix V: Characteristics of participants by type of supplement used as measured at DSA1 (1993-1998). Values are median (interquartile range) for continuous variables and % for categorical variables. ... 24
Appendix VI: Hazard ratios for CHD mortality by supplement user subgroups assessed at DSA1 only. ... 26
Appendix VII: Interactions using time-varying covariate analysis for the association between supplement use and risk of CHD mortality (1993-2015). ... 27

Abbreviations:

DSA: Dietary Supplement Assessment

HE: Health Examination

HLQ: Health and Lifestyle Questionnaire

NSU: Non-Supplement User

SU: Supplement user

EPA: eicosapentaenoic acid

DHA: docosahexaenoic acids

n-3 PUFA: omega-3 poly unsaturated fatty acids (EPA+DHA)

7dDD: 7-day Diet Diary

Appendix I: Methods - extended version

This Appendix may be read as a replacement of the method section in the manuscript.

Study design

Recruitment for the Norfolk-based European Prospective Investigation into Cancer and Nutrition (EPIC-Norfolk) started in 1993 and was completed in 1998 [1,2]. Thirty-five general practices in the Norfolk area of East-Anglia (UK) took part in the study; 77,630 registered patients were approached, 30,445 consented. Exposure assessment has been repeated over the course of the study; participants completed general Health and Lifestyle Questionnaires (HLQ) and attended three completed rounds of health examinations (HE) up to 2011 (Figure 1, Appendix I-Table 1). Data on health outcomes and mortality were collected passively. Ethical approval for the study was given by the Norwich District Health Authority Ethics Committee. Participants gave written informed consent.

Appendix I-Table 1: Availability of variables from questionnaires and health examinations in the EPIC-Norfolk study (1993-2011) for all those who consented.

	HLQ (1993-1998)	HE1 (1993-1998) *DSA1*	HE2 (1998-2000)	FU3 (2002-2004) *DSA2*	FU4 (2004-2011) *DSA3*	HE3 (2004-2011)
Approached for questionnaire (n)	77,630			25,846	18,380	
Completed questionnaire (n)	30,411			17,585	10,874	
Invited for HE (n)		30,445	19,560			10,826
Attended HE (n)		25,639	15,786			8,623
Retention (% relative to attenders HE1)			62	69	42	34
Measured anthropometry obtained at HE						
Height-Weight-BMI (n)		25,582	15,758			8603
Self-reported socio-economic & lifestyle factors from questionnaires						
Social Class (n)	29,673					
Education level (n)	30,411					
Marital status (n)	30,247			17,447	10,770	
Self-reported illnesses (n)	30,411			17,585	***10,874	
Smoking (n)	30,158			17,494	10,870	
Physical activity (n)	30,410			17,585	10,873	
Alcohol consumption (n)	30,051			17,429	10,592	
Diet measured using a 7dDD* (n)		25,525				
*Self-reported supplement use from questionnaires***						
Completed (n)		23,039		17,574	10,870	
Type of instrument		7dDD		HLQ	HLQ	
Recall time		1 week		1 week	1 week	
Median time since previous DSA (IQR), years		-		7.6 (7.1, 8.1)	5.6 (4.0, 6.5)	

HE, health examination; FU, follow-up; DSA, dietary supplement assessment; 7dDD, 7-day Diet Diary; HLQ, Health and Lifestyle Questionnaire; IQR, Interquartile Range.

*7-day Diet Diaries were repeated at HE2 and HE3, but only a small, non-randomly selected proportion of these data have been entered and are available for statistical analysis. Equally, Food Frequency Questionnaires have been administered at all HE; however, only the data from the FFQ at HE1 and HE2 have been entered and are available for analysis; moreover, the supplement data from the FFQ have not been entered using the ViMiS system [3] and were hence not available or compatible with the supplement data used in the described analysis. For these reasons, only baseline dietary data, using the 7dDD, have been used in this analysis.

** Dietary supplement use was assessed at *five* time points (two additional HLQs were situated in between DSA1 and DSA2). These additional DSA were hybrid questions, combining supplement use with medication use, the answers to which did not follow cohort trends or indeed trends in national survey data. The first additional DSA had the extra complication that the supplement/medication question was only introduced after some time, making the data only available on a subset of the participants. For these reasons, only three dietary supplement assessments (referred to as 'DSA', and for ease, numbered consecutively) were useable for analysis all with a recall time of one week (see also Figures 1, 2 and 3 in this Appendix).

***Data are obtained from a combination of self-reported illnesses at the time of the HLQ and FU3 since this question was not asked for at FU4. Missing values were assumed to mean 'no'.

Assessment of n-3 PUFA supplement use

Dietary supplements were defined according to the EU directive 2002/46/EC [4]: "Food stuffs the purpose of which is to supplement the normal diet and which are concentrated sources of nutrients or other substances with a nutritional or physiological effect, alone or in combination, marketed in dose form… ." Prescribed medication containing minerals and/or vitamins (e.g. ferrous sulphate) were not considered supplements. The sum of EPA and DHA from supplements were the main exposure, referred to as n-3 PUFA. We used data from three dietary supplement assessments (DSA), named DSA1, DSA2, DSA3. The three DSA are from different postal data collection methods with a slightly different phrasing of the supplement use question, but all covered a one week recall and shared basic question elements such as naming of vitamins or food supplements and a request for the supplement composition (Figures 1-3 in this Appendix).

Appendix I-Figure 1: DSA1

1st version, used from 1994 through out 1996

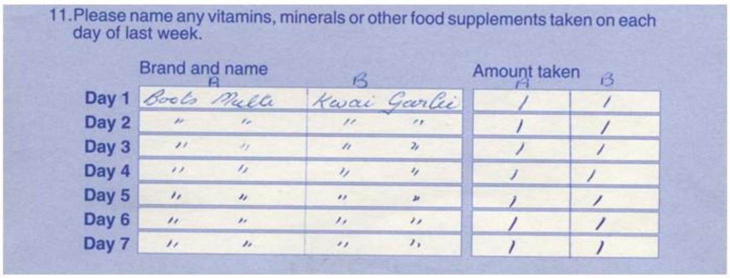

11. Please name any vitamins, minerals or other food supplements taken on each day of last week.

	Brand and name A	Brand and name B	Amount taken A	Amount taken B
Day 1	Boots Multi	Kwai Garlic	/	/
Day 2	" "	" "	/	/
Day 3	" "	" "	/	/
Day 4	" "	" "	/	/
Day 5	" "	" "	/	/
Day 6	" "	" "	/	/
Day 7	" "	" "	/	/

2nd version, used between 1996 and 1998.

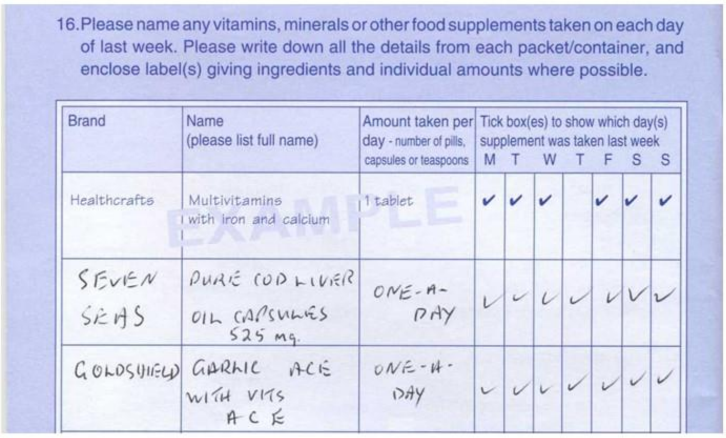

16. Please name any vitamins, minerals or other food supplements taken on each day of last week. Please write down all the details from each packet/container, and enclose label(s) giving ingredients and individual amounts where possible.

Brand	Name (please list full name)	Amount taken per day - number of pills, capsules or teaspoons	M	T	W	T	F	S	S
Healthcrafts	Multivitamins with iron and calcium	1 tablet	✔	✔	✔		✔	✔	✔
SEVEN SEAS	PURE COD LIVER OIL CAPSULES 525 mg.	ONE-A-DAY	✔	✔	✔	✔	✔	✔	✔
GOLDSHIELD	GARLIC ACE WITH VITS A C E	ONE-A-DAY	✔	✔	✔	✔	✔	✔	✔

(Column header for day ticks: Tick box(es) to show which day(s) supplement was taken last week. The Healthcrafts row is marked EXAMPLE.)

Appendix I-Figure 2: DSA2

MEDICATIONS AND SUPPLEMENTS *(continued)*

86 In **the last week**, have you taken any vitamins, food supplements etc. Yes ☐ No ☐ Don't know ☐

(Please name any vitamins, minerals or other food supplements taken on each day of last week. Please write down all the details from each packet/container.)

Brand	Name (Please list full name)	Amount taken per day – number of pills, capsules or teaspoons.	Tick (✓) box(es) to show which day(s) supplement was taken last week.						
			M	T	W	T	F	S	S

Appendix I-Figure 3: DSA3

63. In the last week, have you taken any vitamins, food supplements etc? Yes ☐ No ☐ Don't know ☐

(Please name any vitamins, minerals or other food supplements taken on each day of last week **as in the example below**.)

Brand	Name (Please list **full** name)	Amount taken per day – number of pills, capsules or teaspoons.	Tick (✓) box(es) to show which day(s) supplement was taken last week.						
			M	T	W	T	F	S	S
Healthcrafts	Multivitamins with iron and calcium	1 tablet	✓		✓		✓	✓	

At each DSA, participants were grouped by the type of supplement they consumed:

- non-supplement users (NSU);
- non n-3 PUFA supplement users (SU-n3): participants who consumed one or more supplements that did not contain n-3 PUFA;
- n-3 PUFA supplement users (SU+n3): participants who used n-3 PUFA supplements, either singly or in combination with other non n-3 PUFA supplements. The supplements included in the n-3 PUFA group contained cod liver oil (92%) and/or other types of fish oil (19%) such as halibut liver oil; 81% of the SU+n3 used a supplement which contained only cod liver oil.

Participants were also grouped into five categories identifying change in n-3 PUFA supplement use between two consecutive DSA (*i.e.* DSA1-DSA2; DSA2-DSA3):

Appendix I-Figure 4: Supplement change categories (see also Appendix II).

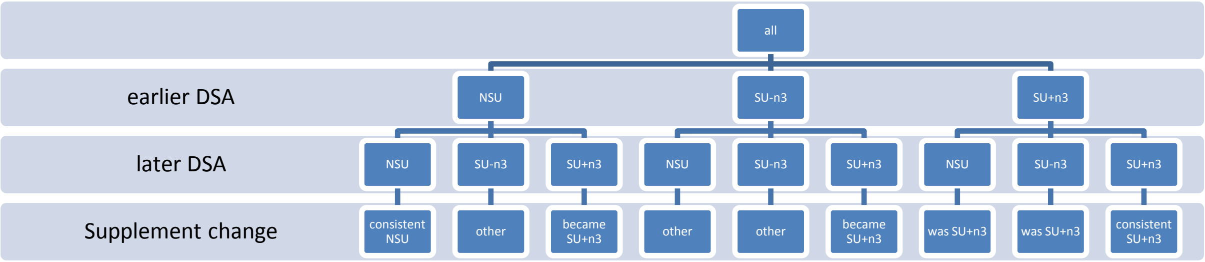

The Vitamin and Mineral Supplement (ViMiS) system to calculate the nutrient quantity from supplements assessed at DSA1, has been described in detail elsewhere [3]. In brief, the supplement described by the participant was coded with regards to supplement name, brand name, strength/unit, type and frequency of consumption. These data were matched against data collected from manufacturers' labels of 2162 dietary supplements. Where no match could be found, one of the 600 best-fitting generic supplement nutrient compositions was assigned. Supplement quantification of nutrients was made compatible with nutrient quantification in food composition tables. Missing values of two of the n-3 PUFAs, EPA and DHA, in supplements were replaced with cod liver oil/fish-specific, capsule/liquid-specific as well as strength-specific nutrient values. Average daily nutrient composition from

supplements was calculated and added to the average daily food intake to obtain average total nutrient intake. N-3 PUFA intake were compared with n-3 PUFA biomarkers (plasma EPA and DHA) available on a subset (n=6467) of EPIC-Norfolk participants; we observed a 28% higher concentration of plasma n-3 PUFA among SU+n3 *vs*. NSU (median [interquartile range, IQR] of plasma n-3 PUFA concentrations were: NSU 268 [206-354] umol/L, SU-n3 281 [223-382] umol/L, SU+n3 343 [256-453] umol/L) [5].

Dietary covariates

The analyses adjust for potential dietary confounders measured at DSA1. Diet was assessed using a 7-day diet diary (7dDD) between 1993-1998 [6]. Participants were asked to record food and drinks consumed in an A5-size booklet where the days were pre-structured into seven meal occasions and a checklist. Portion sizes could be described using colour photographs, household measures, or food packaging. A nurse completed the first day as a 24-hour diet recall in order to indicate the level of detail necessary. Data were entered by trained staff into DINER [7] and checked and calculated by nutritionists using DINERMO [8]. The underlying food composition tables were derived from McCance & Widdowson's 5th edition [9], including the supplementary data [10–19]. Missing values for fatty acids were further completed using a recipe calculation system [20,21]. The baseline 7dDD provided data on average daily energy intake (MJ/d), proportion of energy provided by macronutrients (%En), the sum of EPA and DHA intake (g/d) referred to as 'n-3 PUFA' and consumption of the disaggregated amounts of fruit, vegetable, red meat, processed meat, white meat, and white and oily fish consumption (g/d) [8].

Assessment of other covariates

The analyses at all DSA adjust for a number of non-dietary variables which are potential confounders. Social class was measured through occupational status, which was classified according to the registrar general's occupational classification scheme [22] (professional, managerial, skilled non-manual, skilled manual, semi-skilled, non-skilled). Highest education level achieved was divided in four categories (no qualification, O-level, A-level, degree). Both variables were derived from HLQ1 (coinciding with DSA1) and are time-fixed. The remaining covariates are time-dependent and refer to measures obtained at time points corresponding to the DSA. We identified participants at higher risk of CHD using responses

to the questions “Has the doctor ever told you that you have [myocardial infarction/diabetes/stroke]?”. Participants were classified as a never smoker, former smoker or current smoker based on responses to the questions “Have you ever smoked as much as one cigarette a day for as long as a year?” and “Do you smoke cigarettes now?”. Alcohol consumption, was classified as none, >0-14 units/wk, >14-28 units/wk, >28 units/wk using the sum of the number of units consumed per week of beer, spirits, wine and fortified wine. Marital status was re-categorised into married (married/living as married) or non-married (widowed, divorced, separated, single). Participants were classified as being active, moderately active, moderately inactive or inactive using a validated 4-point scale taking into account both occupational and leisure time activity [23]. At the HE, BMI (kg/m^2) was calculated from height and weight measured by a trained nurse. The collection of non-dietary variables did not always coincide exactly with the DSA: see Table 1 (in this Appendix) for an overview of available variables at respective time points.

Participant selection, case ascertainment and outcomes studied

Participants were eligible for analyses if they provided data on supplement use at any of the three time points (DSA1, DSA2, DSA3) and attended the corresponding HE (Figure 1). Four thousand and thirty participants were not included at any time point studied. The participant’s National Health Services number (a unique national patient identifier) was linked to the data from the Office of National Statistics to obtain vital status and causes of death. A similar procedure was followed for causes of hospital admissions registered by the East Norfolk Primary Health Care Trust which records for its residents the admissions in England and Wales. The main endpoint studied was Coronary Heart Disease (CHD) mortality mentioned anywhere on the death certificate, identified with ICD9 410-414 or ICD10 I20-I25. In sensitivity analyses, we investigated the use of different definitions for the CHD endpoint: (i) CHD mortality mentioned as underlying cause of death, (ii) death due to acute myocardial infarction (ICD-codes 410 or I21), (iii) first recorded hospitalisation due to CHD. We hypothesised to observe associations between n-3 PUFA containing supplements and CHD death; however, considering the different etiological pathways of atherosclerosis we did not expect to find an association at this low ingested dose of n-3 PUFA [5] and hospitalisation (non-fatal) CHD [24].

Statistical analysis

A description of the cohort at DSA1, DSA2 and DSA3 was made for supplement use, socio-demographic characteristics, self-reported illnesses, anthropometry, and nutrient and food intake. For categorical variables, counts and percentages were used; for continuous variables, median and IQR were used.

To study potential changes in supplement user characteristics over time, a multinomial logistic regression analysis was performed at each DSA with supplement use group as the outcome variables and the variables noted above as explanatory variables.

The main analyses are based on Cox proportional hazards models, with follow-up time as the underlying time variable. Individuals who died from causes other than the event of interest were censored at their date of death and individuals who did not die from any cause were censored at the end of follow-up (31st March 2015). Follow-up was therefore for up to 22 years (1993 to 2015).

The Cox regression modelling began by investigating associations between supplement use at DSA1 (SU+n3 *vs.* NSU and SU-n3 *vs.* NSU) and CHD mortality, with adjustment for covariates measured at DSA1. A series of *cumulative* adjustment models were used: sex and age-adjusted estimates (model 1); including smoking, BMI (kg/m^2), physical activity, alcohol intake, social class, education and season in which the questionnaire was completed (model 2); including self-reported myocardial infarction, stroke or diabetes (model 3); including energy intake (MJ/d) and disaggregated fruit (g/d), vegetable (g/d), red meat & processed meat (g/d), and white meat (g/d) (model 4); including white fish (g/d) and oily fish (g/d) (model 5). Models 1-5 were also fitted using quintiles of n-3 PUFA intake from food and supplements at DSA1 as the main exposure in place of supplement groupings. Note that Model 5 includes adjustment for fish consumption, which is a source of n-3 PUFA and therefore contributes to n-3 PUFA intake from food and supplements. Model 5 therefore addresses the question of whether higher n-3 PUFA intake from food and supplements is associated with CHD mortality among individuals consuming equal quantities of fish.

The above analyses relate to variables measured at DSA1 to the hazard of CHD mortality over a long period of follow-up (up to 22 years). The results are therefore likely to be

affected by changes in supplement use over time and the short-term effect of n-3 PUFA on arrhythmia [24]. We therefore repeated the analysis using time-updated measures of supplement use and covariates from DSA2 and DSA3 to reduce misclassification. We performed this analysis in two ways. First, in separate analyses using DSA1, DSA2 and DSA3 as the time origins (the first of these is the same as the analysis described above; however, all DSA used adjustment model 3 since dietary variables were not available for DSA2 and DSA3). Secondly, we performed a single analysis using the most up-to-date exposure and covariate measures for each individual at each time they were at risk, *i.e.* time-varying covariates modelling. These models used the full length of follow-up. Additionally, we restricted the follow-up time at each DSA to two and four years of follow-up as well as until the start of the next DSA (at which time participants were censored). We did this to acknowledge unobserved changes in individuals' supplement use over time which could result in biased associations over time. We equally applied the strategy described in this paragraph using the *change* between supplement group categories *between* DSA (DSA1-DSA2 and DSA2-DSA3) as the main exposure; the latter analyses were therefore restricted to participants having completed two consecutive DSA (Figure 1).

Using time-varying covariates modelling and maximum follow-up time, we explored potential effect modification using the main endpoint (CHD mortality mentioned anywhere) by including interaction terms of supplement use with sex, age, self-reported illnesses, smoking or BMI. The likelihood ratio tests were used to compare the models with and without the respective interaction term.

In sensitivity analyses, we investigated the use of different definitions for the CHD endpoint (as listed earlier) using time-varying covariates modelling and adjustment model 3.

There were missing data in the main exposure and in the covariates. For the descriptive statistics, multinomial logistic analyses and the main Cox regression analyses the missing data were handled as follows. At DSA1, participants with missing data on supplement use and covariables were excluded; at DSA2 and DSA3, missing data on time-dependent covariates, but not supplement use, were carried over from previous DSA where available, *e.g.* missing smoking status at DSA3 would take the value at DSA2. Since DSA2 was a postal data collection only, BMI was taken from the nearest health examination (HE2). Self-

reported prevalent illnesses were not asked for at the time of DSA3, hence data from DSA1 and DSA2 were carried over. The use of the 'last observation carried forward' to handle missing data in time-dependent covariates at DSA2 and DSA3 resulted in some data still being missing at these time points and these individuals were excluded from the main analyses. In the Cox regression analyses we also used multiple imputation as an alternative method for dealing with missing data; details of the approach taken are provided in Appendix IV.

The proportional hazards assumption was tested based on the time-varying covariates model. Significant interactions between time and age, sex and self-reported myocardial infarction were observed; however, inclusion of these variables with their time interaction terms resulted in negligible differences in the HR or 95%CI of the main exposure (supplement use). Results presented are therefore without these interaction terms in the regression equation.

Data were analysed using Stata v14.

Patient involvement

During the study, EPIC-Norfolk participants have received newsletters with summaries of publications and announcements regarding study planning and progression. Researchers have given talks in community groups about the study in general and its findings. Public events have included representation of the EPIC-Norfolk study at the Cambridge Science Festival, of which the resources have been made available to local schools: http://www.sciencefestival.cam.ac.uk/resources. In 2009, EPAP was established (EPIC-Norfolk Participant Advisory Panel). The contributions of this group of participants has varied from providing potential research topics and assessing the potential burden of research activities to clarifying (research) materials to be send to the cohort. This meant that questionnaires, newsletters and participant information sheets could be improved before being sent to our full cohort or to the ethics committee. Summaries of these meetings may be found on: http://www.srl.cam.ac.uk/epic/participant_panel.html.

Appendix II: Consistency of and change in supplement use from data collected at three time points in EPIC-Norfolk (1993-2011).

Below diagram includes all participants who consented and completed the dietary supplement assessments (DSA) from the three included time points (N=8252). The first subdivide in the diagram is the distribution of supplement use as reported at DSA1; the second at DSA2; the third at DSA3. The percentages represent the number relative to the total, indicating consistency over time; the top 3 'pathways' are marked in red.

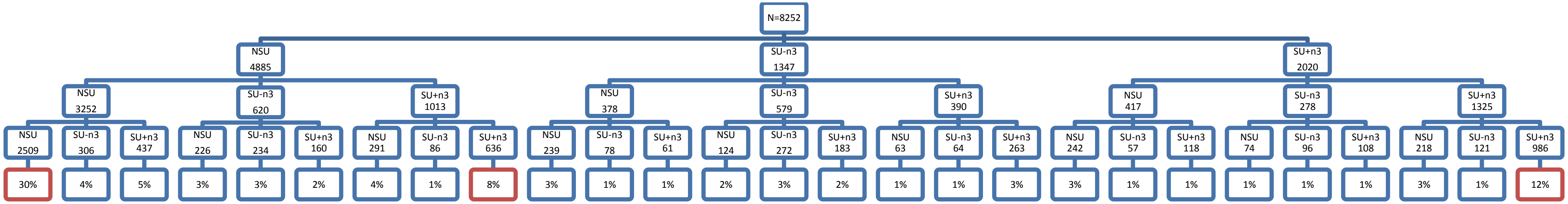

Appendix III: Fish or Supplement?

Characteristics of participants by quintiles of n-3 PUFA intake from food and supplements at DSA1 are provided in Appendix III-Table 1. Since fish is a source of n-3 PUFA, there was a strong association between fish consumption, particularly oily fish, and quintiles of n-3 PUFA intake from food and supplements. The percentage of fish consumers was 43% in quintile 1 and 98% in quintile 5.

It was observed that participants consuming a median intake of 0.81 g/d (5th quintile), had a 21% lower hazard of CHD mortality compared to very low consumers when adjusting for age and sex (Appendix III-Table 2); however, this association attenuated and became non-significant after adjusting for confounders and dietary variables, excluding fish (HR 0.93, 95%CI: 0.79, 1.09). When fish consumption was included, the HR for quintile 5 *vs.* 1 strengthened to 0.79 (95%CI: 0.64, 0.98). Equally, when the exposure was expressed as a continuous variable (per 1 g/d n-3 PUFA intake from food and supplements), we observed a stronger, borderline significant association when comparing model 4 (HR: 0.96, 95%CI: 0.85, 1.07) to model 5 (HR: 0.86, 95%CI: 0.74, 1.01).

In secondary analysis (Appendix III-Table 2), we excluded participants with prevalent baseline myocardial infarction, stroke and diabetes, by which potential increased fish consumption due to diagnosis could be dealt with. HRs strengthened and both quintile 4 (median of 0.33 g/d) and quintile 5 showed a significant lower hazard of CHD mortality compared to quintile 1.

In summary, an inverse association between quintiles of n-3 PUFA intake and CHD mortality was only observed after adjustment for fish consumption, indicating that food and supplement sources of n-3 PUFA might be differently associated with CHD mortality.

However, data on fish consumption (and hence n-3 PUFA intake from food) from 7-day diet diaries could only be estimated for DSA1. Within person changes in fish consumption were unknown and the lack of such data might have misclassified participants over time; or, our results might have been confounded if fish consumption over time was associated with risk factors of CHD mortality, *e.g.* a non-fatal myocardial infarction might have led to increased fish consumption, thereby associating higher fish or n-3 PUFA intake with higher risk of CHD mortality. This 'confounding by indication' might have been the reason why HRs between

n-3 PUFA intake from food and supplements and CHD mortality strengthened after excluding prevalent illnesses.

Fish consumption was encouraged (inter)nationally during the follow-up time [25–27]; however, national survey data do not indicate increased fish consumption [28,29], although a decrease in the duration of data collection from 7 to 4-day diary records limits this comparison.

Fish contains both nutrients and contaminants, such as methylmercury, dioxin or dioxin like polychlorinated biphenyls (PCB), which have been associated with the CHD, but in opposing directions [27,30–32]. Fish oil supplements are not free of such contaminants, but might contain lower concentrations because of their production process [33]. This might explain why adjusting for fish consumption, *i.e.* a relative larger proportion of the higher n-3 PUFA intake being derived from supplements, was associated with a lower hazard of CHD mortality and why fish was associated with a higher hazard of CHD mortality.

Fish consumption advice has been given which maximises n-3 PUFA intake, while minimising methylmercury content for the general population, since the lower hazard of CHD mortality observed for higher n-3 PUFA concentrations only became apparent after controlling for methylmercury contamination [30,32,34].

Appendix III-Table 1: Characteristics of participants by n-3 PUFA intake from food and supplement as assessed at DSA1 (1993-1998). Values are percent or Median (IQR).

Median n-3 PUFA from food and supplements (IQR), g/d	Quintile 1<br>0.04 (0.02, 0.05)	Quintile 2<br>0.09 (0.07, 0.10)	Quintile 3<br>0.16 (0.14, 0.19)	Quintile 4<br>0.33 (0.27, 0.40)	Quintile 5<br>0.81 (0.62, 1.17)
CHD mortality	287/4400	293/4417	338/4410	313/4410	331/4398
Crude rate (1000 PYAR)	3.728	3.809	4.413	4.087	4.335
Sex					
Men	40	47	45	44	49
Women	60	53	55	56	51
Age					
=<50 years	28	24	19	17	14
>50-60 years	33	32	33	30	31
>60-70 years	26	29	32	37	37
>70 years	14	15	17	16	18
BMI					
=<20 kg/m^2	3	2	2	2	2
>20-25 kg/m^2	40	37	38	37	39
>25-30 kg/m^2	41	46	46	47	47
>30 kg/m^2	16	15	15	15	12
Smoking status					
Current	14	13	11	9	8
Former	39	43	43	44	45
Never	47	45	46	47	47
Social class					
Professional	7	5	6	7	9
Managerial	34	35	36	38	41
Skilled non-manual	16	16	18	18	16
Skilled manual	25	25	23	22	20
Semi-skilled	14	15	14	12	11
Non-skilled	4	4	4	3	2

Median n-3 PUFA from food and supplements (IQR), g/d	Quintile 1<br>0.04 (0.02, 0.05)	Quintile 2<br>0.09 (0.07, 0.10)	Quintile 3<br>0.16 (0.14, 0.19)	Quintile 4<br>0.33 (0.27, 0.40)	Quintile 5<br>0.81 (0.62, 1.17)
Marital status					
Married	82	83	82	83	82
Not married	19	18	18	17	18
Education level					
No qualification	37	38	38	36	32
O-level	11	11	10	10	10
A-level	39	40	40	41	43
Degree	12	11	12	13	15
Season of 7dDD					
Spring	25	26	27	28	26
Summer	23	24	24	27	27
Autumn	29	26	25	24	25
Winter	24	24	23	21	23
Physical activity					
Inactive	30	31	30	31	28
Moderately inactive	28	28	30	29	30
Moderately active	23	23	23	22	24
Active	19	18	17	18	18
Alcohol intake (HLQ)					
None	16	14	14	13	11
>0-14 units/wk	73	73	72	71	71
>14-28 units/wk	8	10	11	12	14
>28 units/wk	3	4	3	4	4
Self-reported illness					
Myocardial infarction	3	4	3	3	4
Stroke	2	1	1	1	2
Diabetes	2	2	3	2	3

	Quintile 1	Quintile 2	Quintile 3	Quintile 4	Quintile 5
**Median n-3 PUFA from food and supplements (IQR), g/d**	**0.04 (0.02, 0.05)**	**0.09 (0.07, 0.10)**	**0.16 (0.14, 0.19)**	**0.33 (0.27, 0.40)**	**0.81 (0.62, 1.17)**
Energy (MJ/d)	7.61 (6.32, 9.06)	8.03 (6.74, 9.61)	8.08 (6.76, 9.63)	8.04 (6.80, 9.60)	8.28 (6.99, 9.84)
Protein (en%)	14.5 (13.0, 16.3)	15.0 (13.4, 16.7)	15.2 (13.7, 16.9)	15.4 (13.9, 17.1)	15.7 (14.3, 17.5)
Fat (en%)	33.1 (29.6, 36.7)	33.9 (30.2, 37.2)	33.6 (30.1, 37.0)	33.2 (29.5, 36.5)	33.2 (29.6, 36.8)
Saturated fat (en%)	12.9 (11.1, 14.7)	13.0 (11.2, 14.9)	12.8 (11.1, 14.7)	12.6 (10.8, 14.5)	12.3 (10.4, 14.2)
Carbohydrates (en%)	48.9 (44.6, 53.0)	47.7 (43.7, 51.4)	47.5 (43.3, 51.2)	47.3 (43.1, 51.0)	46.5 (42.1, 50.6)
Alcohol (en%)	1.3 (0.0, 5.0)	1.7 (0.0, 5.1)	2.0 (0.0, 5.7)	2.4 (0.0, 6.5)	2.7 (0.3, 6.9)
Food intake (g/d)					
Fruit	134 (64, 220)	136 (68, 218)	150 (79, 234)	162 (92, 253)	186 (109, 275)
Vegetables	132 (93, 177)	134 (96, 180)	137 (100, 184)	149 (108, 196)	159 (117, 212)
Red & processed meat	53 (31, 76)	58 (37, 81)	54 (34, 78)	51 (31, 73)	48 (26, 74)
White meat	18 (3, 34)	21 (7, 37)	21 (8, 38)	22 (8, 38)	21 (5, 39)
Oily fish	0 (0, 0)	0 (0, 4)	0 (0, 9)	15 (8, 22)	31 (21, 48)
Consumers (%)	15	30	49	85	93
Consumers only	7 (4, 11)	9 (5, 14)	9 (6, 15)	17 (12, 24)	34 (24, 50)
White fish	0 (0, 11)	16 (10, 22)	19 (6, 30)	17 (0, 28)	15 (0, 27)
Consumers (%)	32	80	78	75	68
Consumers only	11 (11, 16)	9 (15, 25)	21 (16, 34)	21 (15, 34)	21 (15, 33)
Dietary supplement					
NSU	79	75	51	54	45
SU-n3	19	16	12	14	13
SU+n3	2	9	37	31	42
n-3 PUFA (g/d)					
Food	0.04 (0.02, 0.05)	0.08 (0.07, 0.10)	0.14 (0.09, 0.17)	0.30 (0.24, 0.38)	0.66 (0.52, 0.90)
Food + supplement	0.04 (0.02, 0.05)	0.09 (0.07, 0.10)	0.16 (0.14, 0.19)	0.33 (0.27, 0.40)	0.81 (0.62, 1.17)
Ratio supplement/(food+supplement) (%)	0 (0, 0)	0 (0, 0)	0 (0, 48)	0 (0, 20)	0 (0, 16)

n-3 PUFA, marine omega-3 polyunsaturated fatty acids EPA and DHA; IQR, interquartile range; HLQ, health and lifestyle questionnaire.

Appendix III-Table 2: The association between quintiles of the sum of food and supplement n-3 PUFA at DSA1 and subsequent hazard of CHD mortality (where cause of death was mentioned anywhere on the death certificate). The analyses relied on a median of 19 years of follow-up (1993-2015).

	Quintile 1	Quintile 2	Quintile 3	Quintile 4	Quintile 5	Continuous
Median food+supplement n-3 PUFA (IQR) g/d	0.04 (0.02, 0.05)	0.09 (0.07, 0.10)	0.16 (0.14, 0.19)	0.33 (0.27, 0.40)	0.81 (0.62, 1.17)	**Per 1 g/d**
Median food n-3 PUFA (IQR) g/d	0.04 (0.02, 0.05)	0.08 (0.07, 0.10)	0.14 (0.09, 0.17)	0.30 (0.24, 0.38)	0.66 (0.52, 0.90)	
Median ratio food:(food+supplement) (IQR) %	100 (100, 100)	100 (100, 100)	100 (52, 100)	100 (79, 100)	100 (84, 100)	
Median white fish (IQR) g/d	0 (0, 11)	16 (10, 22)	19 (6, 30)	17 (0, 28)	15 (0, 27)	
Median oily fish (IQR) g/d	0 (0, 0)	0 (0, 4)	0 (0, 9)	15 (8, 22)	31 (21, 48)	
	**HR**	**HR (95% CI)**	**HR (95% CI)**	**HR (95% CI)**	**HR (95% CI)**	**HR (95% CI), P**
Case/n	287/4400	293/4417	338/4410	313/4410	331/4398	1562/22,035
Model 1: sex and age	1.00	0.91 (0.77, 1.07)	0.95 (0.81, 1.11)	0.83 (0.71, 0.97)	0.79 (0.68, 0.93)	0.87 (0.77, 0.97), P=0.013
Model 2: 1 + socio/lifestyle factors	1.00	0.91 (0.77, 1.07)	1.00 (0.85, 1.17)	0.89 (0.76, 1.04)	0.87 (0.74, 1.02)	0.92 (0.83, 1.03), P=0.17
Model 3: 2 + prevalent illnesses	1.00	0.89 (0.75, 1.05)	1.02 (0.87, 1.20)	0.91 (0.78, 1.07)	0.87 (0.74, 1.03)	0.92 (0.82, 1.03), P=0.15
Model 4: 3 + dietary factors (excluding fish)	1.00	0.91 (0.77, 1.07)	1.05 (0.90, 1.24)	0.96 (0.82, 1.13)	0.93 (0.79, 1.09)	0.96 (0.85, 1.07), P=0.45
Model 5: 4 + fish	1.00	0.88 (0.74, 1.04)	1.00 (0.85, 1.18)	0.88 (0.74, 1.05)	0.79 (0.64, 0.98)	0.86 (0.74, 1.01), P=0.07
Case/n	217/4149	212/4132	257/4128	231/41341	242/4090	1159/20,640
Model 5, excluding illness at DSA1	1.00	0.86 (0.71, 1.04)	0.96 (0.79, 1.16)	0.79 (0.64, 0.97)	0.70 (0.55, 0.90)	0.86 (0.71, 1.03), P=0.09

DSA, dietary supplement assessment; n-3 PUFA, omega-3 polyunsaturated fatty acids (sum of EPA and DHA); IQR, interquartile range; CHD, coronary heart disease; HR, hazard ratio; 95%CI, 95% confidence interval.

See also Appendix III-Table 1 for detailed description of the cohort by quintiles of n-3 PUFA exposure at DSA1.

Model 1: sex, age (continuous).
Model 2: Model 1 + smoking, BMI (continuous), alcohol intake, season in which 7dDD was completed, physical activity (inactive vs. some activity), social class (manual vs. non-manual), education level (no qualification vs. some qualification), marital status (not married vs. married).
Model 3: Model 2 + adjusted for prevalent diabetes, myocardial infarction and stroke.
Model 4: Model 3 + energy intake, fruit, vegetable, red & processed meat, white meat (all continuous).
Model 5: Model 4 + white fish (HR per 80 g/d: 1.20, 95%CI: 0.94, 1.52), oily fish (HR per 80 g/d: 1.33, 95%CI: 1.02, 1.72).
Model 5, excluding baseline myocardial infarction, stroke, diabetes: white fish (HR per 80 g/d: 1.23, 95%CI: 0.93, 1.61), oily fish (HR per 80 g/d: 1.66, 95%CI: 1.21, 2.27).

Appendix IV: Multiple Imputation

Multiple imputation was used to handle missing data in time-fixed variables and in longitudinal exposures. For the longitudinal variables, multiple imputation is a superior alternative to the last-observation carried forward approach used in the main analyses (Figure 1).

Appendix IV-Table 1 shows the number of missing values for each included variable in adjustment model 3. DSA1 had the highest number of variables with missing values, particularly for supplement use (10%), since supplement use was obtained as part of a short questionnaire on the last pages of the 7dDD booklet; participants who did not complete all seven days were most likely to skip these questions. At DSA2 and DSA3, BMI had the most missing values, 28% and 22% respectively. At DSA2, this was caused by BMI being obtained from an earlier time point than the questionnaire (with non-response for HE2, see Appendix I-Table 1); at DSA3, not all participants were seen at HE3 due to time/cost restrictions of the study.

Univariable logistic regression was applied to study associations between each variable and missingness in the supplement use variable (Appendix IV-Table 1). Only at DSA1 were significant associations observed between variables included in Model 3 of the analysis and missingness for supplement use. Being a man, an alcohol consumer, unmarried, smoker or heavier were associated with more missingness of supplement use; being older was associated with less missingness of supplement use. Dietary variables were only available at DSA1, these had strong associations with missingness for the supplement use variable

Missing supplement use at DSA1 (n=2619) was not associated with CHD mortality (HR=0.87; 95%CI: 0.74, 1.02). Only 4 and 11 participants had missing data on supplement use at DSA2 and DSA3 respectively.

Missing values in several variables were handled using multiple imputation by chained equations (MICE). We used the imputation approach described by White and Royston [35] for imputation of missing data in explanatory variables used in Cox regression, by including the event indicator and the Nelson-Aalen estimate of the cumulative hazard at the event or censoring time as predictors in all imputation models, in addition to the predictors summarised below. After the imputation, the aim is to estimate the association between

CLO group and the outcome (CHD mortality) using adjustment model 3. The time-fixed variables included in adjustment model 3 are sex, age, education, social class, and the time-dependent variables are supplement use, smoking, BMI, physical activity, alcohol consumption, season of questionnaire completion and marital status. Missing data in time-dependent variables was handled using a modified version of the two-fold fully conditional specification approach suggested by Nevalainen *et al.* [36]. Time-fixed variables were imputed using time-dependent variables measured at DSA1 as predictors. Time-dependent variables at DSA1 were imputed using the time-fixed variables and other time-dependent variables from DSA1 as predictors. A given time-dependent variable at DSA2 was imputed using the same variable from DSA1 as a predictor, alongside the other time-dependent variables from DSA2 and the time-fixed variables. Similarly, a given time-dependent variable at DSA3 was imputed using the same variable from DSA2 as a predictor, alongside the other time-dependent variables from DSA3 and the time-fixed variables. In summary, a given time-dependent variable was imputed using time-fixed variables, the most recent past value of the same variable, and concurrent values of other time-dependent variables, in addition to the event indicator and the Nelson-Aalen estimate of the cumulative hazard at the event or censoring time. Nevalainen *et al.* suggested including both past and future values of time-dependent variables in the imputation model for a given time-dependent variable [36]; however, this caused convergence issues in our setting and so we simplified the procedure to only use past values. The multiple imputation was performed using *mi impute* in Stata v14. Twenty imputed data sets were created (the percentage of participants with minimally one missing value was 14%, 30% and 25% at each DSA respectively) [37]. Imputed values were used in Cox regression models and the resulting estimated log hazard ratios were combined using Rubin's Rules. Cox proportional hazards analysis was applied to replicate results reported in Table 2 of the main manuscript (with CHD mortality reported on the death certificate [anywhere] as the outcome).

The results are shown in Appendix IV-Table 2. Results using the imputed dataset and results from the main manuscript had similar HRs. As expected, the confidence intervals obtained after multiple imputation were smaller than those obtained previously, and hence some borderline non-significant associations became borderline significant associations. Our

conclusions are therefore unchanged: supplements containing n-3 PUFA (mainly low dose cod liver oil in this cohort) were associated with a lower hazard of CHD mortality.

Appendix IV-Table 1: Number of missing values for each variable and the association (using univariable logistic regression) between this variable and missingness for the supplement use variable.

	DSA1		DSA2		DSA3	
	N	OR (95%CI)	N	OR (95%CI)	N	OR (95%CI)
Attended HE1	25,639					
Completed questionnaire			17,585		10,874	
Supplement use	2619	-	11	-	4	-
Social class	570	1.06 (0.97, 1.15)	313	1.21 (0.27, 5.43)	143	0.89 (0.08, 9.77)
Alcohol	268		156		282	
>0-14 units/wk		1.18 (1.03, 1.34)		0.48 (0.11, 2.12)		-
>14-28 units/wk		1.58 (1.34, 1.86)		2.03 (0.45, 9.08)		
>28 units/wk		1.71 (1.38, 2.14)		2.19 (0.24, 19.67)		
Smoking	220		91		4	-
current		1.95 (1.74, 2.19)		-		
former		1.06 (0.97, 1.16)		0.83 (0.12, 5.88)		
Marital status	150	1.12 (1.02, 1.24)	138	-	104	-
Seasons	132		0	-	0	-
Spring		0.97 (0.86, 1.09)				
Summer		0.90 (0.80, 1.02)				
Autumn		1.01 (0.89, 1.13)				
BMI (kg/m^2)	57	1.05 (1.04, 1.06)	4864	0.98 (0.83, 1.15)	2393	0.84 (0.60, 1.18)
Physical activity	19	0.97 (0.89, 1.06)	0	1.06 (0.31, 3.62)	1	-
Education	18	1.11 (1.02, 1.21)	6	0.33 (0.06, 1.99)	3	-
Self-reported						
MI	13	0.86 (0.67, 1.10)	0	-	0	-
diabetes	13	0.78 (0.58, 1.05)	0	1.92 (0.25, 15.4)	0	2.56 (0.32, 20.49)
stroke	13	1.06 (0.76, 1.48)	0	-	0	-
Sex	0	1.36 (1.25, 1.48)	0	1.59 (0.48, 5.20)	0	1.30 (0.18, 9.23)
Age	0	0.97 (0.97, 0.97)	0	1.04 (0.97, 1.11)	0	1.06 (0.95, 1.20)
Energy (MJ/d)	132	0.96 (0.95, 0.98)	-		-	
Foods (g/d)						
Oily fish	132	0.53 (0.44, 0.65)	-		-	
White fish	132	0.36 (0.29, 0.45)	-		-	
Red & processed meat	132	1.30 (1.19, 1.41)	-		-	
Fruit	132	0.83 (0.81, 0.86)	-		-	
Vegetables	132	0.88 (0.84, 0.92)	-		-	

Appendix IV-Table 2: The association between supplement use reported at DSA1, DSA2 and DSA3 and subsequent hazard of CHD mortality (where cause of death was mentioned anywhere on the death certificate). The follow-up time in the EPIC-Norfolk study was from 1993 to 2015. Results obtained from multiple imputation as described above (these results may be compared to Table 2 of the main manuscript).

		**Two years of follow-up**		**Four years of follow-up**		**Follow-up time until next DSA***		**Full follow-up time**	
	Total N	CHD Events N	HR (95% CI)	CHD Events N	HR (95% CI)	CHD Events N	HR (95% CI)	CHD Events N	HR (95%CI)
DSA1 (1993-1998)	25,621	99		234		1053		**1813	
NSU			1.00		1.00		1.00		1.00
SU-n3			1.18 (0.59, 2.35)		1.24 (0.80, 1.91)		1.04 (0.84, 1.27)		0.96 (0.82, 1.12)
SU+n3			0.92 (0.53, 1.58)		1.06 (0.76, 1.48)		0.84 (0.71, 0.99)		0.92 (0.81, 1.04)
DSA2 (2002-2004)	17,585	87		196		604		832	
NSU			1.00		1.00		1.00		1.00
SU-n3			0.98 (0.51, 1.88)		1.19 (0.79, 1.81)		0.94 (0.72, 1.21)		0.83 (0.66, 1.04)
SU+n3			0.54 (0.29, 0.98)		0.67 (0.46, 0.98)		0.74 (0.61, 0.91)		0.81 (0.69, 0.96)
DSA3 (2004-2011)	10,874	65		137		261			
NSU			1.00		1.00		1.00		
SU-n3			0.60 (0.26, 1.42)		1.12 (0.69, 1.81)		1.07 (0.74, 1.53)		
SU+n3			0.45 (0.23, 0.88)		0.58 (0.37, 0.89)		0.72 (0.53, 0.97)		
Time-varying	27,796	251		567		**1918			
NSU			1.00		1.00		1.00		
SU-n3			0.93 (0.61, 1.41)		1.19 (0.92, 1.53)		0.92 (0.79, 1.06)		
SU+n3			0.63 (0.45, 0.89)		0.78 (0.63, 0.97)		0.74 (0.66, 0.84)		

All models adjusted (model 3) for time-point specific (and imputed where necessary): age, smoking, BMI, alcohol consumption, physical activity, season of questionnaire completion, marital status and self-report of myocardial infarction, stroke or diabetes; as well as: sex, social class and education.
*or in case of DSA3, the censor date was the date of administrative follow-up (31 March 2015).
**Since participants can contribute follow-up time at DSA2 and/or DSA3 only (without contributing to DSA1), the number of participants/deaths included in the time-varying analysis is larger than reported in DSA1 only.

Appendix V: Characteristics of participants by type of supplement used as measured at DSA1 (1993-1998). Values are median (interquartile range) for continuous variables and % for categorical variables.

	Full cohort N=22,035	NSU N=13,444	SU-n3 N=3263	SU+n3 N=5328	P[a]
CHD mortality	7.1 (1562)	7.5 (1012)	5.5 (178)	7.0 (372)	
Sex					<0.001
Men	45 (9890)	50	31	40	
Women	55 (12,145)	50	70	60	
Age [b]					
=<50 years	20 (4496)	22	25	13	<0.001
>50-60 years	32 (6984)	32	33	31	
>60-70 years	32 (7058)	31	29	37	
>70 years	16 (3497)	16	13	18	
BMI [c]					
=<20 kg/m^2	2 (491)	2	4	2	<0.001
>20-25 kg/m^2	38 (8369)	36	44	40	
>25-30 kg/m^2	45 (9979)	46	41	46	
>30 kg/m^2	15 (3196)	16	11	13	
Smoking status					<0.001
Current	11 (2395)	13	10	7	
Former	43 (9426)	43	40	45	
Never	46 (10,214)	45	51	48	
Social class					<0.001
Professional	7 (1531)	7	8	6	
Managerial	37 (8048)	36	42	36	
Skilled non-manual	17 (3715)	16	18	19	
Skilled manual	23 (5055)	24	18	23	
Semi-skilled	13 (2910)	14	11	13	
Non-skilled	4 (776)	4	3	3	
Marital status					<0.001
Married	82 (18,127)	83	80	81	
Not married	18 (3908)	17	20	19	
Education level					<0.001
No qualification	36 (7999)	37	31	39	
O-level	10 (2282)	10	12	10	
A-level	41 (8955)	40	43	41	
Degree	13 (2799)	13	15	11	
Season of 7dDD					0.022
Spring	26 (5817)	27	25	27	
Summer	25 (5473)	25	25	24	
Autumn	26 (5661)	26	26	25	
Winter	23 (5084)	22	24	24	
Physical activity					<0.001
Inactive	30 (6592)	32	26	28	
Moderately inactive	29 (6389)	28	31	31	
Moderately active	23 (5040)	23	25	23	
Active	18 (4014)	18	19	19	

	Full cohort N=22,035	NSU N=13,444	SU-n3 N=3263	SU+n3 N=5328	P[a]
Alcohol intake (HLQ)					<0.001
None	13 (2930)	14	13	13	
>0-14 units/wk	72 (15,880)	71	74	74	
>14-28 units/wk	11 (2418)	11	10	11	
>28 units/wk	4 (807)	4	3	3	
Self-reported illness					
Myocardial infarction	3 (704)	4	2	2	<0.001
Stroke	1 (304)	2	1	1	0.005
Diabetes	2 (510)	3	2	2	<0.001
Energy (MJ/d)	8.00 (6.71, 9.56)	8.09 (6.72, 9.70)	7.77 (6.60, 9.19)	7.97 (6.77, 9.38)	<0.001
Protein (en%)	15.2 (13.6, 16.9)	15.1 (13.6, 16.9)	15.1 (13.5, 16.9)	15.4 (13.9, 17.0)	<0.001
Fat (en%)	33.4 (29.8, 36.8)	33.7 (30.2, 37.2)	33.0 (29.1, 36.4)	32.7 (29.3, 36.1)	<0.001
Saturated fat (en%)	12.7 (10.9, 14.6)	12.9 (11.1, 14.8)	12.5 (10.6, 14.5)	12.4 (10.6, 14.2)	<0.001
Carbohydrates (en%)	47.6 (43.4, 51.5)	47.2 (43.0, 51.1)	48.1 (44.0, 52.0)	48.2 (44.1, 52.0)	<0.001
Alcohol (en%)	2.0 (0, 5.9)	2.0 (0.0, 5.9)	2.1 (0.0, 5.9)	1.9 (0.0, 5.7)	0.62
Food intake (g/d)					
Fruit	153 (82, 242)	140 (71, 226)	170 (97, 258)	174 (102, 265)	<0.001
Vegetables	142 (102, 190)	138 (99, 186)	147 (105, 199)	146 (109, 195)	<0.001
Red & processed meat	53 (32, 76)	56 (35, 80)	45 (24, 67)	49 (29, 73)	<0.001
White meat	21 (6, 37)	21 (7, 37)	20 (7, 37)	21 (6, 38)	0.27
Oily fish, consumer (%)	54	52	58	58	<0.001
Consumers only	17 (10, 30)	17 (9, 30)	18 (10, 32)	19 (11, 32)	<0.001
White fish, consumer (%)	66	66	64	69	<0.001
Consumers only	19 (13, 29)	19 (14, 29)	19 (12, 29)	19 (14, 29)	0.021
Total fish, consumer (%)	84	83	85	86	<0.001
Consumers only	28 (17, 44)	27 (16, 43)	28 (17, 45)	30 (18, 46)	<0.001
Micronutrient intake					
Vitamin A (mcg/d), food	342 (232, 529)	350 (237, 542)	328 (222, 508)	330 (227, 507)	<0.001
Food + supplement	481 (278, 1047)	350 (237, 542)	578 (288, 1064)	1105 (914, 1523)	-
Vitamin D (mcg/d), food	2.78 (1.83, 4.21)	2.76 (1.80, 4.16)	2.67 (1.76, 4.16)	2.91 (1.94, 4.40)	<0.001
Food + supplement	3.70 (2.17, 6.04)	2.76 (1.80, 4.16)	4.19 (2.44, 6.37)	6.92 (5.09, 9.54)	-
Vitamin E (mcg/d), food	9.6 (7.4, 12.4)	9.5 (7.3, 12.4)	9.6 (7.4, 12.3)	9.8 (7.6, 12.6)	<0.001
Food + supplement	10.6 (7.9, 15.1)	9.5 (7.3, 12.4)	15.6 (10.3, 24.1)	13.1 (9.2, 19.1)	-
n-3 PUFA (g/d)					
Food	0.12 (0.06, 0.34)	0.12 (0.06, 0.31)	0.13 (0.06, 0.37)	0.14 (0.07, 0.39)	<0.001
Supplement	0 (0, 0)	0	0	0.09 (0.08, 0.15)	-
Food + supplement	0.16 (0.07, 0.40)	0.12 (0.06, 0.31)	0.13 (0.06, 0.37)	0.30 (0.17, 0.72)	-
Ratio supplement/ (food+supplement)	0 (0, 0)	0	0	0.46 (0.21, 0.69)	-

n-3 PUFA, omega-3 polyunsaturated fatty acids (sum of eicosapentaenoic acid and docosahexaenoic acid); NSU, non-supplement users; SU-n3, non-n-3 PUFA supplement users; SU+n3, n-3 PUFA supplement users (mainly cod liver oil).

[a] Differences between groups were tested using Chi-squared statistic (categorical variables) and Kruskal-Wallis (continuous variables).

[b] Median and Interquartile Range (IQR) of age (years) was: NSU 59 (51, 67), SU-n3 57 (50, 66), SU+n3 62 (54, 68); P<0.001.

[c] Median (IQR) of BMI (kg/m^2) was: NSU 26.0 (23.9, 28.5), SU-n3 25.2 (23.1, 27.6), SU+n3 25.6 (23.6, 28.0); P<0.001.

Appendix VI: Hazard ratios for CHD mortality by supplement user subgroups assessed at DSA1 only.

Here we use data from DSA1 only and we ignored any changes over time for any of the included variables in the models. This shows the results if only baseline study measures would have been used. Median follow-up time was 19 years (1993-2015). The hazard of CHD death among SU+n3 was 20% lower than among NSU after adjusting for sex and age (model 1). Following adjustment for CHD risk factors, the association attenuated and became non-significant after adjustment for self-reported illnesses (models 2 and 3). Further adjustment for dietary variables minimally changed this HR (models 4 and 5). Equally, there was no evidence that the CHD mortality of SU-n3 differed from NSU using data from DSA1 only.

	All Case/N	NSU HR (Ref)	SU-n3 HR (95% CI)	SU+n3 HR (95% CI)
Case/n	1562/22,035	1012/13,444	178/3263	372/5328
Model 1: sex and age		1.00	0.86 (0.73, 1.01)	0.80 (0.71, 0.90)
Model 2: 1 + socio/lifestyle factors		1.00	0.93 (0.79, 1.09)	0.87 (0.77, 0.98)
Model 3: 2 + prevalent illnesses		1.00	0.96 (0.82, 1.13)	0.94 (0.83, 1.06)
Model 4: 3 + dietary factors (excluding fish)		1.00	0.98 (0.83, 1.15)	0.97 (0.85, 1.09)
Model 5: 4 + fish		1.00	0.98 (0.83, 1.15)	0.96 (0.85, 1.09)

NSU, non-supplement users; SU-n3, non-n-3 PUFA supplement users; SU+n3, n-3 PUFA supplement users (mainly cod liver oil); PYAR, person years at risk; CHD, coronary heart disease; HR, hazard ratio; 95%CI, 95% confidence interval.

Model 1: sex, age (continuous).
Model 2: Model 1 + smoking, BMI (continuous), alcohol intake (categorical), season in which 7dDD was completed, physical activity (inactive vs. some activity), social class (manual vs. non-manual), education level (no qualification vs. some qualification), marital status (not married vs. married).
Model 3: Model 2 + prevalent diabetes, myocardial infarction, stroke.
Model 4: Model 3 + energy intake, fruit, vegetable, red & processed meat, white meat (all continuous).
Model 5: Model 4 + white fish, oily fish (all continuous).

Appendix VII: Interactions using time-varying covariate analysis for the association between supplement use and risk of CHD mortality (1993-2015).

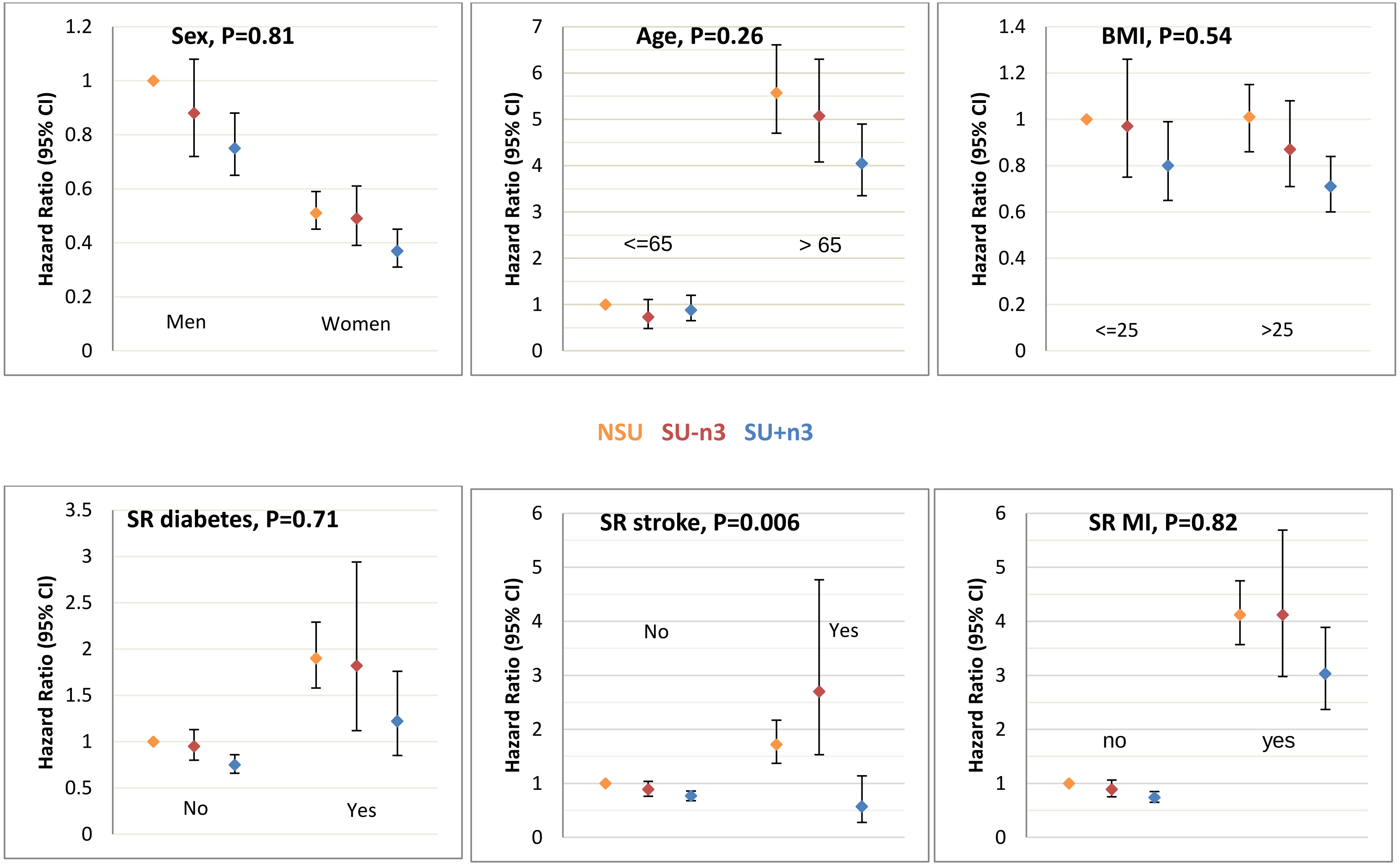

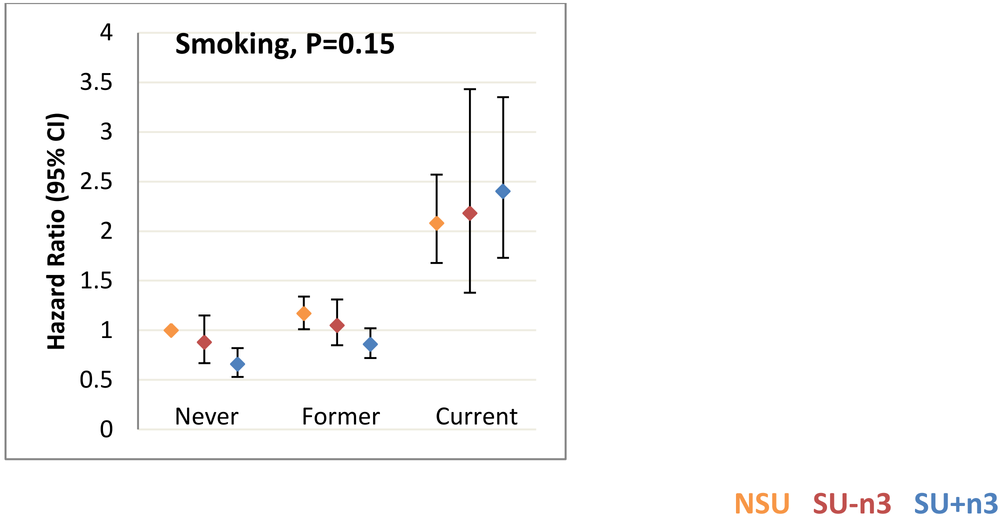

BMI, body mass index (kg/m^2); SR, self-reported; MI, myocardial infarction. ***Note***: the Y-axis for each graph is different.

All Models (1640/24,329) in time-varying covariates analysis were adjusted for: time-point specific age, smoking, BMI, alcohol consumption, physical activity, season of questionnaire completion, marital status and self-report of myocardial infarction, stroke or diabetes; as well as: sex, social class and education at time of DSA1.

The P-value represents the significance of the model's improvement after including the interaction term with supplement use and the presented variable.

References in Appendix

1 Day N, Oakes S, Luben R, *et al.* EPIC-Norfolk: study design and characteristics of the cohort. European Prospective Investigation of Cancer. *Br J Cancer* 1999;**80 Suppl 1**:95–103.

2 Hayat SA, Luben R, Keevil VL, *et al.* Cohort profile: A prospective cohort study of objective physical and cognitive capability and visual health in an ageing population of men and women in Norfolk (EPIC-Norfolk 3). *Int J Epidemiol* 2014;**43**:1063–72.

3 Lentjes MAH, Bhaniani A, Mulligan A a, *et al.* Developing a database of vitamin and mineral supplements (ViMiS) for the Norfolk arm of the European Prospective Investigation into Cancer (EPIC-Norfolk). *Public Health Nutr* 2011;**14**:459–71.

4 European Commission. Directive 2002/46/EC of the European Parliament and of the Council of 10 June 2002 on the approximation of the laws of the Member States relating to food supplements. *Off J Eur Communities* 2002;**L183/51**.

5 Lentjes MAH, Mulligan AA, Welch AA, *et al.* Contribution of cod liver oil-related nutrients (vitamins A, D, E and eicosapentaenoic acid and docosahexaenoic acid) to daily nutrient intake and their associations with plasma concentrations in the EPIC-Norfolk cohort. *J Hum Nutr Diet* 2015;**28**:568–82. doi:10.1111/jhn.12271

6 Bingham SA, Welch AA, McTaggart A, *et al.* Nutritional methods in the European Prospective Investigation of Cancer in Norfolk. *Public Health Nutr* 2001;**4**:847–58.

7 Welch AA, McTaggart A, Mulligan AA, *et al.* DINER (Data Into Nutrients for Epidemiological Research) - a new data-entry program for nutritional analysis in the EPIC-Norfolk cohort and the 7-day diary method. *Public Heal Nutr* 2001;**4**:1253–65.

8 Lentjes MAH, McTaggart A, Mulligan AA, *et al.* Dietary intake measurement using 7 d diet diaries in British men and women in the European Prospective Investigation into Cancer-Norfolk study: a focus on methodological issues. *Br J Nutr* 2014;**111**:516–26.

9 Holland B, Welch AA, Unwin D, *et al. McCance and Widdowson's The Composition of Foods*. Cambridge: : Royal Society of Chemistry (RSC) 1991.

10 Holland B, Unwin I, Buss D. *Cereals and cereal products. The third supplement to McCance & Widdowson's The Composition of Foods (4th Edition)*. Cambridge: : RSC/MAFF 1988.

11 Holland B, Unwin I, Buss D. *Milk products and eggs. The fourth supplement to McCance & Widdowson's The Composition of Foods (4th Edition)*. Cambridge: : RSC/MAFF 1989.

12 Holland B, Unwin I, Buss D. *Fruit and nuts. The first supplement to McCance & Widdowson's The Composition of Foods (5th Edition)*. Cambridge: : RSC/MAFF 1992.

13 Holland B, Unwin I, Buss D. *Vegetables, herbs and spices. The fifth supplement to McCance & Widdowson's The Composition of Foods (4th Edition)*. Cambridge: : RSC/MAFF 1991.

14 Holland B, Welch A, Buss D. *Vegetable dishes. The second supplement to McCance & Widdowson's The Composition of Foods (5th Edition)*. Cambridge: : RSC/MAFF 1992.

15 Holland B, Brown J, Buss D. *Fish and fish products. The third supplement to McCance & Widdowson's The Composition of Foods (5th Edition)*. Cambridge: : RSC/MAFF 1993.

16 Chan W, Brown J, Buss D. *Miscellaneous foods. The fourth supplement to McCance & Widdowson's The Composition of Foods (5th Edition)*. Cambridge: : RSC/MAFF 1994.

17 Chan W, Brown J, Lee S, *et al. Meat, poulry and game. The fifth supplement to McCance & Widdowson's The Composition of Foods (5th Edition)*. Cambridge: : RSC/MAFF 1995.

18 Chan W, Brown J, Church S, *et al. Meat products and dishes. The sixth supplement to McCance & Widdowson's The Composition of Foods (5th Edition)*. Cambridge: : RSC/MAFF 1996.

19 Ministry of Agriculture Fisheries and Food. *Fatty aids. The seventh supplement to McCance & Widdowson's The Composition of Foods (5th Edition)*. Cambridge: : RSC/MAFF 1998.

20 Mulligan AA, Kuhnle GGC, Lentjes MAH, *et al.* Intakes and sources of isoflavones, lignans, enterolignans, coumestrol and soya-containing foods in the Norfolk arm of the European Prospective Investigation into Cancer and Nutrition (EPIC-Norfolk), from 7 d food diaries, using a newly updated database. *Public Health Nutr* 2013;**16**:1454–62.

21 Welch AAA, Shakya-Shrestha S, Lentjes MAH, *et al.* Dietary intake and status of n-3 polyunsaturated fatty acids in a population of fish-eating and non-fish-eating meat-eaters, vegetarians, and vegans and the product-precursor ratio [corrected] of α-linolenic acid to long-chain n-3 polyunsaturated fatty ac. *Am J Clin Nutr* 2010;**92**:1040–51.

22 Shohaimi S, Luben R, Wareham N, *et al.* Residential area deprivation predicts smoking habit independently of individual educational level and occupational social class. A cross sectional study in the Norfolk cohort of the European Investigation into Cancer (EPIC-Norfolk). *J Epidemiol Community Health* 2003;**57**:270–6.
23 Wareham NJ, Jakes RW, Rennie KL, *et al.* Validity and repeatability of a simple index derived from the short physical activity questionnaire used in the European Prospective Investigation into Cancer and Nutrition (EPIC) study. *Public Health Nutr* 2003;**6**:407–13.
24 Mozaffarian D, Rimm EB. Fish intake, contaminants, and human health: evaluating the risks and the benefits. *JAMA* 2006;**296**:1885–99.
25 James MJ, Sullivan TR, Metcalf RG, *et al.* Pitfalls in the use of randomised controlled trials for fish oil studies with cardiac patients. *Br J Nutr* 2014;**112**:812–20.
26 Wu JHY, Mozaffarian D. ω-3 Fatty acids, atherosclerosis progression and cardiovascular outcomes in recent trials: new pieces in a complex puzzle. *Heart* 2014;**100**:530–3.
27 Scientific Advisory Committee Nutrition (SACN), Committee on Toxicity. Advice on fish consumption: benefits & risks. London: : The Stationery Office (TSO) 2004.
28 Henderson L, Gregory J, Swan G. The National Diet & Nutrition Survey: adults aged 19 to 64 years. Types and quantities of foods consumed. London: : Her Majesty's Stationery Office (HMSO) 2002.
29 Bates B, Lennox A, Prentice A, *et al.*, editors. *National Diet and Nutrition Survey Results from Years 1 , 2 , 3 and 4 (combined) of the Rolling Programme (2008/2009-2011/2012)*. London: : Public Health England 2014.
30 Stern AH. Public health guidance on cardiovascular benefits and risks related to fish consumption. *Environ Health* 2007;**6**:31.
31 Rissanen T, Voutilainen S, Nyyssönen K, *et al.* Fish oil-derived fatty acids, docosahexaenoic acid and docosapentaenoic acid, and the risk of acute coronary events: the Kuopio ischaemic heart disease risk factor study. *Circulation* 2000;**102**:2677–9.
32 Guallar E, Sanz-Gallardo MI, van't Veer P, *et al.* Mercury, fish oils, and the risk of myocardial infarction. *N Engl J Med* 2002;**347**:1747–54.
33 Bourdon JA, Bazinet TM, Arnason TT, *et al.* Polychlorinated biphenyls (PCBs) contamination and aryl hydrocarbon receptor (AhR) agonist activity of Omega-3 polyunsaturated fatty acid supplements: implications for daily intake of dioxins and PCBs. *Food Chem Toxicol* 2010;**48**:3093–7.
34 Mahaffey KR, Sunderland EM, Chan HM, *et al.* Balancing the benefits of n-3 polyunsaturated fatty acids and the risks of methylmercury exposure from fish consumption. *Nutr Rev* 2011;**69**:493–508.
35 White IR, Royston P. Imputing missing covariate values for the Cox model. *Stat Med* 2009;**28**:1982–98.
36 Nevalainen J, Kenward MG, Virtanen SM. Missing values in longitudinal dietary data: a multiple imputation approach based on a fully conditional specification. *Stat Med* 2009;**28**:3657–69.
37 White IR, Royston P, Wood AM. Multiple imputation using chained equations: Issues and guidance for practice. *Stat Med* 2011;**30**:377–99.
