# Supplementary material for: Longitudinal associations between marine omega-3 supplement users and coronary heart disease in a UK population-based cohort
Source: BMJ Open. 2017 Oct 13;7(10):e017471. doi: 10.1136/bmjopen-2017-017471 (PMC5652534; doi:10.1136/bmjopen-2017-017471)
Supplement: Author's manuscript [file bmjopen-2017-017471.draft_revisions.pdf]

BMJ Open

BMJ Open is committed to open peer review. As part of this commitment we make the peer review history of every article we publish publicly available.

When an article is published we post the peer reviewers' comments and the authors' responses online. We also post the versions of the paper that were used during peer review. These are the versions that the peer review comments apply to.

The versions of the paper that follow are the versions that were submitted during the peer review process. They are not the versions of record or the final published versions. They should not be cited or distributed as the published version of this manuscript.

BMJ Open is an open access journal and the full, final, typeset and author-corrected version of record of the manuscript is available on our site with no access controls, subscription charges or pay-per-view fees (http://bmjopen.bmj.com).

If you have any questions on BMJ Open's open peer review process please email editorial.bmjopen@bmj.com

BMJ Open

Longitudinal associations between n-3 containing PUFA supplements and Coronary Heart Disease in a UK population-based cohort

Journal:	*BMJ Open*
Manuscript ID	bmjopen-2017-017471
Article Type:	Research
Date Submitted by the Author:	24-Apr-2017
Complete List of Authors:	Lentjes, Marleen; University of Cambridge, Department of Public Health & Primary Care<br>Keogh, Ruth; London School of Hygiene and Tropical Medicine, Medical Statistics<br>Welch, A. A.; University of East Anglia, Population Health & Primary Care<br>Mulligan, Angela; University of Cambridge, Public Health & Primary Care<br>Luben, Robert; University of Cambridge, Department of Public Health and Primary Care<br>Wareham, Nicholas; University of Cambridge School of Clinical Medicine, MRC Epidemiology Unit<br>Khaw, Kay-Tee; University of Cambridge, Clinical Medicine
**Primary Subject Heading**:	Epidemiology
Secondary Subject Heading:	Nutrition and metabolism, Public health, Research methods, Cardiovascular medicine
Keywords:	EPIDEMIOLOGY, Cardiac Epidemiology < CARDIOLOGY, NUTRITION & DIETETICS

SCHOLARONE™
Manuscripts

Longitudinal associations between n-3 containing PUFA supplements and Coronary Heart Disease in a UK population-based cohort

Marleen AH Lentjes*: Snr. Research Nutritionist, Department of Public Health and Primary Care, University of Cambridge, Strangeways Research Laboratories, Worts Causeway, CAMBRIDGE CB1 8RN, United Kingdom. Email: marleen.lentjes@phpc.cam.ac.uk. Telephone: 01223 748683

Ruth H Keogh: Associate Professor, Department of Medical Statistics, London School of Hygiene and Tropical Medicine, Keppel street, LONDON WC1E 7HT, United Kingdom.

Ailsa A Welch: Reader in Nutritional Epidemiology, Department of Population Health & Primary Care, University of East Anglia, NORWICH NR4 7TJ, United Kingdom.

Angela A Mulligan: Snr. Research Nutritionist, Department of Public Health and Primary Care, University of Cambridge, Strangeways Research Laboratories, Worts Causeway, CAMBRIDGE CB1 8RN, United Kingdom.

Robert N Luben: Head of Bioinformatics, Department of Public Health and Primary Care, University of Cambridge, Strangeways Research Laboratories, Worts Causeway, CAMBRIDGE CB1 8RN, United Kingdom.

Nicholas J Wareham: Director MRC Epidemiology Unit, University of Cambridge, Institute of Metabolic Science, CAMBRIDGE CB2 0SL, United Kingdom.

Kay-Tee Khaw: Professor of Clinical Gerontology, University of Cambridge, School of Clinical Medicine, Addenbrooke's Hospital, CAMBRIDGE CB2 2QQ, United Kingdom.

*Corresponding author

For peer review only

Contributorship statement

MAHL: wrote the manuscript, prepared & analysed both the supplement and dietary data. Developed the research question.

RHK: advised on statistical analysis, specifically time-varying covariates analysis and multiple imputation.

AAW: Managed the dietary data collection of the study. Contributed to the study protocol.

AAM: Prepared dietary data.

RNL: Manages study data. Contributed to the study protocol.

NJW: Principal investigator of the study.

KTK: Principal investigator of the study.

All authors critically read and contributed to the manuscript.

Declaration of competing interests

All authors have completed the ICMJE uniform disclosure form at http://www.icmje.org/coi_disclosure.pdf and declare: no support from any organisation for the submitted work; no financial relationships with any organisations that might have an interest in the submitted work in the previous three years, no other relationships or activities that could appear to have influenced the submitted work.

Transparency declaration

Prof. Khaw affirms that the manuscript is an honest, accurate, and transparent account of the study being reported; no important aspects of the study have been omitted; any discrepancies from the study have been explained.

All authors had full access to all of the data (including statistical reports and tables) in the study and can take responsibility for the integrity of the data and the accuracy of the data analysis.

For peer review only - http://bmjopen.bmj.com/site/about/guidelines.xhtml

For peer review only

Ethics

Study approval was obtained from the Norfolk District Health Authority Ethics Committee. All participants completed a written informed consent.

Funding

All authors report grants from Cancer Research UK (G9502233) and grants from the Medical Research Council (MRC) during the study (SP2024-0201, SP2024-0204). RHK is supported by a MRC Fellowship (MR/M014827/1).

The funders of EPIC-Norfolk had no role in the study design, data collection, data analysis, interpretation of data, writing of the report, or in the decision to submit the paper for publication.

Data sharing statement

More information on the EPIC-Norfolk study can be obtained from the website: www.epic-norfolk.org.uk. Requests for collaboration will be discussed at the EPIC-Norfolk management meetings.

Exclusive license

The Corresponding Author has the right to grant on behalf of all authors and does grant on behalf of all authors, a worldwide licence (http://www.bmj.com/sites/default/files/BMJ%20Author%20Licence%20March%202013.doc) to the Publishers and its licensees in perpetuity, in all forms, formats and media (whether known now or created in the future), to i) publish, reproduce, distribute, display and store the Contribution, ii) translate the Contribution into other languages, create adaptations, reprints, include within collections and create summaries, extracts and/or, abstracts of the Contribution and convert or allow conversion into any format including without limitation audio, iii) create any other derivative work(s) based in whole or part on the on the Contribution, iv) to exploit all subsidiary rights that currently exist or as may exist in the future in the Contribution, v) the inclusion of electronic links from the Contribution to third party material where-ever it may be located; and, vi) licence any third party to do any or all of the above.

For peer review only

Acknowledgements

The authors thank the EPIC-Norfolk participants and the research and administrative staff, past & present.

For peer review only

For peer review only - http://bmjopen.bmj.com/site/about/guidelines.xhtml

Abstract

Objectives: Assess the association between n-3 PUFA intake from supplements (mainly cod liver oil) and CHD mortality.

Design: Prospective cohort study, with three exposure measurements over 22 y.

Setting: Norfolk-based European Prospective Investigation into Cancer (EPIC-Norfolk, UK).

Participants: 22,035 men and women from the general population, 39-79 y at recruitment.

Exposure: Supplement use was assessed in three questionnaires (1993-1998; 2002-2004; 2004-2011). Participants were grouped into non-supplement users (NSU), n-3 PUFA-containing supplement users (SU+n3) and non n-3 PUFA-containing supplement users (SU-n3). In Cox proportional hazards analysis, estimates were adjusted for the following time-point specific variables: age, smoking, prevalent illnesses, BMI, alcohol consumption, physical activity and season and the baseline assessments of sex, social class, education and dietary intake (7-day diet diary).

Primary and secondary outcome measures: During a median of 19 y follow-up, 1562 CHD deaths were registered for 22,035 included participants.

Results: Baseline n-3 PUFA supplement consumption was not associated with CHD mortality, but baseline food and supplement intake of n-3 PUFA was inversely associated with CHD mortality after adjusting for fish consumption. Using time-varying covariate analysis, significant associations were observed for SU+n3 (HR: 0.74, 95% CI: 0.66, 0.84), but not for SU-n3 vs. NSU. Those who became SU+n3 over time or were consistent SU+n3 vs. consistent NSU had a lower hazard of CHD mortality; no association with CHD was observed in those who stopped using n-3 PUFA-containing supplements. No associations were observed for first hospitalisation due to CHD.

Conclusions: Recent use of n-3 PUFA supplements was associated with a lower hazard of CHD mortality in this UK general population with low fish consumption. Residual confounding cannot be excluded, but associations observed were consistent with the postulated antiarrhythmic effect of low dose n-3 PUFA.

For peer review only

For peer review only - http://bmjopen.bmj.com/site/about/guidelines.xhtml

Article summary

Strengths and limitations of this study

- The use of dietary supplements was measured three times over the course of 22 years.
- The repeated measures enabled to study change in supplement use in relation to hard endpoints.
- The cohort was population-based as opposed to trials on omega-3 supplement use which have been in high risk populations.
- Total omega-3 intake from both food (mainly fish sources) and supplement sources could be quantified; however, only for the baseline measure.
- Estimates were adjusted for socio-demographic and other behavioural risk factors, although residual confounding cannot be excluded.

For peer review only

For peer review only - http://bmjopen.bmj.com/site/about/guidelines.xhtml

Introduction

The traditionally high omega-3 polyunsaturated fatty acid (n-3 PUFA) intake among the Inuit has been associated with a lower risk of ischemic heart disease (IHD) mortality in this population [1]. Research leading on from this observed that in a Dutch cohort, where average fish consumption was 20 g/d, higher fish consumption was associated with lower relative risk of Coronary Heart Disease (CHD) mortality between 1960-1980 [2]. A recent review of prospective cohort studies has observed a 16% (95%CI: 5-25%) lower risk of CHD mortality in those consuming one portion of fish per week *vs.* lower frequency of consumption; however, null findings among included studies were equally observed [3]. The association between fish intake and CHD has been attributed to n-3 PUFA, which are mainly derived from oily fish. According to the National Diet and Nutrition Survey in 2000/'01, less than 50% of the UK population consumed oily fish once a week [4]; however, dietary supplements may provide 50% of the n-3 PUFA intake [5].

N-3 PUFA at pharmaceutical doses reduce plasma triglycerides and possibly thrombosis; whereas lower doses (around 250 mg/d, obtainable from fish consumption and low dose supplements such as cod liver oil [CLO]) have been observed to lower the risk of sudden death or CHD death in trials and observational studies, which may be due to the influence of n-3 PUFA on heart rate and arrhythmia [6–8]. Reviews of trials on n-3 PUFA supplementation in relation to overall cardiovascular diseases (CVD) [9], or when separated into subgroups of stroke and coronary diseases [10–12], have shown no overall benefit, particularly in the most recent trials. It has been suggested that background medication, inclusion of non-fatal events as outcome measures and increasing trends in n-3 PUFA/fish consumption might underlie the null findings [13,14].

Although there have been a number of studies on the association between n-3 PUFA intake and CHD, the evidence for a protective association between n-3 PUFA supplement intake and CHD remains inconclusive [15]. Consumption of n-3 PUFA supplements is traditionally high in Northern European countries [16]. A Swedish cohort observed no association for fish oil supplements and CVD mortality [17]; whereas a study based in Iceland observed a lower risk of CHD hospitalisation with high frequency of CLO consumption [18]. Previous studies have relied on single baseline measures of supplement use and covariates [19],

For peer review only - http://bmjopen.bmj.com/site/about/guidelines.xhtml

excluded fish oil supplement users because of minimal use in the studied cohort [20] or lacked detailed data on supplement use and imputed n-3 PUFA content [21]; moreover, trials have included participants at increased risk of CHD [22–24] or included those who have already experienced a CHD event [25–28]; the effects of n-3 PUFA supplementation in a general population are less well documented, and the first primary prevention trial is ongoing [29].

The aim of this analysis was to use three repeated measures of supplement use to investigate the association between n-3 PUFA supplement intake and CHD mortality in a general population-based cohort.

Methods

An extended version of the methods has been included in Appendix I.

Study design

Recruitment for the Norfolk-based European Prospective Investigation into Cancer and Nutrition (EPIC-Norfolk) started in 1993 and was completed in 1998 [30,31]. Thirty-five general practices in the Norfolk area of East-Anglia (UK) took part in the study; 77,630 registered patients were approached, 30,445 consented. Exposures have been re- assessed over the course of the study; participants completed general Health and Lifestyle Questionnaires (HLQ) and attended three health examinations (HE) up to 2011 (Figure 1, Appendix I). Data on health outcomes and mortality were collected passively. Ethical approval for the study was given by the Norwich District Health Authority Ethics Committee. Participants gave written informed consent.

Assessment of n-3 PUFA supplement use

Dietary supplements were defined according to the EU directive 2002/46/EC [32]. Prescribed medication containing minerals and/or vitamins were not considered supplements. The sum of eicosapentaenoic acid (EPA) and docosahexaenoic acid (DHA) from supplements were the main exposure, referred to as n-3 PUFA. We used data from three dietary supplement assessments (DSA), named DSA1, DSA2, DSA3. All covered a one week recall (Appendix I).

For peer review only

For peer review only - http://bmjopen.bmj.com/site/about/guidelines.xhtml

At each DSA, participants were grouped by the type of supplement they consumed:

- non-supplement users (NSU);
- non n-3 PUFA supplement users (SU-n3): participants who consumed one or more supplements that did not contain n-3 PUFA;
- n-3 PUFA supplement users (SU+n3): participants who used n-3 PUFA supplements, either singly or in combination with other non n-3 PUFA supplements.

Participants were also grouped into five categories identifying change in n-3 PUFA supplement use between two consecutive DSA (Appendix I and Appendix II): 'consistent NSU', 'was SU+n3', 'became SU+n3', 'consistent SU+n3', 'other SU'.

Average daily nutrient composition from supplements was calculated using the Vitamin and Mineral Supplement (ViMiS) system [33] and added to the average daily food intake to obtain average total nutrient intake. Validation of these data with biomarkers were undertaken [5].

Dietary covariates

The analyses adjust for potential dietary confounders measured at DSA1. Diet was assessed using a 7-day diet diary (7dDD) between 1993-1998 [34]. Data were entered by trained staff into DINER [35] and checked and calculated by nutritionists using DINERMO [36]. The underlying food composition tables were derived from McCance & Widdowson's Composition of Foods and supplements [37]. Missing values for fatty acids were further completed using a recipe calculation system [38,39]. The baseline 7dDD provided data on average daily energy intake (MJ/d), proportion of energy provided by macronutrients (%En), the sum of EPA and DHA intake (g/d) referred to as 'n-3 PUFA' and consumption of the disaggregated amounts of fruit, vegetable, red meat, processed meat, white meat, and white and oily fish consumption (g/d) [36].

Assessment of other covariates

Social class was measured through occupational status (professional, managerial, skilled non-manual, skilled manual, semi-skilled, non-skilled). Highest education level achieved was divided in four categories (no qualification, O-level, A-level, degree). Both variables were

For peer review only - http://bmjopen.bmj.com/site/about/guidelines.xhtml

derived from HLQ1 (coinciding with DSA1) and are time-fixed. The remaining covariates are time-dependent and refer to measures obtained at time points corresponding to the DSA (Appendix I). We identified participants at higher risk of CHD using responses to the questions “Has the doctor ever told you that you have [myocardial infarction/diabetes/stroke]?”. Participants were classified as a never smoker, former smoker or current smoker. Alcohol consumption was classified as none, >0-14, >14-28, >28 units/wk. Marital status was re-categorised into married (married/living as married) or non-married (widowed, divorced, separated, single). Participants were classified as being active, moderately active, moderately inactive or inactive. BMI (kg/m^2) was calculated from height and weight measured by a trained nurse.

Participant selection, case ascertainment and outcomes studied

Participants were eligible for analyses if they provided data on supplement use at any of the three time points (DSA1, DSA2, DSA3) and attended the corresponding HE (Figure 1); 4030 participants were not included at any time point studied. The participant’s National Health Services number (a unique national patient identifier) was linked to the data from the Office of National Statistics to obtain vital status and causes of death. A similar procedure was followed for causes of hospital admissions registered by the East-Norfolk Primary Health Care Trust which records for its residents the admissions in England and Wales. The main endpoint studied was CHD mortality mentioned anywhere on the death certificate, identified with ICD9 410-414 or ICD10 I20-I25. In sensitivity analyses, we investigated the use of different definitions for the CHD endpoint: (i) CHD mortality mentioned as underlying cause of death, (ii) death due to acute myocardial infarction (ICD-codes 410 or I21), (iii) first recorded hospitalisation due to CHD.

Statistical analysis

A description of the cohort at DSA1, DSA2 and DSA3 is given. To study potential changes in supplement user characteristics over time, a multinomial logistic regression analysis was performed at each DSA with supplement use group as the outcome variables and the variables noted above as explanatory variables.

For peer review only

For peer review only - http://bmjopen.bmj.com/site/about/guidelines.xhtml

The main analyses used Cox regression, using the follow-up time scale. Individuals who died from causes other than the event of interest were censored at their date of death and individuals who did not die from any cause were censored at the end of follow-up (31st March 2015).

First, we modelled associations between supplement use at DSA1 and CHD mortality, with adjustment for covariates measured at DSA1. A series of *cumulative* adjustment models were used: sex and age-adjusted estimates (model 1); including smoking, BMI (kg/m^2), physical activity, alcohol intake, social class, education and season in which the questionnaire was completed (model 2); including self-reported myocardial infarction, stroke or diabetes (model 3); including energy intake (MJ/d) and disaggregated fruit (g/d), vegetable (g/d), red meat & processed meat (g/d), and white meat (g/d) (model 4); including white fish (g/d) and oily fish (g/d) (model 5). Secondly, models 1-5 were fitted using quintiles of n-3 PUFA intake from food and supplements at DSA1 as the main exposure (Appendix V).

The above analyses were repeated using time-updated measures of supplement use and covariates using model 3. First, we performed separate analyses using DSA1, DSA2 and DSA3 as the time origins. Secondly, we performed time-varying covariates modelling using the full length of follow-up as well as shorter length of follow-up time to acknowledge unobserved changes in individuals' supplement use over time. We equally applied the strategy described here using the *change* between supplement group categories *between* DSA.

We assessed interactions between supplement use and sex, age, self-reported illnesses, smoking and BMI using likelihood ratio tests.

At DSA1, participants with missing data on supplement use and covariables were excluded; at DSA2 and DSA3 missing covariates from the last observation were carried forward (Figure 1). Alternatively, multiple imputation was used to handle missing data in the Cox regression analyses (Appendix VII). The proportional hazards assumption was assessed in the time-varying covariates model, by including interactions between time and each explanatory variable. Data were analysed using Stata v14.

For peer review only - http://bmjopen.bmj.com/site/about/guidelines.xhtml

For peer review only

Results

Analyses were based on 22,035 participants who provided data at DSA1, 15,937 participants at DSA2 and 10,120 participants at DSA3 (Figure 1). Median (IQR) of follow-up time was 19 (17, 20) years. During this time, 1562 participants (out of 22,035) died of CHD mentioned *anywhere* on the death certificate.

Description of included participants

Table 1 describes the study participants at each DSA. Included participants had a median age of 59, 65 and 69 years at DSA1, DSA2 and DSA3 respectively and consisted of 45%, 43% and 44% men. We observed a lower smoking prevalence, fewer alcohol consumers and more physical inactivity as time progressed; however, there was still a large range in these behaviours. The proportion of NSU dropped from 61% at DSA1 to 50% at DSA3, with 24%, 31% and 34% using n-3 PUFA containing supplements at the three DSA respectively. The change in supplement use over time is depicted in Appendix II.

Multinomial logistic regression analysis assessed the relation of these characteristics to supplement use status, mutually adjusted, at DSA1, DSA2 and DSA3 (Figure 2). Having a non-manual social class and a higher education level were associated with SU-n3; whereas older participants, non-smoking, alcohol consumption and fewer self-reported illnesses were associated with SU+n3. Associations between covariates and supplement use group were similar at the three DSA.

n-3 PUFA supplement use and hazard of CHD mortality

There was no evidence that the CHD mortality of SU+n3 or SU-n3 differed from NSU when solely using baseline data (Appendix IV). Table 2 shows results from the Cox proportional hazards analyses in which the time origin was re-set at each DSA. No association was observed between supplement use at DSA1 and CHD mortality, for any amount of follow-up time. On the contrary, the HR of CHD mortality for SU+n3 relative to NSU at DSA2 and DSA3 was significantly below 1. With increasing follow-up time the HR attenuated, but remained statistically significant. We observed stronger associations, for all lengths of follow-up studied, between SU+n3 and CHD mortality in the later DSAs.

For peer review only - http://bmjopen.bmj.com/site/about/guidelines.xhtml

Using time-varying analysis, we observed a 26% lower hazard among n-3 PUFA supplement consumers compared to NSU (HR: 0.74, 95%CI: 0.66, 0.84). Significant interactions between follow-up time and age, sex and self-reported myocardial infarction were observed; however, inclusion of these interaction terms resulted in negligible differences in the HR or 95%CI of the main exposure. Results presented are therefore without these interaction terms in the regression equation.

Change in supplement use over time

Table 3 shows the results from analysis investigating changes in supplement use over time. There was no evidence to suggest that participants who *were* taking n-3 PUFA supplements at an earlier point in time, but who stopped its use, had a different hazard of CHD mortality compared to consistent NSU ($HR_{DSA1-DSA2}$ 1.24, 95%CI: 0.94, 1.64; $HR_{DSA2-DSA3}$ 1.20, 95%CI: 0.79, 1.81,

), therefore suggesting that there are no long term benefits of previous n-3 PUFA supplement use. Compared to consistent NSU, participants who reported n-3 PUFA supplement use at both time points, and participants who *became* a SU+n3 had a lower hazard of CHD mortality. This association was stronger in the time-varying covariates analysis where we observed a HR of 0.69 (95%CI: 0.52, 0.90) among starters of SU+n3 and a HR of 0.78 (95%CI: 0.63, 0.98) among consistent SU+n3. Associations attenuated or became non-significant as follow-up time increased.

Sensitivity analyses

Using different definitions for the endpoint (Figure 3), we observed associations of similar strength for CHD as *underlying* cause of death (HR: 0.78, 95%CI: 0.67, 0.90) and for acute fatal myocardial infarction when compared to CHD mortality mentioned *anywhere* on the death certificate. No associations were observed for first event of hospitalisation due to CHD.

Associations between n-3 PUFA supplement use and CHD mortality were similar for men and women, for overweight and non-overweight participants, for participants with and without self-reported myocardial infarction or diabetes and among smokers and non-smokers (Appendix VI). There was evidence of an interaction between supplement use

For peer review only - http://bmjopen.bmj.com/site/about/guidelines.xhtml

group and prevalent stroke (P=0.006); although the SU+n3 had similar HRs by prevalent stroke group, SU-n3 had not.

Discussion

Statement of principal findings

We observed a protective association between n-3 PUFA supplement use and CHD mortality in this general population, where the mean age was approximately 60 years at baseline (DSA1). When data from three DSA were used, lower hazards of CHD mortality were observed for SU+n3 at all three DSA, which were strongest when the follow-up time was short; however, this analysis lacked statistical power. Using a time-varying covariates analysis, we observed a 26% lower hazard of CHD mortality among SU+n3 compared to NSU.

Strengths and weaknesses of the study

A strength is the selection of participants at baseline from a general population as opposed to fish oil trials which have consisted of high risk populations [11]. Measures of covariates and supplement use were available from three time points, which enabled mitigation of potential bias due to changes in supplement use over time. We could adjust for several variables representing health behaviours, as well as co-morbidities, to account for potential confounding. The DSA had short recall periods of one week; however, we have previously shown that measures obtained from three instruments at baseline (DSA1), with recall times varying from 1 year to 1 week, had good agreement [40]. Supplement use in general is associated with health behaviours [41], so unmeasured and residual confounding could account for findings. However, heterogeneity across supplement users exists, we therefore separated participants into SU+n3 and SU-n3 and carefully assessed potential confounders at all DSA. A further strength was our use of multiple imputation to handle missing data (Appendix VII).

The EPIC-Norfolk study has limitations. The baseline response rate was 39%; however, participants were found to be representative of the Health Survey for England population regarding anthropometry, blood pressure and blood lipids [30]. There was loss to follow-up; however, outcomes were ascertained for those who no longer actively participated. Since the exact dates of supplement use were not known as were the exact dates of self-reported

For peer review only - http://bmjopen.bmj.com/site/about/guidelines.xhtml

myocardial infarction, stroke or diabetes, we are unable to say which happened first and therefore the chronological ordering is unknown. Healthier individuals might have been more likely to continue active participation; however, we adjusted for a number of factors to mitigate potential bias arising due to this. The time intervals between DSA were relatively long, particularly between DSA1 and DSA2; therefore, misclassification of the exposure is likely to have occurred. Data on fish consumption (and hence n-3 PUFA intake from food) from 7dDD could only be estimated at baseline (Appendix V). The SU+n3 and SU-n3 groups included individuals taking different numbers and types of supplements. Among the SU-n3 and SU+n3, 34% and 44% respectively consumed multiple supplements at DSA1; however, we considered the observed association between the SU+n3 and CHD mortality unlikely to be due to other supplements being used or the additional vitamins found in n-3 PUFA containing supplements, since these typically contain a 100% of the recommended nutrient intake and such doses have not been associated with cardiovascular mortality [42,43]. Nevertheless, residual confounding due to other dietary differences between SU+n3, SU-n3 and NSU, particularly those occurring over time, such as differences in fruit, vegetables, and meat, cannot be excluded. Lastly, the analyses required interpretation of many models and will have increased the role of chance findings; however, the significant findings observed may be explained by biological mechanisms and the results were specific to SU+n3.

Meaning of the study: possible explanations and implications for clinicians and policymakers

The associations observed at the low dose of supplemental EPA and DHA in this cohort makes an antiarrhythmic function of n-3 PUFA a likely biological pathway [6,7]. In time-varying covariate analysis, it was observed that n-3 PUFA supplement use was associated with 26% lower hazard of CHD mortality compared to non-supplement use. A similar association has been estimated from trial and prospective cohort data [6] which concluded that 250-500 mg/d of n-3 PUFA lowered risk by 25% or more; a dose which corresponds to the intake from food and supplements of 300 mg/d observed in this cohort among SU+n3 (Table 1). We have previously reported a non-significant association between plasma n-3 PUFA and CHD incidence in a sub-cohort of the EPIC-Norfolk study [44]; however, plasma concentrations were only available at baseline and are likely to have fluctuated over time

For peer review only - http://bmjopen.bmj.com/site/about/guidelines.xhtml

considering the variability in supplement use (Appendix II). The results between the studies are consistent when only relying on supplement data from DSA1 in the current analysis (Appendix IV). The VITAL cohort study found no significant association between increasing quartiles of n-3 PUFA intake from food and supplements and CHD (P_{trend}=0.120), although a significant trend was observed for those participants free of baseline CHD (P_{trend}=0.029). When including all DSA, effect modification for prevalent myocardial infarction was not observed in EPIC-Norfolk, which might be indicative of the different motivations for CLO use in the UK *vs.* fish oil supplement use in the US [45,46]. In Europe, the highest oily fish consumption is observed in Spain and Scandinavian countries, and is approximately 2-3 fold higher than fish intake in the UK [47]. A Norwegian cohort, where 30-41% consumed two or more fish meals per week, observed no association between CLO consumption and CHD mortality [19]. CLO could therefore have exceeded the threshold for an antiarrhythmic effect in this Norwegian cohort but might supply a considerable contribution to n-3 PUFA intake in the UK. The AGES study in Iceland, where the majority of women consumed 2-4 fish portion per week (85% lean fish), did observe a lower risk of hospitalisation due to CHD when n-3 PUFA from cod liver oil was >=5.9 g/week, whereas no association was observed with fish consumption [18]. We did not observe any association with CHD hospitalisation; however, the n-3 PUFA dose in the AGES study was much higher and therefore efficacy might have been through atherosclerotic pathways. Fish oil supplements cannot contain all the nutrients that would be obtained when consuming fish, or indeed the other meal components consumed with it (*e.g.* vegetables) or the red meat replacement that took place [48,49], but N-3 PUFA supplements contain lower concentrations of contamination [50], and provide essential fatty acids without any coating and/or frying oils which might supply trans-fatty acids which are positively associated with CHD [11]. This is consistent with findings that broiled or baked fish have been associated with a lower risk of CHD mortality, whereas fried fish has not [51]. Moreover, from DSA1, it is known that n-3 PUFA supplements were consumed on a daily basis by approximately 95% of the participants [40]. Fish consumption has not been a daily dietary habit in the UK based on National Survey data in 2000/’01 and 2008/’09-2011/’12 [4,52]. Incorporation of n-3 PUFA into plasma, platelets and red blood cells has been shown to be enhanced with a daily *vs.* twice weekly regime of similar weekly supplement doses [53].

For peer review only - http://bmjopen.bmj.com/site/about/guidelines.xhtml

Unanswered questions and future research

Food composition tables which include contaminants (Appendix V), nutrient composition from supplements and foods and which distinguish between fish preparation methods, could clarify any association between n-3 PUFA sources and CHD to further inform public health dietary guidelines.

Conclusion

Recent use of n-3 PUFA supplements was associated with a lower hazard of CHD mortality in a population-based cohort with low fish consumption. This association was specific to SU+n3 and independent of history of myocardial infarction. The masking of the association between n-3 PUFA and CHD mortality by fish consumption at baseline, through either preparation methods or contaminants, might play a role in the association between n-3 PUFA and CHD mortality, which will require further investigation with time-updated measures.

For peer review only

For peer review only - http://bmjopen.bmj.com/site/about/guidelines.xhtml

References

1 Dyerberg J. Fatty acid composition of the plasma lipids in hypothyroid subjects. *Acta Med Scand* 1968;**184**:441–9.

2 Kromhout D, Bosschieter EB, de Lezenne Coulander C. The inverse relation between fish consumption and 20-year mortality from coronary heart disease. *N Engl J Med* 1985;**312**:1205–9.

3 Zheng J, Huang T, Yu Y, *et al.* Fish consumption and CHD mortality: an updated meta-analysis of seventeen cohort studies. *Public Health Nutr* 2012;**15**:725–37.

4 Henderson L, Gregory J, Swan G. The National Diet & Nutrition Survey: adults aged 19 to 64 years. Types and quantities of foods consumed. London: : Her Majesty's Stationery Office (HMSO) 2002.

5 Lentjes MAH, Mulligan AA, Welch AA, *et al.* Contribution of cod liver oil-related nutrients (vitamins A, D, E and eicosapentaenoic acid and docosahexaenoic acid) to daily nutrient intake and their associations with plasma concentrations in the EPIC-Norfolk cohort. *J Hum Nutr Diet* 2015;**28**:568–82. doi:10.1111/jhn.12271

6 Mozaffarian D, Rimm EB. Fish intake, contaminants, and human health: evaluating the risks and the benefits. *JAMA* 2006;**296**:1885–99.

7 London B, Albert C, Anderson ME, *et al.* Omega-3 Fatty Acids and Cardiac Arrhythmias: Prior Studies and Recommendations for Future Research. *Circulation* 2007;**116**:e320–35.

8 Chiuve SE, Rimm EB, Sandhu RK, *et al.* Dietary fat quality and risk of sudden cardiac death in women. *Am J Clin Nutr* 2012;**96**:498–507.

9 Hooper L, Thompson RL, Harrison R a, *et al.* Risks and benefits of omega 3 fats for mortality, cardiovascular disease, and cancer: systematic review. *BMJ* 2006;**332**:752–60.

10 Rizos EC, Ntzani EE, Bika E, *et al.* Association between omega-3 fatty acid supplementation and risk of major cardiovascular disease events: a systematic review and meta-analysis. *JAMA* 2012;**308**:1024–33.

11 Chowdhury R, Warnakula S, Kunutsor S, *et al.* Association of dietary, circulating, and supplement fatty acids with coronary risk: A systematic review and meta-analysis. *Ann Intern Med* 2014;**160**:398–406.

12 Mozaffarian D, Wu JHY. Omega-3 fatty acids and cardiovascular disease: effects on risk factors, molecular pathways, and clinical events. *J Am Coll Cardiol* 2011;**58**:2047–67.

13 Wu JHY, Mozaffarian D. ω-3 Fatty acids, atherosclerosis progression and cardiovascular outcomes in recent trials: new pieces in a complex puzzle. *Heart* 2014;**100**:530–3.

14 James MJ, Sullivan TR, Metcalf RG, *et al.* Pitfalls in the use of randomised controlled trials for fish oil studies with cardiac patients. *Br J Nutr* 2014;**112**:812–20.

For peer review only - http://bmjopen.bmj.com/site/about/guidelines.xhtml

15 Ridker PM. Fish consumption, fish oils, and cardiovascular events: still waiting for definitive evidence. *Am J Clin Nutr* 2016;:2–3.

16 Skeie G, Braaten T, Hjartåker A, *et al.* Use of dietary supplements in the European Prospective Investigation into Cancer and Nutrition calibration study. *Eur J Clin Nutr* 2009;**63**:S226–38.

17 Messerer M, Håkansson N, Wolk A, *et al.* Dietary supplement use and mortality in a cohort of Swedish men. *Br J Nutr* 2008;**99**:626–31.

18 Haraldsdottir A, Torfadottir JE, Valdimarsdottir UA, *et al.* Fish and fish-liver oil consumption in adolescence and midlife and risk of CHD in older women. *Public Health Nutr* 2016;**19**:318–25.

19 Egeland GM, Meyer HE, Selmer R, *et al.* Cod liver oil consumption, smoking, and coronary heart disease mortality: three counties, Norway. *Int J Circumpolar Health* 2001;**60**:143–9.

20 Mozaffarian D, Lemaitre RN, King IB, *et al.* Plasma phospholipid long-chain omega-3 fatty acids and total and cause-specific mortality in older adults, a cohort study. *Ann Intern Med* 2013;**158**:515–25.

21 Bell GA, Kantor ED, Lampe JW, *et al.* Intake of Long-Chain ω-3 Fatty Acids From Diet and Supplements in Relation to Mortality. *Am J Epidemiol* 2014;**179**:710–20.

22 Yokoyama M, Origasa H, Matsuzaki M, *et al.* Effects of eicosapentaenoic acid on major coronary events in hypercholesterolaemic patients (JELIS): a randomised open-label, blinded endpoint analysis. *Lancet* 2007;**369**:1090–8.

23 The Origin Trial Investigators. Basal Insulin and Cardiovascular and Other Outcomes in Dysglycemia. *N Engl J Med* 2012;**367**:319–28.

24 Risk and Prevention Study Collaborative Group, Roncaglioni MC, Tombesi M, *et al.* n-3 fatty acids in patients with multiple cardiovascular risk factors. *N Engl J Med* 2013;**368**:1800–8.

25 Burr ML. Secondary prevention of CHD in UK men: the Diet and Reinfarction Trial and its sequel. *Proc Nutr Soc* 2007;**66**:9–15.

26 GISSI-Prevenzione Investigators. Dietary supplementation with n-3 polyunsaturated fatty acids and vitamin E after myocardial infarction: results of the GISSI-Prevenzione trial. Gruppo Italiano per lo Studio della Sopravvivenza nell'Infarto miocardico. *Lancet* 1999;**354**:447–55.

27 Rauch B, Schiele R, Schneider S, *et al.* OMEGA, a Randomized, Placebo-Controlled Trial to Test the Effect of Highly Purified Omega-3 Fatty Acids on Top of Modern Guideline-Adjusted Therapy After Myocardial Infarction. *Circulation* 2010;**122**:2152–9.

28 Galan P, Kesse-Guyot E, Czernichow S, *et al.* Effects of B vitamins and omega 3 fatty acids on cardiovascular diseases: a randomised placebo controlled trial. *BMJ* 2010;**341**:c6273.

29 Manson JE, Bassuk SS, Lee I-M, *et al.* The VITamin D and OmegA-3 TriaL (VITAL):

For peer review only

For peer review only - http://bmjopen.bmj.com/site/about/guidelines.xhtml

Rationale and design of a large randomized controlled trial of vitamin D and marine omega-3 fatty acid supplements for the primary prevention of cancer and cardiovascular disease. Contemp. Clin. Trials. 2012;**33**:159–71.

30 Day N, Oakes S, Luben R, *et al.* EPIC-Norfolk: study design and characteristics of the cohort. European Prospective Investigation of Cancer. *Br J Cancer* 1999;**80 Suppl 1**:95–103.

31 Hayat SA, Luben R, Keevil VL, *et al.* Cohort profile: A prospective cohort study of objective physical and cognitive capability and visual health in an ageing population of men and women in Norfolk (EPIC-Norfolk 3). *Int J Epidemiol* 2014;**43**:1063–72.

32 European Commission. Directive 2002/46/EC of the European Parliament and of the Council of 10 June 2002 on the approximation of the laws of the Member States relating to food supplements. *Off J Eur Communities* 2002;**L183/51**.

33 Lentjes MAH, Bhaniani A, Mulligan A a, *et al.* Developing a database of vitamin and mineral supplements (ViMiS) for the Norfolk arm of the European Prospective Investigation into Cancer (EPIC-Norfolk). *Public Health Nutr* 2011;**14**:459–71.

34 Bingham SA, Welch AA, McTaggart A, *et al.* Nutritional methods in the European Prospective Investigation of Cancer in Norfolk. *Public Health Nutr* 2001;**4**:847–58.

35 Welch AA, McTaggart A, Mulligan AA, *et al.* DINER (Data Into Nutrients for Epidemiological Research) - a new data-entry program for nutritional analysis in the EPIC-Norfolk cohort and the 7-day diary method. *Public Health Nutr* 2001;**4**:1253–65.

36 Lentjes MAH, McTaggart A, Mulligan AA, *et al.* Dietary intake measurement using 7 d diet diaries in British men and women in the European Prospective Investigation into Cancer-Norfolk study: a focus on methodological issues. *Br J Nutr* 2014;**111**:516–26.

37 Holland B, Welch AA, Unwin D, *et al. McCance and Widdowson's The Composition of Foods*. Cambridge: : Royal Society of Chemistry (RSC) 1991.

38 Mulligan AA, Kuhnle GGC, Lentjes MAH, *et al.* Intakes and sources of isoflavones, lignans, enterolignans, coumestrol and soya-containing foods in the Norfolk arm of the European Prospective Investigation into Cancer and Nutrition (EPIC-Norfolk), from 7 d food diaries, using a newly updated database. *Public Health Nutr* 2013;**16**:1454–62.

39 Welch AAA, Shakya-Shrestha S, Lentjes MAH, *et al.* Dietary intake and status of n-3 polyunsaturated fatty acids in a population of fish-eating and non-fish-eating meat-eaters, vegetarians, and vegans and the product-precursor ratio [corrected] of α-linolenic acid to long-chain n-3 polyunsaturated fatty ac. *Am J Clin Nutr* 2010;**92**:1040–51.

40 Lentjes MAH, Welch AA, Luben RN, *et al.* Differences in dietary supplement use and secular and seasonal trends assessed using three different instruments in the EPIC-Norfolk population study. *J Diet Suppl* 2013;**10**:142–51.

41 Lentjes MAH, Welch AA, Mulligan AA, *et al.* Cod liver oil supplement consumption and health: cross-sectional results from the EPIC-Norfolk cohort study. *Nutrients* 2014;**6**:4320–37.

For peer review only

For peer review only - http://bmjopen.bmj.com/site/about/guidelines.xhtml

42 Macpherson H, Pipingas A, Pase MP. Multivitamin-multimineral supplementation and mortality: a meta-analysis of randomized controlled trials. *Am J Clin Nutr* 2013;**97**:437–44.

43 Myung S-K, Ju W, Cho B, *et al.* Efficacy of vitamin and antioxidant supplements in prevention of cardiovascular disease: systematic review and meta-analysis of randomised controlled trials. *BMJ* 2013;**346**:f10.

44 Khaw K-T, Friesen MD, Riboli E, *et al.* Plasma phospholipid fatty acid concentration and incident coronary heart disease in men and women: the EPIC-Norfolk prospective study. *PLoS Med* 2012;**9**:e1001255.

45 Kim HJ, Giovannucci E, Rosner B, *et al.* Longitudinal and secular trends in dietary supplement use: Nurses' health study and health professionals follow-up study, 1986-2006. *J Acad Nutr Diet* 2014;**114**:436–43.

46 Rajakumar K. Vitamin D, cod-liver oil, sunlight, and rickets: a historical perspective. *Pediatrics* 2003;**112**:e132-5.

47 Welch A, Lund E, Amiano P, *et al.* Variability of fish consumption within the 10 European countries participating in the European Investigation into Cancer and Nutrition (EPIC) study. *Public Health Nutr* 2002;**5**:1273–85.

48 Kiefte-de Jong JC, Chowdhury R, Franco OH. Fish intake or omega-3 fatty acids: greater than the sum of all parts? *Eur J Epidemiol* 2012;**27**:891–4.

49 He K. Fish, Long-Chain Omega-3 Polyunsaturated Fatty Acids and Prevention of Cardiovascular Disease-Eat Fish or Take Fish Oil Supplement? *Prog Cardiovasc Dis* 2009;**52**:95–114.

50 Bourdon JA, Bazinet TM, Arnason TT, *et al.* Polychlorinated biphenyls (PCBs) contamination and aryl hydrocarbon receptor (AhR) agonist activity of Omega-3 polyunsaturated fatty acid supplements: implications for daily intake of dioxins and PCBs. *Food Chem Toxicol* 2010;**48**:3093–7.

51 Mozaffarian D, Lemaitre RN, Kuller LH, *et al.* Cardiac benefits of fish consumption may depend on the type of fish meal consumed: The cardiovascular health study. *Circulation* 2003;**107**:1372–7.

52 Bates B, Lennox A, Prentice A, *et al.*, editors. *National Diet and Nutrition Survey Results from Years 1 , 2 , 3 and 4 (combined) of the Rolling Programme (2008/2009-2011/2012)*. London: : Public Health England 2014.

53 Browning LM, Walker CG, Mander AP, *et al.* Compared with daily, weekly n-3 PUFA intake affects the incorporation of eicosapentaenoic acid and docosahexaenoic acid into platelets and mononuclear cells in humans. *J Nutr* 2014;**144**:667–72.

54 Holland B, Unwin I, Buss D. *Cereals and cereal products. The third supplement to McCance & Widdowson's The Composition of Foods (4th Edition)*. Cambridge: : RSC/MAFF 1988.

55 Holland B, Unwin I, Buss D. *Milk products and eggs. The fourth supplement to McCance & Widdowson's The Composition of Foods (4th Edition)*. Cambridge: : RSC/MAFF 1989.

For peer review only

For peer review only - http://bmjopen.bmj.com/site/about/guidelines.xhtml

56 Holland B, Unwin I, Buss D. *Fruit and nuts. The first supplement to McCance & Widdowson's The Composition of Foods (5th Edition)*. Cambridge: : RSC/MAFF 1992.

57 Holland B, Unwin I, Buss D. *Vegetables, herbs and spices. The fifth supplement to McCance & Widdowson's The Composition of Foods (4th Edition)*. Cambridge: : RSC/MAFF 1991.

58 Holland B, Welch A, Buss D. *Vegetable dishes. The second supplement to McCance & Widdowson's The Composition of Foods (5th Edition)*. Cambridge: : RSC/MAFF 1992.

59 Holland B, Brown J, Buss D. *Fish and fish products. The third supplement to McCance & Widdowson's The Composition of Foods (5th Edition)*. Cambridge: : RSC/MAFF 1993.

60 Chan W, Brown J, Buss D. *Miscellaneous foods. The fourth supplement to McCance & Widdowson's The Composition of Foods (5th Edition)*. Cambridge: : RSC/MAFF 1994.

61 Chan W, Brown J, Lee S, *et al. Meat, poulry and game. The fifth supplement to McCance & Widdowson's The Composition of Foods (5th Edition)*. Cambridge: : RSC/MAFF 1995.

62 Chan W, Brown J, Church S, *et al. Meat products and dishes. The sixth supplement to McCance & Widdowson's The Composition of Foods (5th Edition)*. Cambridge: : RSC/MAFF 1996.

63 Ministry of Agriculture Fisheries and Food. *Fatty aids. The seventh supplement to McCance & Widdowson's The Composition of Foods (5th Edition)*. Cambridge: : RSC/MAFF 1998.

64 Shohaimi S, Luben R, Wareham N, *et al.* Residential area deprivation predicts smoking habit independently of individual educational level and occupational social class. A cross sectional study in the Norfolk cohort of the European Investigation into Cancer (EPIC-Norfolk). *J Epidemiol Community Health* 2003;**57**:270–6.

65 Wareham NJ, Jakes RW, Rennie KL, *et al.* Validity and repeatability of a simple index derived from the short physical activity questionnaire used in the European Prospective Investigation into Cancer and Nutrition (EPIC) study. *Public Health Nutr* 2003;**6**:407–13.

66 Scientific Advisory Committee Nutrition (SACN), Committee on Toxicity. Advice on fish consumption: benefits & risks. London: : The Stationery Office (TSO) 2004.

67 Stern AH. Public health guidance on cardiovascular benefits and risks related to fish consumption. *Environ Health* 2007;**6**:31.

68 Rissanen T, Voutilainen S, Nyyssönen K, *et al.* Fish oil-derived fatty acids, docosahexaenoic acid and docosapentaenoic acid, and the risk of acute coronary events: the Kuopio ischaemic heart disease risk factor study. *Circulation* 2000;**102**:2677–9.

69 Guallar E, Sanz-Gallardo MI, van't Veer P, *et al.* Mercury, fish oils, and the risk of myocardial infarction. *N Engl J Med* 2002;**347**:1747–54.

70 Mahaffey KR, Sunderland EM, Chan HM, *et al.* Balancing the benefits of n-3 polyunsaturated fatty acids and the risks of methylmercury exposure from fish

For peer review only

For peer review only - http://bmjopen.bmj.com/site/about/guidelines.xhtml

consumption. *Nutr Rev* 2011;**69**:493–508.

71 White IR, Royston P. Imputing missing covariate values for the Cox model. *Stat Med* 2009;**28**:1982–98.

72 Nevalainen J, Kenward MG, Virtanen SM. Missing values in longitudinal dietary data: a multiple imputation approach based on a fully conditional specification. *Stat Med* 2009;**28**:3657–69.

73 White IR, Royston P, Wood AM. Multiple imputation using chained equations: Issues and guidance for practice. *Stat Med* 2011;**30**:377–99.

For peer review only

For peer review only - http://bmjopen.bmj.com/site/about/guidelines.xhtml

Tables

Table 1: Characteristics of the EPIC-Norfolk cohort at time of DSA1 (1993-1998), DSA2 (2002-2004) and DSA3 (2004-2011). Values are % (n) for categorical variables and median (IQR) for continuous variables.

	DSA1 N=22,035	DSA2 N=15,937	DSA3 N=10,120
CHD mortality events from DSA onwards (n)	1562	742	241
Total person years at risk (years)	383,444	173,239	63,535
Crude cumulative rate (per 1000 person years)	4.074	4.283	3.793
Supplement use			
NSU	61 (13444)	52 (8353)	50 (5029)
SU-n3	15 (3263)	17 (2665)	16 (1610)
SU+n3	24 (5328)	31 (4919)	34 (3481)
n-3 PUFA (g/d)*			
From food only	0.12 (0.06, 0.34)	-	-
From food and supplements combined	0.16 (0.07, 0.40)	-	-
among SU+n3 only	0.30 (0.17, 0.72)	-	-
Sex			
Men	45 (9890)	43 (6923)	44 (4418)
Women	55 (12,145)	57 (9014)	56 (5702)
Age (years)	59 (51, 67)	65 (58, 72)	69 (63, 76)
BMI (kg/m^2)	25.8 (23.7, 28.3)	26.1 (24.0, 28.7)	26.3 (24.0, 29.0)
Smoking status			
Current	11 (2395)	7 (1149)	5 (456)
Former	43 (9426)	50 (7982)	46 (4671)
Never	46 (10,214)	43 (6806)	49 (4993)
Social class			
Professional	7 (1531)	8 (1203)	8 (852)
Managerial	37 (8048)	39 (6189)	40 (4047)
Skilled non-manual	17 (3715)	16 (2585)	16 (1658)
Skilled manual	23 (5055)	22 (3483)	21 (2131)
Semi-skilled	13 (2910)	13 (2007)	12 (1163)
Non-skilled	4 (776)	3 (470)	3 (269)
Marital status			
Married	82 (18,127)	78 (12394)	76 (7708)
Not married	18 (3908)	22 (3543)	24 (2412)
Education level			
No qualification	36 (7999)	32 (5106)	28 (2851)
O-level	10 (2282)	11 (1757)	12 (1184)
A-level	41 (8955)	42 (6720)	44 (4427)
Degree	13 (2799)	15 (2354)	16 (1658)
Season of 7dDD			
Spring	26 (5817)	23 (3645)	27 (2761)
Summer	25 (5473)	21 (3295)	30 (3008)
Autumn	26 (5661)	32 (5150)	25 (2513)
Winter	23 (5084)	24 (3847)	18 (1838)

For peer review only - http://bmjopen.bmj.com/site/about/guidelines.xhtml

	DSA1 N=22,035	DSA2 N=15,937	DSA3 N=10,120
Physical activity			
Inactive	30 (6592)	36 (5809)	41 (4106)
Moderately inactive	29 (6389)	27 (4302)	28 (2792)
Moderately active	23 (5040)	20 (3151)	17 (1709)
Active	18 (4014)	17 (2675)	15 (1513)
Alcohol intake (HLQ)			
None	13 (2930)	32 (5042)	33 (3312)
>0-14 units/wk	72 (15,880)	52 (8336)	56 (5704)
>14-28 units/wk	11 (2418)	12 (1950)	9 (869)
>28 units/wk	4 (807)	4 (609)	2 (235)
Self-reported illness			
Myocardial infarction	3 (704)	5 (717)	3 (330)
Stroke	1 (304)	2 (359)	2 (164)
Diabetes	2 (510)	5 (745)	3 (338)
Energy – 7dDD (MJ/d)*	8.00 (6.71, 9.56)	-	-
Protein (en%)	15.2 (13.6, 16.9)	-	-
Fat (en%)	33.4 (29.8, 36.8)	-	-
Saturated fat (en%)	12.7 (10.9, 14.6)	-	-
Carbohydrates (en%)	47.6 (43.4, 51.5)	-	-
Alcohol (en%)	2.0 (0, 5.9)	-	-
Food intake – 7dDD (g/d)*			
Fruit	153 (84, 242)	-	-
Vegetables	142 (102, 190)	-	-
Red & processed meat	53 (32, 76)	-	-
White meat	21 (6, 37)	-	-
Oily fish	4 (0, 19)	-	-
Consumers only (54%)	17 (10, 30)	-	-
White fish	13 (0, 23)	-	-
Consumers only (66%)	19 (13, 29)	-	-
Total fish	23 (11, 40)	-	-
Consumers only (84%)	28 (17, 44)	-	-

7dDD, 7-day diet diary; HLQ, health and lifestyle questionnaire; DSA, dietary supplement assessment; BMI, body mass index; n-3 PUFA, omega-3 polyunsaturated fatty acids (sum of eicosapentaenoic acid and docosahexaenoic acid); SU, supplement user; NSU, non-supplement user.
*Data only available from DSA1, see also Appendix I; for more information on diet at DSA1 see Appendix III.

For peer review only

Table 2: The association between supplement use reported at DSA1, DSA2 and DSA3 and subsequent hazard of CHD mortality (where cause of death was mentioned anywhere on the death certificate) adjusted using model 3*. The follow-up time in the EPIC-Norfolk study was from 1993 to 2015.

		Two years of follow-up		Four years of follow-up		Follow-up time until next DSA**		Full follow-up time	
	Total N	CHD Events N	HR (95% CI)	CHD Events N	HR (95% CI)	CHD Events N	HR (95% CI)	CHD Events N	HR (95% CI)
DSA1 (1993-1998)	22,035	71		174		872		1562	
NSU	13,444	47	1.00	113	1.00	580	1.00	1012	1.00
SU-n3	3263	9	1.24 (0.60, 2.56)	20	1.17 (0.73, 1.90)	101	1.06 (0.85, 1.31)	178	0.96 (0.82, 1.13)
SU+n3	5328	15	0.95 (0.53, 1.72)	41	1.05 (0.73, 1.52)	191	0.86 (0.73, 1.02)	372	0.94 (0.83, 1.06)
DSA2 (2002-2004)	15,937	75		171		527		742	
NSU	8353	53	1.00	115	1.00	346	1.00	475	1.00
SU-n3	2665	10	1.01 (0.51, 2.02)	25	1.24 (0.79, 1.93)	61	0.97 (0.71, 1.24)	81	0.84 (0.66, 1.07)
SU+n3	4919	12	0.55 (0.29, 1.04)	31	0.64 (0.43, 0.96)	120	0.75 (0.61, 0.93)	186	0.81 (0.68, 0.97)
DSA3 (2004-2011)	10,120	59		125		241			
NSU	5029	44	1.00	82	1.00	148	1.00		
SU-n3	1610	6	0.60 (0.25, 1.42)	19	1.01 (0.61, 1.67)	35	1.00 (0.69, 1.45)		
SU+n3	3481	9	0.38 (0.18, 0.78)	24	0.52 (0.33, 0.83)	58	0.70 (0.52, 0.95)		
Time-varying***	24,330	205		470		1640			
NSU		144	1.00	310	1.00	1074	1.00		
SU-n3		25	0.93 (0.61, 1.43)	64	1.13 (0.86, 1.49)	197	0.91 (0.78, 1.06)		
SU+n3		36	0.59 (0.41, 0.85)	96	0.73 (0.58, 0.92)	369	0.74 (0.66, 0.84)		

DSA, Dietary Supplement Assessment; HR, hazard ratio; 95%CI, 95% confidence interval; NSU, non-supplement users; SU-n3, non-n-3 PUFA supplement users; SU+n3, n-3 PUFA supplement users (mainly cod liver oil).

*Using adjustment Model 3: time-point specific age, smoking, BMI, alcohol consumption, physical activity, season of questionnaire completion, marital status and self-report of myocardial infarction, stroke or diabetes; as well as: sex, social class and education measured at DSA1.

**or in case of DSA3, the censor date was the date of administrative follow-up (31 March 2015).

*** In the 'time-varying' approach, the follow-up time is divided by the dates of the respective DSA measures. Each participant is allocated to an exposure group ('NSU', 'SU-n3' or 'SU+n3'), but only for that section of the follow-up time in which they did so. If they changed supplement use (i.e. 'varied' by stopping a supplement or changing the type of supplement) the next section of the follow-up time (until the next DSA) was allocated to the exposure group they changed to. This type of analysis reduces misclassification of the exposure and any other confounders over time in a single analysis. Please note, the variables in this analysis do not explain what the associated risk is when changing from a specific supplement user category to another, this is shown in Table 3. The re-shaped dataset for time-varying analyses contains a larger number

For peer review only - http://bmjopen.bmj.com/site/about/guidelines.xhtml

of participants (N=24,330) than available at DSA1 alone since 2295 participants did not complete DSA1, but did complete DSA2 and/or DSA3; equally, some participants were excluded from DSA1 due to missing covariate data when these covariates were available at DSA2 and/or DSA3 and so the participant contributed follow-up time from DSA2 and/or DSA3 only.

For peer review only

For peer review only - http://bmjopen.bmj.com/site/about/guidelines.xhtml

Table 3: The association between change in CLO consumption and subsequent hazard of CHD mortality (where cause of death was mentioned anywhere on the death certificate). The follow-up time in the EPIC-Norfolk study was from 2002 for DSA1-DSA2 and from 2004 for DSA2-DSA3 (until 2015)

		Two years of follow-up		Four years of follow-up		Follow-up time until next DSA*		Full follow-up time	
	Total N	Events N	HR (95% CI)	Events N	HR (95% CI)	Events N	HR (95% CI)	Events N	HR (95% CI)
Change between DSA1-DSA2	14,283	71		162		477		672	
Consistent NSU	5861	42	1.00	89	1.00	240	1.00	335	1.00
Were SU+n3	1297	9	1.03 (0.50, 2.12)	21	1.13 (0.70, 1.82)	64	1.24 (0.94, 1.64)	81	1.12 (0.88, 1.43)
Became SU+n3	2318	6	0.58 (0.25, 1.38)	18	0.79 (0.47, 1.31)	46	0.69 (0.50, 0.94)	75	0.77 (0.60, 1.00)
Consistent SU+n3	2213	6	0.51 (0.22, 1.21)	13	0.50 (0.28, 0.90)	65	0.87 (0.66, 1.15)	96	0.88 (0.70, 1.11)
Other type of SU	2594	8	0.71 (0.33, 1.53)	21	0.90 (0.56, 1.46)	62	0.93 (0.70, 1.24)	85	0.86 (0.67, 1.09)
Change between DSA2-DSA3	9221	58		118		218			
Consistent NSU	3427	37	1.00	59	1.00	107	1.00		
Were SU+n3	932	5	0.58 (0.23, 1.49)	17	1.22 (0.71, 2.11)	29	1.20 (0.79, 1.81)		
Became SU+n3	1173	2	0.22 (0.05, 0.94)	8	0.54 (0.25, 1.12)	17	0.63 (0.38, 1.06)		
Consistent SU+n3	2037	7	0.41 (0.18, 0.93)	15	0.54 (0.31, 0.96)	37	0.75 (0.52, 1.10)		
Other type of SU	1652	7	0.57 (0.25, 1.29)	19	0.97 (0.58, 1.65)	28	0.79 (0.52, 1.21)		
*Time-varying***	15,356	129		280		695			
Consistent NSU		79	1.00	148	1.00	347	1.00		
Were SU+n3		14	0.81 (0.46, 1.44)	38	1.18 (0.82, 1.69)	93	1.25 (0.99, 1.57)		
Became SU+n3		8	0.42 (0.20, 0.88)	26	0.70 (0.46, 1.07)	63	0.69 (0.52, 0.90)		
Consistent SU+n3		13	0.46 (0.26, 0.84)	28	0.53 (0.35, 0.80)	102	0.78 (0.63, 0.98)		
Other type of SU		15	0.64 (0.37, 1.13)	40	0.92 (0.65, 1.32)	90	0.85 (0.68, 1.08)		

DSA, Dietary Supplement Assessment; HR, hazard ratio; 95%CI, 95% confidence interval; NSU, non-supplement users; SU-n3, non-n-3 PUFA supplement users; SU+n3, n-3 PUFA supplement users (mainly cod liver oil).

Using adjustment Model 3: covariates are from the latest considered time-point (*e.g.* DSA2, when analysing change between DSA1 and DSA2): age, smoking, BMI, alcohol consumption, physical activity, season of questionnaire completion, marital status and self-report of myocardial infarction, stroke or diabetes; as well as: sex, social class and education measured at DSA1.

*or in case of DSA3, the censor date was the date of administrative follow-up (31 March 2015).

For peer review only - http://bmjopen.bmj.com/site/about/guidelines.xhtml

**The re-shaped dataset contains a larger number of participants (N=15,356), since participants did not complete DSA1, but did complete DSA2 and/or DSA3; equally, some participants were excluded from DSA1 due to missing covariate data when these covariates were available at DSA2 and/or DSA3 and so the participant contributed follow-up time from DSA2 and/or DSA3 only.

For peer review only

Figures

Figure 1: Number of participants included at each DSA time point studied, with different scenarios of exclusion due to missing data.

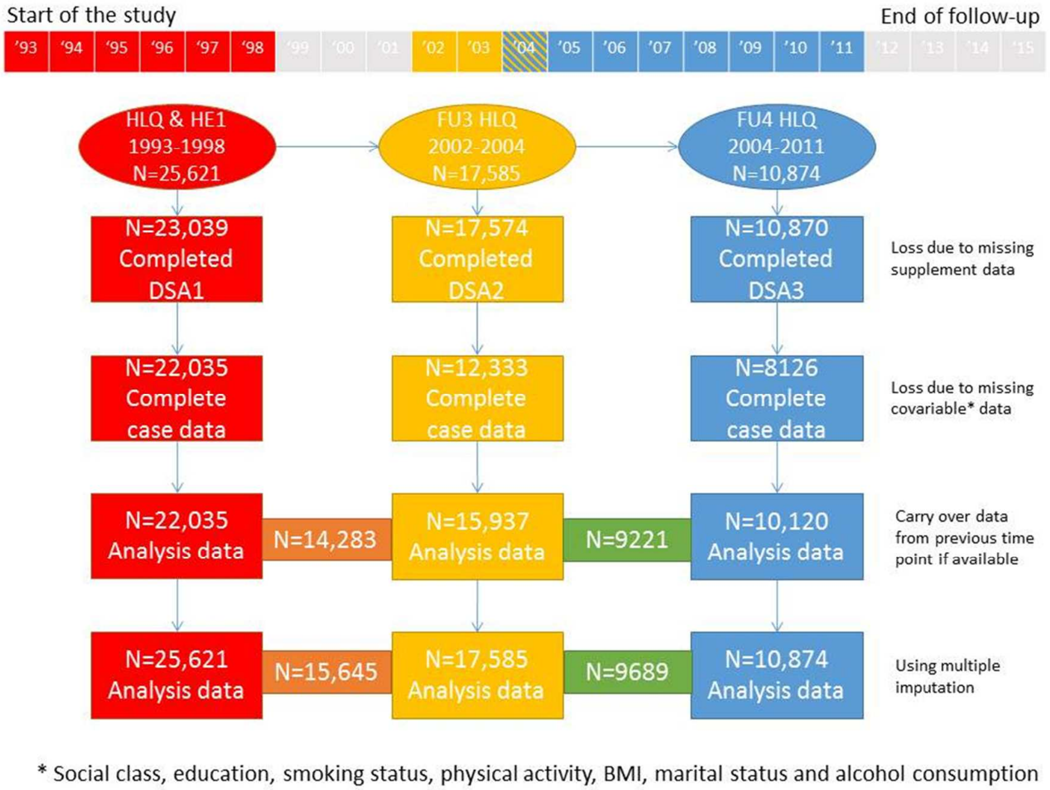

DSA, dietary supplement assessment; HLQ, health and lifestyle questionnaire; HE, health examination.

The numbers in the orange and green boxes relate to the number of participants available when the two consecutive DSA were considered in the analysis. The grey area in the timeline represents unobserved time. For more information regarding data availability see Appendix I.

For peer review only - http://bmjopen.bmj.com/site/about/guidelines.xhtml

Figure 2: Characteristics of SU-n3 vs. NSU and SU+n3 vs. NSU measured at three time points (DSA1, DSA2, DSA3) in the EPIC-Norfolk study.

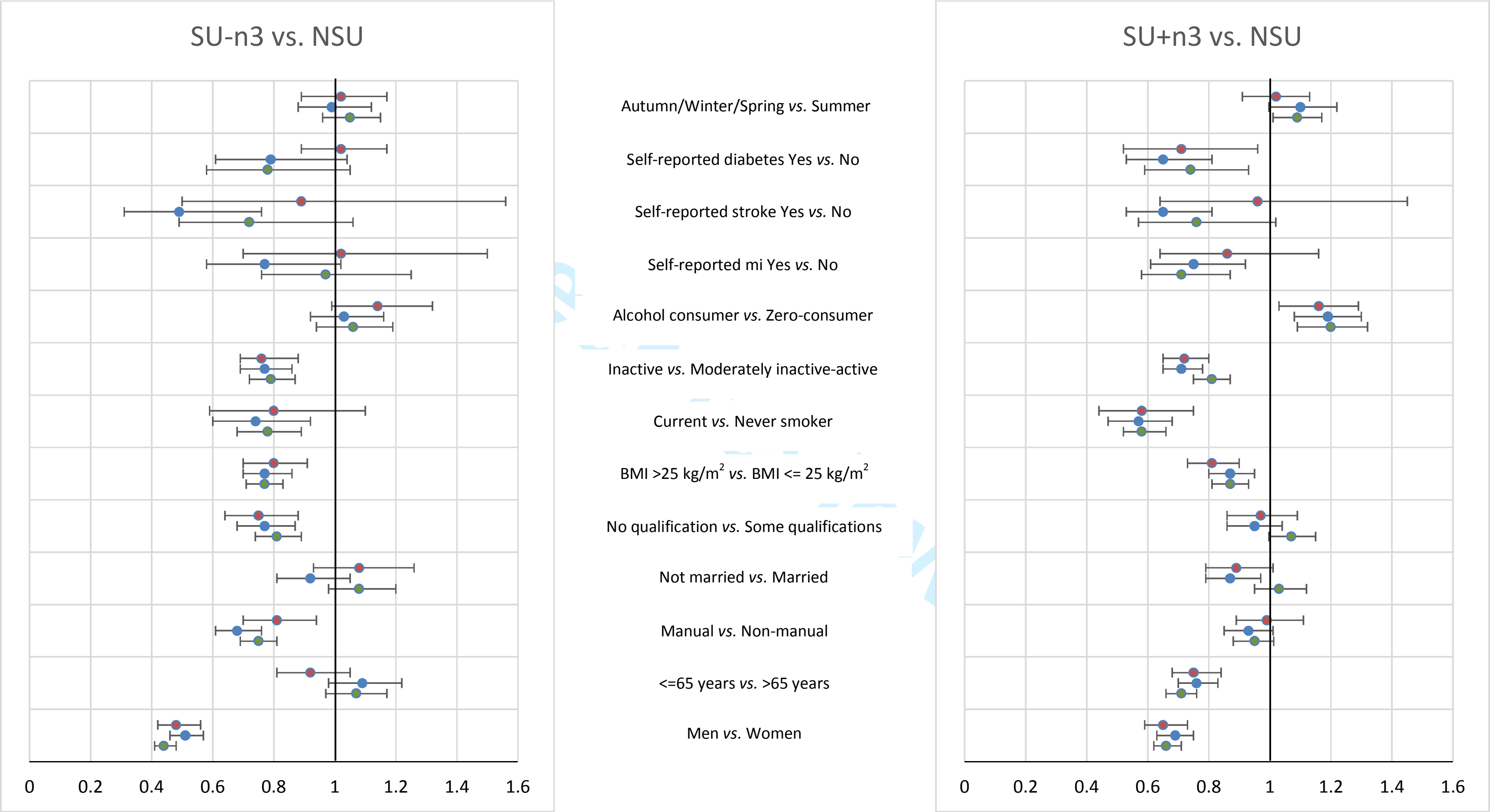

Three analyses from multinomial logistic regression at three time points: DSA1 (N=22,035), DSA2 (N=12,330), DSA3 (N=8126). The ORs are mutually adjusted for all variables shown. DSA, dietary supplement assessment; NSU, non-supplement user; SU+n3, n-3 PUFA supplement user; SU-n3; supplement user without n-3 PUFA.

Figure 3: Time-varying covariate analysis of the association between CLO consumption and hazard of CHD in the EPIC-Norfolk study (follow-up time from 1993 to 2015).

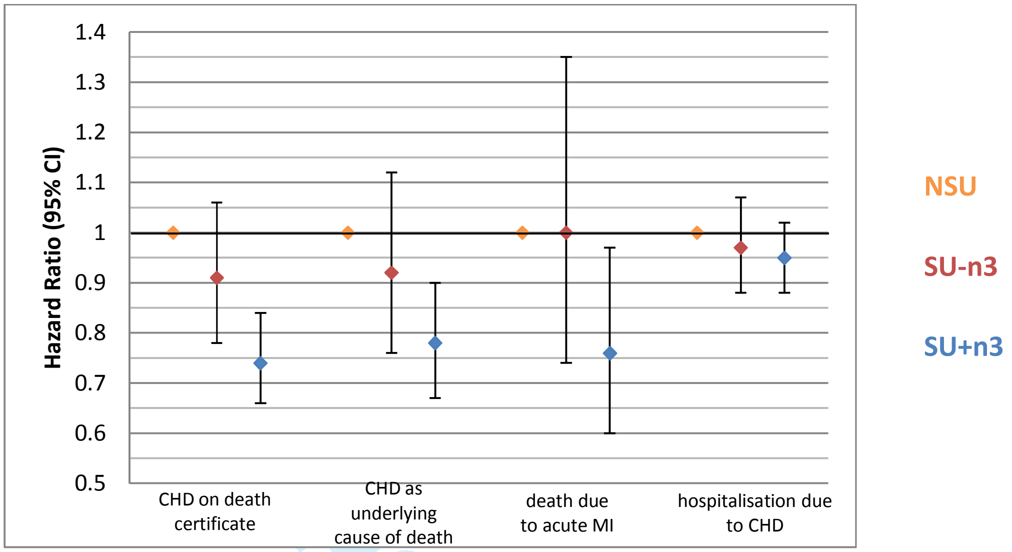

NSU, non-supplement users; SU-n3, non-N-3 PUFA supplement users; SU+n3, N-3 PUFA supplement users (mainly cod liver oil); MI, myocardial infarction.

(i) CHD mentioned *anywhere* on the death certificate (n=1640/24,330, as Table 2).
(ii) CHD mentioned as *underlying* cause of death on death certificate (n=1084/24,330).
(iii) *Acute myocardial infarction* as underlying cause of death on death certificate (n=411/24,330).
(iv) *Hospitalisation* due to CHD (n=4087/24,216).

The re-shaped dataset for mortality analysis contains a larger number of participants (N=24,330) than available at DSA1 alone and therefore more events, since participants did not complete DSA1, but did complete DSA2 and/or DSA3; equally, some participants were excluded from DSA1 due to missing covariate data whereas these covariates were available at DSA2 and/or DSA3 and so the participant contributed follow-up time from DSA2 and/or DSA3 only.

The re-shaped dataset for hospitalisation analysis contains a smaller number of participants (N=24,216) than the mortality analysis, since participants who did not complete DSA1, but completed DSA2 or DSA3 -however had a non-fatal event before DSA2 or DSA3 respectively- were excluded.

Using adjustment Model 3: time-point specific age, smoking, BMI, alcohol consumption, physical activity, season of questionnaire completion, marital status and self-report of myocardial infarction, stroke or diabetes; as well as: sex, social class and education.

For peer review only - http://bmjopen.bmj.com/site/about/guidelines.xhtml

Appendices

Appendix I: Methods - extended version. 34
Appendix II: Consistency of and change in supplement use from data collected at three time points in EPIC-Norfolk (1993-2011). 45
Appendix III: Characteristics of participants by type of supplement used as measured at DSA1 (1993-1998). 46
Appendix IV: Hazard ratios for CHD mortality by supplement user subgroups assessed at DSA1 only. 48
Appendix V: Fish or Supplement? 49
Appendix VI: Interactions using time-varying covariate analysis for the association between CLO consumption and risk of CHD mortality (1993-2015). 55
Appendix VII: Multiple Imputation 57

For peer review only

Abbreviations:

DSA: Dietary Supplement Assessment

HE: Health Examination

HLQ: Health and Lifestyle Questionnaire

NSU: Non-Supplement User

SU: Supplement user

EPA: eicosapentaenoic acid

DHA: docosahexaenoic acids

n-3 PUFA: omega-3 poly unsaturated fatty acids (EPA+DHA)

7dDD: 7-day Diet Diary

For peer review only - http://bmjopen.bmj.com/site/about/guidelines.xhtml

Appendix I: Methods - extended version

This Appendix may be read as a replacement of the method section in the manuscript.

Study design

Recruitment for the Norfolk-based European Prospective Investigation into Cancer and Nutrition (EPIC-Norfolk) started in 1993 and was completed in 1998 [30,31]. Thirty-five general practices in the Norfolk area of East-Anglia (UK) took part in the study; 77,630 registered patients were approached, 30,445 consented. Exposure assessment has been repeated over the course of the study; participants completed general Health and Lifestyle Questionnaires (HLQ) and attended three completed rounds of health examinations (HE) up to 2011 (Figure 1, Appendix I-Table 1). Data on health outcomes and mortality were collected passively. Ethical approval for the study was given by the Norwich District Health Authority Ethics Committee. Participants gave written informed consent.

For peer review only

Appendix I-Table 1: Availability of variables from questionnaires and health examinations in the EPIC-Norfolk study (1993-2011) for all those who consented.

	HLQ (1993-1998)	HE1 (1993-1998) *DSA1*	HE2 (1998-2000)	FU3 (2002-2004) *DSA2*	FU4 (2004-2011) *DSA3*	HE3 (2004-2011)
Approached for questionnaire (n)	77,630			25,846	18,380	
Completed questionnaire (n)	30,411			17,585	10,874	
Invited for HE (n)		30,445	19,560			10,826
Attended HE (n)		25,639	15,786			8,623
Retention (% relative to attenders HE1)			62	69	42	34
Measured anthropometry obtained at HE						
Height-Weight-BMI (n)		25,582	15,758			8603
Self-reported socio-economic & lifestyle factors from questionnaires						
Social Class (n)	29,673					
Education level (n)	30,411					
Marital status (n)	30,247			17,447	10,770	
Self-reported illnesses (n)	30,409			17,580	***10,874	
Smoking (n)	30,158			17,494	10,770	
Physical activity (n)	30,410			17,585	10,873	
Alcohol consumption (n)	30,051			17,429	10,592	
Diet measured using a 7dDD* (n)		25,525				
*Self-reported supplement use from questionnaires***						
Completed (n)		23,039		17,574	10,870	
Type of instrument		7dDD		HLQ	HLQ	
Recall time		1 week		1 week	1 week	
Median time since previous DSA (IQR), years		-		7.6 (7.1, 8.1)	5.6 (4.0, 6.5)	

HE, health examination; FU, follow-up; DSA, dietary supplement assessment; 7dDD, 7-day Diet Diary; HLQ, Health and Lifestyle Questionnaire; IQR, Interquartile Range.

*7-day Diet Diaries were repeated at HE2 and HE3, but only a small, non-randomly selected proportion of these data have been entered and are available for statistical analysis. Equally, Food Frequency Questionnaires have been administered at all HE; however, only the data from the FFQ at HE1 and HE2 have been entered and are available for analysis; moreover, the supplement data from the FFQ have not been entered using the ViMiS system [33] and were hence not available or compatible with the supplement data used in the described analysis. For these reasons, only baseline dietary data, using the 7dDD, have been used in this analysis.

For peer review only

For peer review only - http://bmjopen.bmj.com/site/about/guidelines.xhtml

** Dietary supplement use was assessed at *five* time points (two additional HLQs were situated in between DSA1 and DSA2). These additional DSA were hybrid questions, combining supplement use with medication use, the answers to which did not follow cohort trends or indeed trends in national survey data. The first additional DSA had the extra complication that the supplement/medication question was only introduced after some time, making the data only available on a subset of the participants. For these reasons, only three dietary supplement assessments (referred to as 'DSA', and for ease, numbered consecutively) were useable for analysis all with a recall time of one week (see also Figures 1, 2 and 3 in this Appendix).

***Data are obtained from a combination of self-reported illnesses at the time of the HLQ and FU3 since this question was not asked for at FU4. Missing values were assumed to mean 'no'.

For peer review only

Assessment of n-3 PUFA supplement use

Dietary supplements were defined according to the EU directive 2002/46/EC [32]: "Food stuffs the purpose of which is to supplement the normal diet and which are concentrated sources of nutrients or other substances with a nutritional or physiological effect, alone or in combination, marketed in dose form… ." Prescribed medication containing minerals and/or vitamins (e.g. ferrous sulphate) were not considered supplements. The sum of EPA and DHA from supplements were the main exposure, referred to as n-3 PUFA. We used data from three dietary supplement assessments (DSA), named DSA1, DSA2, DSA3. The three DSA are from different postal data collection methods with a slightly different phrasing of the supplement use question, but all covered a one week recall and shared basic question elements such as naming of vitamins or food supplements and a request for the supplement composition (Figures 1-3 in this Appendix).

Appendix I-Figure 1: DSA1

1st version, used from 1994 through out 1996

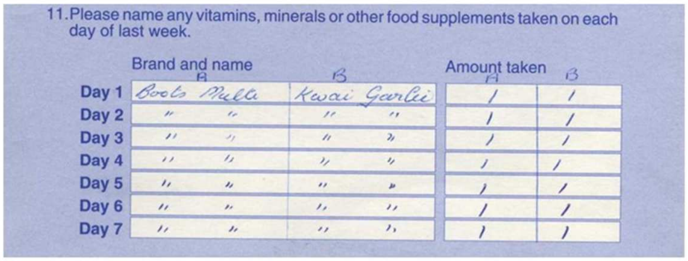

11. Please name any vitamins, minerals or other food supplements taken on each day of last week.

	Brand and name A	Brand and name B	Amount taken A	Amount taken B
Day 1	Boots Multi	Kwai Garlic	/	/
Day 2	" "	" "	/	/
Day 3	" "	" "	/	/
Day 4	" "	" "	/	/
Day 5	" "	" "	/	/
Day 6	" "	" "	/	/
Day 7	" "	" "	/	/

2nd version, used between 1996 and 1998.

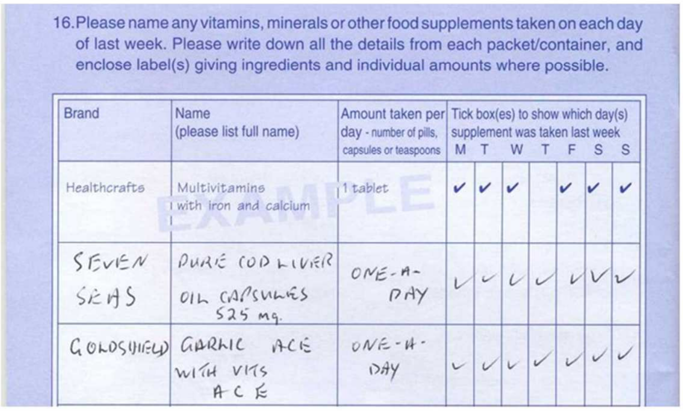

16. Please name any vitamins, minerals or other food supplements taken on each day of last week. Please write down all the details from each packet/container, and enclose label(s) giving ingredients and individual amounts where possible.

Brand	Name (please list full name)	Amount taken per day - number of pills, capsules or teaspoons	M	T	W	T	F	S	S
Healthcrafts	Multivitamins with iron and calcium	1 tablet	✔	✔	✔		✔	✔	✔
SEVEN SEAS	PURE COD LIVER OIL CAPSULES 525 mg.	ONE-A-DAY	✔	✔	✔	✔	✔	✔	✔
GOLDSHIELD	GARLIC ACE WITH VITS A C E	ONE-A-DAY	✔	✔	✔	✔	✔	✔	✔

(Column heading over M–S: Tick box(es) to show which day(s) supplement was taken last week. The Healthcrafts row is marked EXAMPLE.)

For peer review only - http://bmjopen.bmj.com/site/about/guidelines.xhtml

Appendix I-Figure 2: DSA2

MEDICATIONS AND SUPPLEMENTS *(continued)*

86 In **the last week**, have you taken any vitamins, food supplements etc. Yes ☐ No ☐ Don't know ☐

(Please name any vitamins, minerals or other food supplements taken on each day of last week. Please write down all the details from each packet/container.)

Brand	Name (Please list full name)	Amount taken per day – number of pills, capsules or teaspoons.	Tick (✓) box(es) to show which day(s) supplement was taken last week. M	T	W	T	F	S	S

Appendix I-Figure 3: DSA3

63. In the last week, have you taken any vitamins, food supplements etc? Yes ☐ No ☐ Don't know ☐

(Please name any vitamins, minerals or other food supplements taken on each day of last week **as in the example below**.)

Brand	Name (Please list **full** name)	Amount taken per day – number of pills, capsules or teaspoons.	Tick (✓) box(es) to show which day(s) supplement was taken last week. M	T	W	T	F	S	S
Healthcrafts	Multivitamins with iron and calcium	1 tablet	✓		✓		✓	✓	

Example

For peer review only - http://bmjopen.bmj.com/site/about/guidelines.xhtml

At each DSA, participants were grouped by the type of supplement they consumed:

- non-supplement users (NSU);
- non n-3 PUFA supplement users (SU-n3): participants who consumed one or more supplements that did not contain n-3 PUFA;
- n-3 PUFA supplement users (SU+n3): participants who used n-3 PUFA supplements, either singly or in combination with other non n-3 PUFA supplements. The supplements included in the n-3 PUFA group contained cod liver oil (92%) and/or other types of fish oil (19%) such as halibut liver oil; 81% of the SU+n3 used a supplement which contained only cod liver oil.

Participants were also grouped into five categories identifying change in n-3 PUFA supplement use between two consecutive DSA (*i.e.* DSA1-DSA2; DSA2-DSA3):

Appendix I-Figure 4: Supplement change categories (see also Appendix II).

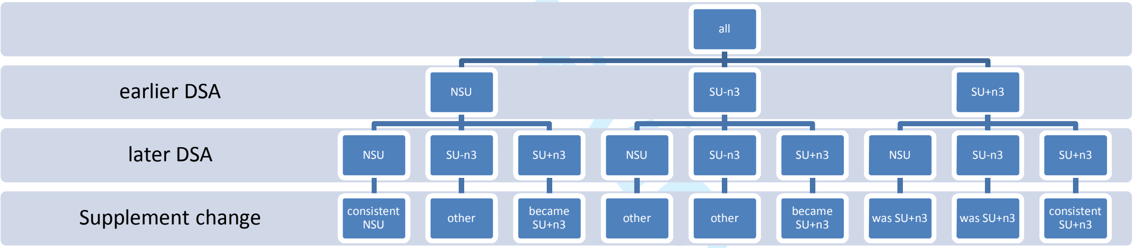

The Vitamin and Mineral Supplement (ViMiS) system to calculate the nutrient quantity from supplements assessed at DSA1, has been described in detail elsewhere [33]. In brief, the supplement described by the participant was coded with regards to supplement name, brand name, strength/unit, type and frequency of consumption. These data were matched against data collected from manufacturers' labels of 2162 dietary supplements. Where no match could be found, one of the 600 best-fitting generic supplement nutrient compositions was assigned. Supplement quantification of nutrients was made compatible with nutrient quantification in food composition tables. Missing values of two of the n-3 PUFAs, EPA and DHA, in supplements were replaced with cod liver oil/fish-specific, capsule/liquid-specific as well as strength-specific nutrient values. Average daily nutrient composition from

For peer review only

For peer review only - http://bmjopen.bmj.com/site/about/guidelines.xhtml

supplements was calculated and added to the average daily food intake to obtain average total nutrient intake. N-3 PUFA intake were compared with n-3 PUFA biomarkers (plasma EPA and DHA) available on a subset (n=6467) of EPIC-Norfolk participants; we observed a 28% higher concentration of plasma n-3 PUFA among SU+n3 vs. NSU (median [interquartile range, IQR] of plasma n-3 PUFA concentrations were: NSU 268 [206-354] umol/L, SU-n3 281 [223-382] umol/L, SU+n3 343 [256-453] umol/L) [5].

Dietary covariates

The analyses adjust for potential dietary confounders measured at DSA1. Diet was assessed using a 7-day diet diary (7dDD) between 1993-1998 [34]. Participants were asked to record food and drinks consumed in an A5-size booklet where the days were pre-structured into seven meal occasions and a checklist. Portion sizes could be described using colour photographs, household measures, or food packaging. A nurse completed the first day as a 24-hour diet recall in order to indicate the level of detail necessary. Data were entered by trained staff into DINER [35] and checked and calculated by nutritionists using DINERMO [36]. The underlying food composition tables were derived from McCance & Widdowson's 5th edition [37], including the supplementary data [54–63]. Missing values for fatty acids were further completed using a recipe calculation system [38,39]. The baseline 7dDD provided data on average daily energy intake (MJ/d), proportion of energy provided by macronutrients (%En), the sum of EPA and DHA intake (g/d) referred to as 'n-3 PUFA' and consumption of the disaggregated amounts of fruit, vegetable, red meat, processed meat, white meat, and white and oily fish consumption (g/d) [36].

Assessment of other covariates

The analyses at all DSA adjust for a number of non-dietary variables which are potential confounders. Social class was measured through occupational status, which was classified according to the registrar general's occupational classification scheme [64] (professional, managerial, skilled non-manual, skilled manual, semi-skilled, non-skilled). Highest education level achieved was divided in four categories (no qualification, O-level, A-level, degree). Both variables were derived from HLQ1 (coinciding with DSA1) and are time-fixed. The remaining covariates are time-dependent and refer to measures obtained at time points corresponding to the DSA. We identified participants at higher risk of CHD using responses

For peer review only - http://bmjopen.bmj.com/site/about/guidelines.xhtml

to the questions “Has the doctor ever told you that you have [myocardial infarction/diabetes/stroke]?”. Participants were classified as a never smoker, former smoker or current smoker based on responses to the questions “Have you ever smoked as much as one cigarette a day for as long as a year?” and “Do you smoke cigarettes now?”. Alcohol consumption, was classified as none, >0-14 units/wk, >14-28 units/wk, >28 units/wk using the sum of the number of units consumed per week of beer, spirits, wine and fortified wine. Marital status was re-categorised into married (married/living as married) or non-married (widowed, divorced, separated, single). Participants were classified as being active, moderately active, moderately inactive or inactive using a validated 4-point scale taking into account both occupational and leisure time activity [65]. At the HE, BMI (kg/m^2) was calculated from height and weight measured by a trained nurse. The collection of non-dietary variables did not always coincide exactly with the DSA: see Table 1 (in this Appendix) for an overview of available variables at respective time points.

Participant selection, case ascertainment and outcomes studied

Participants were eligible for analyses if they provided data on supplement use at any of the three time points (DSA1, DSA2, DSA3) and attended the corresponding HE (Figure 1). Four thousand and thirty participants were not included at any time point studied. The participant’s National Health Services number (a unique national patient identifier) was linked to the data from the Office of National Statistics to obtain vital status and causes of death. A similar procedure was followed for causes of hospital admissions registered by the East Norfolk Primary Health Care Trust which records for its residents the admissions in England and Wales. The main endpoint studied was Coronary Heart Disease (CHD) mortality mentioned anywhere on the death certificate, identified with ICD9 410-414 or ICD10 I20-I25. In sensitivity analyses, we investigated the use of different definitions for the CHD endpoint: (i) CHD mortality mentioned as underlying cause of death, (ii) death due to acute myocardial infarction (ICD-codes 410 or I21), (iii) first recorded hospitalisation due to CHD. We hypothesised to observe associations between n-3 PUFA containing supplements and CHD death; however, considering the different etiological pathways of atherosclerosis we did not expect to find an association at this low ingested dose of n-3 PUFA [5] and hospitalisation (non-fatal) CHD [6].

For peer review only - http://bmjopen.bmj.com/site/about/guidelines.xhtml

Statistical analysis

A description of the cohort at DSA1, DSA2 and DSA3 was made for supplement use, socio-demographic characteristics, self-reported illnesses, anthropometry, and nutrient and food intake. For categorical variables, counts and percentages were used; for continuous variables, median and IQR were used.

To study potential changes in supplement user characteristics over time, a multinomial logistic regression analysis was performed at each DSA with supplement use group as the outcome variables and the variables noted above as explanatory variables.

The main analyses are based on Cox proportional hazards models, with follow-up time as the underlying time variable. Individuals who died from causes other than the event of interest were censored at their date of death and individuals who did not die from any cause were censored at the end of follow-up (31st March 2015). Follow-up was therefore for up to 22 years (1993 to 2015).

The Cox regression modelling began by investigating associations between supplement use at DSA1 (SU+n3 *vs.* NSU and SU-n3 *vs.* NSU) and CHD mortality, with adjustment for covariates measured at DSA1. A series of *cumulative* adjustment models were used: sex and age-adjusted estimates (model 1); including smoking, BMI (kg/m^2), physical activity, alcohol intake, social class, education and season in which the questionnaire was completed (model 2); including self-reported myocardial infarction, stroke or diabetes (model 3); including energy intake (MJ/d) and disaggregated fruit (g/d), vegetable (g/d), red meat & processed meat (g/d), and white meat (g/d) (model 4); including white fish (g/d) and oily fish (g/d) (model 5). Models 1-5 were also fitted using quintiles of n-3 PUFA intake from food and supplements at DSA1 as the main exposure in place of supplement groupings. Note that Model 5 includes adjustment for fish consumption, which is a source of n-3 PUFA and therefore contributes to n-3 PUFA intake from food and supplements. Model 5 therefore addresses the question of whether higher n-3 PUFA intake from food and supplements is associated with CHD mortality among individuals consuming equal quantities of fish.

The above analyses relate to variables measured at DSA1 to the hazard of CHD mortality over a long period of follow-up (up to 22 years). The results are therefore likely to be

For peer review only - http://bmjopen.bmj.com/site/about/guidelines.xhtml

affected by changes in supplement use over time and the short-term effect of n-3 PUFA on arrhythmia [6]. We therefore repeated the analysis using time-updated measures of supplement use and covariates from DSA2 and DSA3 to reduce misclassification. We performed this analysis in two ways. First, in separate analyses using DSA1, DSA2 and DSA3 as the time origins (the first of these is the same as the analysis described above; however, all DSA used adjustment model 3 since dietary variables were not available for DSA2 and DSA3). Secondly, we performed a single analysis using the most up-to-date exposure and covariate measures for each individual at each time they were at risk, *i.e.* time-varying covariates modelling. These models used the full length of follow-up. Additionally, we restricted the follow-up time at each DSA to two and four years of follow-up as well as until the start of the next DSA (at which time participants were censored). We did this to acknowledge unobserved changes in individuals' supplement use over time which could result in biased associations over time. We equally applied the strategy described in this paragraph using the *change* between supplement group categories *between* DSA (DSA1-DSA2 and DSA2-DSA3) as the main exposure; the latter analyses were therefore restricted to participants having completed two consecutive DSA (Figure 1).

Using time-varying covariates modelling and maximum follow-up time, we explored potential effect modification using the main endpoint (CHD mortality mentioned anywhere) by including interaction terms of supplement use with sex, age, self-reported illnesses, smoking or BMI. The likelihood ratio tests were used to compare the models with and without the respective interaction term.

In sensitivity analyses, we investigated the use of different definitions for the CHD endpoint (as listed earlier) using time-varying covariates modelling and adjustment model 3.

There were missing data in the main exposure and in the covariates. For the descriptive statistics, multinomial logistic analyses and the main Cox regression analyses the missing data were handled as follows. At DSA1, participants with missing data on supplement use and covariables were excluded; at DSA2 and DSA3, missing data on time-dependent covariates, but not supplement use, were carried over from previous DSA where available, *e.g.* missing smoking status at DSA3 would take the value at DSA2. Since DSA2 was a postal data collection only, BMI was taken from the nearest health examination (HE2). Self-

For peer review only - http://bmjopen.bmj.com/site/about/guidelines.xhtml

reported prevalent illnesses were not asked for at the time of DSA3, hence data from DSA1 and DSA2 were carried over. The use of the 'last observation carried forward' to handle missing data in time-dependent covariates at DSA2 and DSA3 resulted in some data still being missing at these time points and these individuals were excluded from the main analyses. In the Cox regression analyses we also used multiple imputation as an alternative method for dealing with missing data; details of the approach taken are provided in Appendix VII.

The proportional hazards assumption was tested based on the time-varying covariates model. Significant interactions between time and age, sex and self-reported myocardial infarction were observed; however, inclusion of these variables with their time interaction terms resulted in negligible differences in the HR or 95%CI of the main exposure (supplement use). Results presented are therefore without these interaction terms in the regression equation.

Data were analysed using Stata v14.

Patient involvement

During the study, EPIC-Norfolk participants have received newsletters with summaries of publications and announcements regarding study planning and progression. Researchers have given talks in community groups about the study in general and its findings. Public events have included representation of the EPIC-Norfolk study at the Cambridge Science Festival, of which the resources have been made available to local schools: http://www.sciencefestival.cam.ac.uk/resources. In 2009, EPAP was established (EPIC-Norfolk Participant Advisory Panel). The contributions of this group of participants has varied from providing potential research topics and assessing the potential burden of research activities to clarifying (research) materials to be send to the cohort. This meant that questionnaires, newsletters and participant information sheets could be improved before being sent to our full cohort or to the ethics committee. Summaries of these meetings may be found on: http://www.srl.cam.ac.uk/epic/participant_panel.html.

For peer review only - http://bmjopen.bmj.com/site/about/guidelines.xhtml

Appendix II: Consistency of and change in supplement use from data collected at three time points in EPIC-Norfolk (1993-2011).

Below diagram includes all participants who consented and completed the dietary supplement assessments (DSA) from the three included time points (N=8252). The first subdivide in the diagram is the distribution of supplement use as reported at DSA1; the second at DSA2; the third at DSA3. The percentages represent the number relative to the total, indicating consistency over time; the top 3 'pathways' are marked in red.

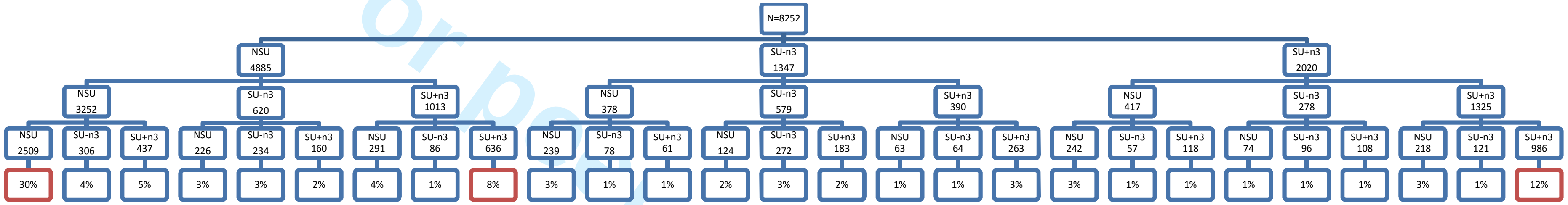

For peer review only - http://bmjopen.bmj.com/site/about/guidelines.xhtml

Appendix III: Characteristics of participants by type of supplement used as measured at DSA1 (1993-1998).

	Full cohort N=22,035	NSU N=13444	SU-n3 N=3263	SU+n3 N=5328	P[a]
CHD mortality	7.1 (1562)	7.5 (1012)	5.5 (178)	7.0 (372)	
Sex					<0.001
Men	45 (9890)	50	31	40	
Women	55 (12,145)	50	70	60	
Age [b]					
=<50 years	20 (4496)	22	25	13	<0.001
>50-60 years	32 (6984)	32	33	31	
>60-70 years	32 (7058)	31	29	37	
>70 years	16 (3497)	16	13	18	
BMI [c]					
=<20 kg/m^2	2 (491)	2	4	2	<0.001
>20-25 kg/m^2	38 (8369)	36	44	40	
>25-30 kg/m^2	45 (9979)	46	41	46	
>30 kg/m^2	15 (3196)	16	11	13	
Smoking status					<0.001
Current	11 (2395)	13	10	7	
Former	43 (9426)	43	40	45	
Never	46 (10,214)	45	51	48	
Social class					<0.001
Professional	7 (1531)	7	8	6	
Managerial	37 (8048)	36	42	36	
Skilled non-manual	17 (3715)	16	18	19	
Skilled manual	23 (5055)	24	18	23	
Semi-skilled	13 (2910)	14	11	13	
Non-skilled	4 (776)	4	3	3	
Marital status					<0.001
Married	82 (18,127)	83	80	81	
Not married	18 (3908)	17	20	19	
Education level					<0.001
No qualification	36 (7999)	37	31	39	
O-level	10 (2282)	10	12	10	
A-level	41 (8955)	40	43	41	
Degree	13 (2799)	13	15	11	
Season of 7dDD					0.022
Spring	26 (5817)	27	25	27	
Summer	25 (5473)	25	25	24	
Autumn	26 (5661)	26	26	25	
Winter	23 (5084)	22	24	24	
Physical activity					<0.001
Inactive	30 (6592)	32	26	28	
Moderately inactive	29 (6389)	28	31	31	
Moderately active	23 (5040)	23	25	23	
Active	18 (4014)	18	19	19	

For peer review only

	Full cohort N=22,035	NSU N=13444	SU-n3 N=3263	SU+n3 N=5328	P[a]
Alcohol intake (HLQ)					<0.001
None	13 (2930)	14	13	13	
>0-14 units/wk	72 (15,880)	71	74	74	
>14-28 units/wk	11 (2418)	11	10	11	
>28 units/wk	4 (807)	4	3	3	
Self-reported illness					
Myocardial infarction	3 (704)	4	2	2	<0.001
Stroke	1 (304)	2	1	1	0.005
Diabetes	2 (510)	3	2	2	<0.001
Energy (MJ/d)	8.00 (6.71, 9.56)	8.09 (6.72, 9.70)	7.77 (6.60, 9.19)	7.97 (6.77, 9.38)	<0.001
Protein (en%)	15.2 (13.6, 16.9)	15.1 (13.6, 16.9)	15.1 (13.5, 16.9)	15.4 (13.9, 17.0)	<0.001
Fat (en%)	33.4 (29.8, 36.8)	33.7 (30.2, 37.2)	33.0 (29.1, 36.4)	32.7 (29.3, 36.1)	<0.001
Saturated fat (en%)	12.7 (10.9, 14.6)	12.9 (11.1, 14.8)	12.5 (10.6, 14.5)	12.4 (10.6, 14.2)	<0.001
Carbohydrates (en%)	47.6 (43.4, 51.5)	47.2 (43.0, 51.1)	48.1 (44.0, 52.0)	48.2 (44.1, 52.0)	<0.001
Alcohol (en%)	2.0 (0, 5.9)	2.0 (0.0, 5.9)	2.1 (0.0, 5.9)	1.9 (0.0, 5.7)	0.62
Food intake (g/d)					
Fruit	153 (84, 242)	140 (71, 226)	170 (97, 258)	174 (102, 265)	<0.001
Vegetables	142 (102, 190)	138 (99, 186)	147 (105, 199)	146 (109, 195)	<0.001
Red & processed meat	53 (32, 76)	56 (35, 80)	45 (24, 67)	49 (29, 73)	<0.001
White meat	21 (6, 37)	21 (7, 37)	20 (7, 37)	21 (6, 38)	0.27
Oily fish, consumer (%)	54	52	58	58	<0.001
Consumers only	17 (10, 30)	17 (9, 30)	18 (10, 32)	19 (11, 32)	<0.001
White fish, consumer (%)	67	66	64	69	<0.001
Consumers only	19 (13, 29)	19 (14, 29)	19 (12, 29)	19 (12, 29)	0.021
Total fish, consumer (%)	84	83	84	86	<0.001
Consumers only	28 (17, 44)	27 (16, 43)	28 (17, 45)	30 (18, 46)	<0.001
Micronutrient intake					
Vitamin A (mcg/d), food	342 (232, 529)	350 (237, 542)	328 (222, 508)	330 (227, 507)	<0.001
Food + supplement	480 (278, 1047)	350 (237, 542)	578 (288 (1064)	1105 (914, 1523)	-
Vitamin D (mcg/d), food	2.78 (1.83, 4.21)	2.76 (1.80, 4.16)	2.67 (1.76, 4.16)	2.91 (1.94, 4.40)	<0.001
Food + supplement	3.70 (2.17, 6.04)	2.76 (1.80, 4.16)	4.19 (2.44, 6.37)	6.92 (5.09, 9.54)	-
Vitamin E (mcg/d), food	9.6 (7.4, 12.4)	9.5 (7.3, 12.4)	9.6 (7.4, 12.3)	9.8 (7.6, 12.6)	<0.001
Food + supplement	10.6 (7.9, 15.1)	9.5 (7.3, 12.4)	15.6 (10.3, 24.1)	13.1 (9.2, 19.1)	-
n-3 PUFA (g/d)					
Food	0.12 (0.06, 0.34)	0.12 (0.06, 0.31)	0.13 (0.06, 0.37)	0.14 (0.07, 0.39)	<0.001
Supplement	0 (0, 0)	0	0	0.10 (0.08, 0.16)	-
Food + supplement	0.16 (0.07, 0.40)	0.12 (0.06, 0.31)	0.13 (0.06, 0.37)	0.30 (0.17, 0.72)	-
Ratio supplement/ (food+supplement) (%)	0 (0, 0)	0	0	0.46 (0.21, 0.69)	-

Values are median (interquartile range) for continuous variables and % (n) for categorical variables.
n-3 PUFA, omega-3 polyunsaturated fatty acids (sum of eicosapentaenoic acid and docosahexaenoic acid); NSU, non-supplement users; SU-n3, non-n-3 PUFA supplement users; SU+n3, n-3 PUFA supplement users (mainly cod liver oil).

[a] Differences between groups were tested using Chi-squared statistic (categorical variables) and Kruskal-Wallis (continuous variables).

[b] Median and Interquartile Range (IQR) of age (years) was: NSU 59 (51, 67), SU-n3 57 (50, 66), SU+n3 62 (54, 68); P<0.001.

[c] Median (IQR) of BMI (kg/m^2) was: NSU 26.0 (23.9, 28.5), SU-n3 25.2 (23.1, 27.6), SU+n3 25.6 (23.6, 28.0); P<0.001.

For peer review only

For peer review only - http://bmjopen.bmj.com/site/about/guidelines.xhtml

Appendix IV: Hazard ratios for CHD mortality by supplement user subgroups assessed at DSA1 only.

Here we use data from DSA1 only and we ignored any changes over time for any of the included variables in the models. This shows the results if only baseline study measures would have been used. Median follow-up time was 19 years (1993-2015). The hazard of CHD death among SU+n3 was 20% lower than among NSU after adjusting for sex and age (model 1). Following adjustment for CHD risk factors, the association attenuated and became non-significant after adjustment for self-reported illnesses (models 2 and 3). Further adjustment for dietary variables minimally changed this HR (models 4 and 5). Equally, there was no evidence that the CHD mortality of SU-n3 differed from NSU using data from DSA1 only.

	All Case/N	NSU HR (Ref)	SU-n3 HR (95% CI)	SU+n3 HR (95% CI)
Case/n	1562/22,035	1012/13,444	178/3263	372/5328
Model 1: sex and age		1.00	0.86 (0.73, 1.01)	0.80 (0.71, 0.90)
Model 2: 1 + socio/lifestyle factors		1.00	0.93 (0.79, 1.09)	0.87 (0.77, 0.98)
Model 3: 2 + prevalent illnesses		1.00	0.96 (0.83, 1.13)	0.94 (0.83, 1.06)
Model 4: 3 + dietary factors (excluding fish)		1.00	0.98 (0.83, 1.15)	0.97 (0.86, 1.09)
Model 5: 4 + fish		1.00	0.98 (0.83, 1.15)	0.96 (0.85, 1.09)

NSU, non-supplement users; SU-n3, non-n-3 PUFA supplement users; SU+n3, n-3 PUFA supplement users (mainly cod liver oil); PYAR, person years at risk; CHD, coronary heart disease; HR, hazard ratio; 95%CI, 95% confidence interval.

Model 1: sex, age (continuous).
Model 2: Model 1 + smoking, BMI (continuous), alcohol intake (categorical), season in which 7dDD was completed, physical activity (inactive vs. some activity), social class (manual vs. non-manual), education level (no qualification vs. some qualification), marital status (not married vs. married).
Model 3: Model 2 + prevalent diabetes, myocardial infarction, stroke.
Model 4: Model 3 + energy intake, fruit, vegetable, red & processed meat, white meat (all continuous).
Model 5: Model 4 + white fish, oily fish (all continuous).

For peer review only

For peer review only - http://bmjopen.bmj.com/site/about/guidelines.xhtml

Appendix V: Fish or Supplement?

Characteristics of participants by quintiles of n-3 PUFA intake from food and supplements at DSA1 are provided in Appendix V-Table 1. Since fish is a source of n-3 PUFA, there was a strong association between fish consumption, particularly oily fish, and quintiles of n-3 PUFA intake from food and supplements. The percentage of fish consumers was 43% in quintile 1 and 98% in quintile 5.

It was observed that participants consuming a median intake of 0.81 g/d (5th quintile), had a 21% lower hazard of CHD mortality compared to very low consumers when adjusting for age and sex (Appendix V-Table 2); however, this association attenuated and became non-significant after adjusting for confounders and dietary variables, excluding fish (HR 0.93, 95%CI: 0.79, 1.09). When fish consumption was included, the HR for quintile 5 *vs.* 1 strengthened to 0.79 (95%CI: 0.64, 0.98). Equally, when the exposure was expressed as a continuous variable (per 1 g/d n-3 PUFA intake from food and supplements), we observed a stronger, borderline significant association when comparing model 4 (HR: 0.96, 95%CI: 0.85, 1.07) to model 5 (HR: 0.86, 95%CI: 0.74, 1.01).

In secondary analysis (Appendix V-Table 2), we excluded participants with prevalent baseline myocardial infarction, stroke and diabetes, by which potential increased fish consumption due to diagnosis could be dealt with. HRs strengthened and both quintile 4 (median of 0.33 g/d) and quintile 5 showed a significant lower hazard of CHD mortality compared to quintile 1.

In summary, an inverse association between quintiles of n-3 PUFA intake and CHD mortality was only observed after adjustment for fish consumption, indicating that food and supplement sources of n-3 PUFA might be differently associated with CHD mortality.

However, data on fish consumption (and hence n-3 PUFA intake from food) from 7-day diet diaries could only be estimated for DSA1. Within person changes in fish consumption were unknown and the lack of such data might have misclassified participants over time; or, our results might have been confounded if fish consumption over time was associated with risk factors of CHD mortality, *e.g.* a non-fatal myocardial infarction might have led to increased fish consumption, thereby associating higher fish or n-3 PUFA intake with higher risk of CHD mortality. This 'confounding by indication' might have been the reason why HRs between

For peer review only - http://bmjopen.bmj.com/site/about/guidelines.xhtml

n-3 PUFA intake from food and supplements and CHD mortality strengthened after excluding prevalent illnesses.

Fish consumption was encouraged (inter)nationally during the follow-up time [13,14,66]; however, national survey data do not indicate increased fish consumption [4,52], although a decrease in the duration of data collection from 7 to 4-day diary records limits this comparison.

Fish contains both nutrients and contaminants, such as methylmercury, dioxin or dioxin like polychlorinated biphenyls (PCB), which have been associated with the CHD, but in opposing directions [66–69]. Fish oil supplements are not free of such contaminants, but might contain lower concentrations because of their production process [50]. This might explain why adjusting for fish consumption, *i.e.* a relative larger proportion of the higher n-3 PUFA intake being derived from supplements, was associated with a lower hazard of CHD mortality and why fish was associated with a higher hazard of CHD mortality.

Fish consumption advice has been given which maximises n-3 PUFA intake, while minimising methylmercury content for the general population, since the lower hazard of CHD mortality observed for higher n-3 PUFA concentrations only became apparent after controlling for methylmercury contamination [67,69,70].

For peer review only

For peer review only - http://bmjopen.bmj.com/site/about/guidelines.xhtml

Appendix V-Table 1: Characteristics of participants by n-3 PUFA intake from food and supplement as assessed at DSA1 (1993-1998). Values are percent or Median (IQR).

Median n-3 PUFA from food and supplements (IQR), g/d	Quintile 1<br>0.04 (0.02, 0.05)	Quintile 2<br>0.09 (0.07, 0.10)	Quintile 3<br>0.16 (0.14, 0.19)	Quintile 4<br>0.33 (0.27, 0.40)	Quintile 5<br>0.81 (0.62, 1.17)
CHD mortality	287/4400	293/4417	338/4410	313/4410	331/4398
Crude rate (1000 PYAR)	3.728	3.809	4.413	4.087	4.335
Sex					
Men	40	47	45	44	49
Women	60	53	55	56	51
Age [b]					
=<50 years	28	24	19	17	14
>50-60 years	33	32	33	30	31
>60-70 years	26	29	32	37	37
>70 years	14	15	17	16	18
BMI [c]					
=<20 kg/m^2	3	2	2	2	2
>20-25 kg/m^2	40	37	38	37	39
>25-30 kg/m^2	41	46	46	47	47
>30 kg/m^2	16	15	15	15	12
Smoking status					
Current	14	13	11	9	8
Former	39	43	43	44	45
Never	47	45	46	47	47
Social class					
Professional	7	5	6	7	9
Managerial	34	35	36	38	41
Skilled non-manual	16	16	18	18	16
Skilled manual	25	25	23	22	20
Semi-skilled	14	15	14	12	11
Non-skilled	4	4	4	3	2

For peer review only

For peer review only - http://bmjopen.bmj.com/site/about/guidelines.xhtml

Median n-3 PUFA from food and supplements (IQR), g/d	Quintile 1<br>0.04 (0.02, 0.05)	Quintile 2<br>0.09 (0.07, 0.10)	Quintile 3<br>0.16 (0.14, 0.19)	Quintile 4<br>0.33 (0.27, 0.40)	Quintile 5<br>0.81 (0.62, 1.17)
Marital status					
Married	82	83	82	83	82
Not married	19	18	18	17	18
Education level					
No qualification	37	38	38	36	32
O-level	11	11	10	10	10
A-level	39	40	40	41	43
Degree	12	11	12	13	15
Season of 7dDD					
Spring	25	26	27	28	26
Summer	23	24	24	27	27
Autumn	29	26	25	24	25
Winter	24	24	23	21	23
Physical activity					
Inactive	30	31	30	31	28
Moderately inactive	28	28	30	29	30
Moderately active	23	23	23	22	24
Active	19	18	17	18	18
Alcohol intake (HLQ)					
None	16	14	14	13	11
>0-14 units/wk	73	73	72	71	71
>14-28 units/wk	8	10	11	12	14
>28 units/wk	3	4	3	4	4
Self-reported illness					
Myocardial infarction	3	4	3	3	4
Stroke	2	1	1	1	2
Diabetes	2	2	3	2	3

For peer review only - http://bmjopen.bmj.com/site/about/guidelines.xhtml

	Quintile 1	Quintile 2	Quintile 3	Quintile 4	Quintile 5
**Median n-3 PUFA from food and supplements (IQR), g/d**	**0.04 (0.02, 0.05)**	**0.09 (0.07, 0.10)**	**0.16 (0.14, 0.19)**	**0.33 (0.27, 0.40)**	**0.81 (0.62, 1.17)**
Energy (MJ/d)	7.61 (6.32, 9.06)	8.03 (6.74, 9.61)	8.08 (6.76, 9.63)	8.04 (6.80, 9.60)	8.28 (6.99, 9.84)
Protein (en%)	14.5 (130, 16.3)	15.0 (13.4, 16.7)	15.2 (13.7, 16.9)	15.4 (13.9, 17.1)	15.7 (14.3, 17.5)
Fat (en%)	33.1 (29.6, 36.7)	33.9 (30.2, 37.2)	33.6 (30.1, 37.0)	33.2 (29.5, 36.5)	33.2 (29.6, 36.8)
Saturated fat (en%)	12.9 (11.1, 14.7)	13.0 (11.2, 14.9)	12.8 (11.1, 14.7)	12.6 (10.8, 14.5)	12.3 (10.4, 14.2)
Carbohydrates (en%)	48.9 (44.6, 53.0)	47.7 (43.7, 51.4)	47.5 (43.3, 51.2)	47.3 (43.1, 51.0)	46.5 (42.1, 50.6)
Alcohol (en%)	1.3 (0.0, 5.0)	1.7 (0.0, 5.1)	2.0 (0.0, 5.7)	2.4 (0.0, 6.5)	2.7 (0.3, 6.9)
Food intake (g/d)					
Fruit	134 (64, 220)	136 (68, 218)	150 (79, 234)	162 (92, 253)	186 (109, 275)
Vegetables	132 (93, 177)	134 (96, 180)	136 (100, 184)	149 (108, 196)	159 (117, 212)
Red & processed meat	53 (31, 76)	58 (37, 81)	54 (34, 78)	51 (31, 73)	48 (26, 74)
White meat	18 (3, 34)	21 (7, 37)	21 (8, 38)	22 (8, 38)	21 (5, 39)
Oily fish	0 (0, 0)	0 (0, 4)	0 (0, 9)	15 (8, 22)	31 (21, 48)
Consumers (%)	15	30	49	85	93
Consumers only	7 (4, 11)	9 (5, 14)	9 (6, 15)	17 (12, 24)	34 (24, 50)
White fish	0 (0, 11)	16 (10, 22)	19 (3, 30)	17 (0, 28)	15 (0, 27)
Consumers (%)	32	80	78	75	68
Consumers only	11 (11, 16)	9 (15, 25)	21 (16, 34)	21 (15, 34)	21 (15, 33)
Dietary supplement					
NSU	79	75	51	54	45
SU-n3	19	16	12	14	13
SU+n3	2	9	37	31	42
n-3 PUFA (g/d)					
Food	0.04 (0.02, 0.05)	0.08 (0.07, 0.10)	0.14 (0.09, 0.17)	0.30 (0.24, 0.38)	0.66 (0.52, 0.90)
Food + supplement	0.04 (0.02, 0.05)	0.09 (0.07, 0.10)	0.16 (0.14, 0.19)	0.33 (0.27, 0.40)	0.81 (0.62, 1.17)
Ratio supplement/(food+supplement) (%)	0 (0, 0)	0 (0, 0)	0 (0, 48)	0 (0, 21)	0 (0, 16)

n-3 PUFA, marine omega-3 polyunsaturated fatty acids EPA and DHA; IQR, interquartile range.

For peer review only - http://bmjopen.bmj.com/site/about/guidelines.xhtml

Appendix V-Table 2: The association between quintiles of the sum of food and supplement n-3 PUFA at DSA1 and subsequent hazard of CHD mortality (where cause of death was mentioned anywhere on the death certificate). The analyses relied on a median of 19 years of follow-up (1993-2015).

	Quintile 1	Quintile 2	Quintile 3	Quintile 4	Quintile 5	Continuous
Median food+supplement n-3 PUFA (IQR) g/d	0.04 (0.02, 0.05)	0.09 (0.07, 0.10)	0.16 (0.14, 0.19)	0.33 (0.27, 0.40)	0.81 (0.62, 1.17)	Per 1 g/d
Median food n-3 PUFA (IQR) g/d	0.04 (0.02, 0.05)	0.08 (0.07, 0.10)	0.14 (0.09, 0.17)	0.30 (0.24, 0.38)	0.66 (0.52, 0.90)	
Median ratio food:(food+supplement) (IQR) %	100 (100, 100)	100 (100, 100)	100 (52, 100)	100 (79, 100)	100 (84, 100)	
Median white fish (IQR) g/d	0 (0, 11)	16 (10, 22)	19 (6, 43)	17 (0, 28)	15 (0, 27)	
Median oily fish (IQR) g/d	0 (0, 0)	0 (0, 4)	0 (0, 9)	15 (8, 22)	31 (21, 48)	
	**HR**	**HR (95% CI)**	**HR (95% CI)**	**HR (95% CI)**	**HR (95% CI)**	**HR (95% CI), P**
Case/n	287/4407	293/4407	338/4407	312/4407	332/4407	1562/22,035
Model 1: sex and age	1.00	0.91 (0.77, 1.07)	0.95 (0.81, 1.11)	0.83 (0.71, 0.97)	0.80 (0.68, 0.93)	0.87 (0.77, 0.97), P=0.013
Model 2: 1 + socio/lifestyle factors	1.00	0.91 (0.77, 1.07)	1.00 (0.85, 1.17)	0.89 (0.76, 1.04)	0.87 (0.74, 1.02)	0.92 (0.83, 1.03), P=0.17
Model 3: 2 + prevalent illnesses	1.00	0.89 (0.76, 1.05)	1.02 (0.87, 1.20)	0.91 (0.78, 1.07)	0.88 (0.75, 1.03)	0.92 (0.82, 1.03), P=0.15
Model 4: 3 + dietary factors (excluding fish)	1.00	0.91 (0.77, 1.07)	1.05 (0.90, 1.24)	0.96 (0.82, 1.13)	0.93 (0.79, 1.10)	0.96 (0.85, 1.07), P=0.45
Model 5: 4 + fish	1.00	0.88 (0.74, 1.04)	1.00 (0.85, 1.18)	0.88 (0.74, 1.05)	0.79 (0.64, 0.98)	0.86 (0.74, 1.01), P=0.07
Case/n	217/4156	212/4122	257/4125	231/4139	242/4098	1159/20,640
Model 5, excluding illness at DSA1	1.00	0.86 (0.71, 1.05)	0.96 (0.79, 1.16)	0.79 (0.64, 0.97)	0.70 (0.55, 0.90)	0.86 (0.71, 1.03), P=0.09

DSA, dietary supplement assessment; n-3 PUFA, omega-3 polyunsaturated fatty acids (sum of EPA and DHA); IQR, interquartile range; CHD, coronary heart disease; HR, hazard ratio; 95%CI, 95% confidence interval.

See also Appendix V for detailed description of the cohort by quintiles of n-3 PUFA exposure at DSA1.

Model 1: sex, age (continuous).
Model 2: Model 1 + smoking, BMI (continuous), alcohol intake, season in which 7dDD was completed, physical activity (inactive vs. some activity), social class (manual vs. non-manual), education level (no qualification vs. some qualification), marital status (not married vs. married).
Model 3: Model 2 + adjusted for prevalent diabetes, myocardial infarction and stroke.
Model 4: Model 3 + energy intake, fruit, vegetable, red & processed meat, white meat (all continuous).
Model 5: Model 4 + white fish (HR per 80g/d: 1.20, 95%CI: 0.94, 1.52), oily fish (HR per 80 g/d: 1.33, 95%CI: 1.02, 1.72).
Model 5, excluding baseline myocardial infarction, stroke, diabetes: white fish (HR per 80g/d: 1.23, 95%CI: 0.93, 1.61), oily fish (HR per 80 g/d: 1.66, 95%CI: 1.21, 2.28).

For peer review only - http://bmjopen.bmj.com/site/about/guidelines.xhtml

Appendix VI: Interactions using time-varying covariate analysis for the association between CLO consumption and risk of CHD mortality (1993-2015).

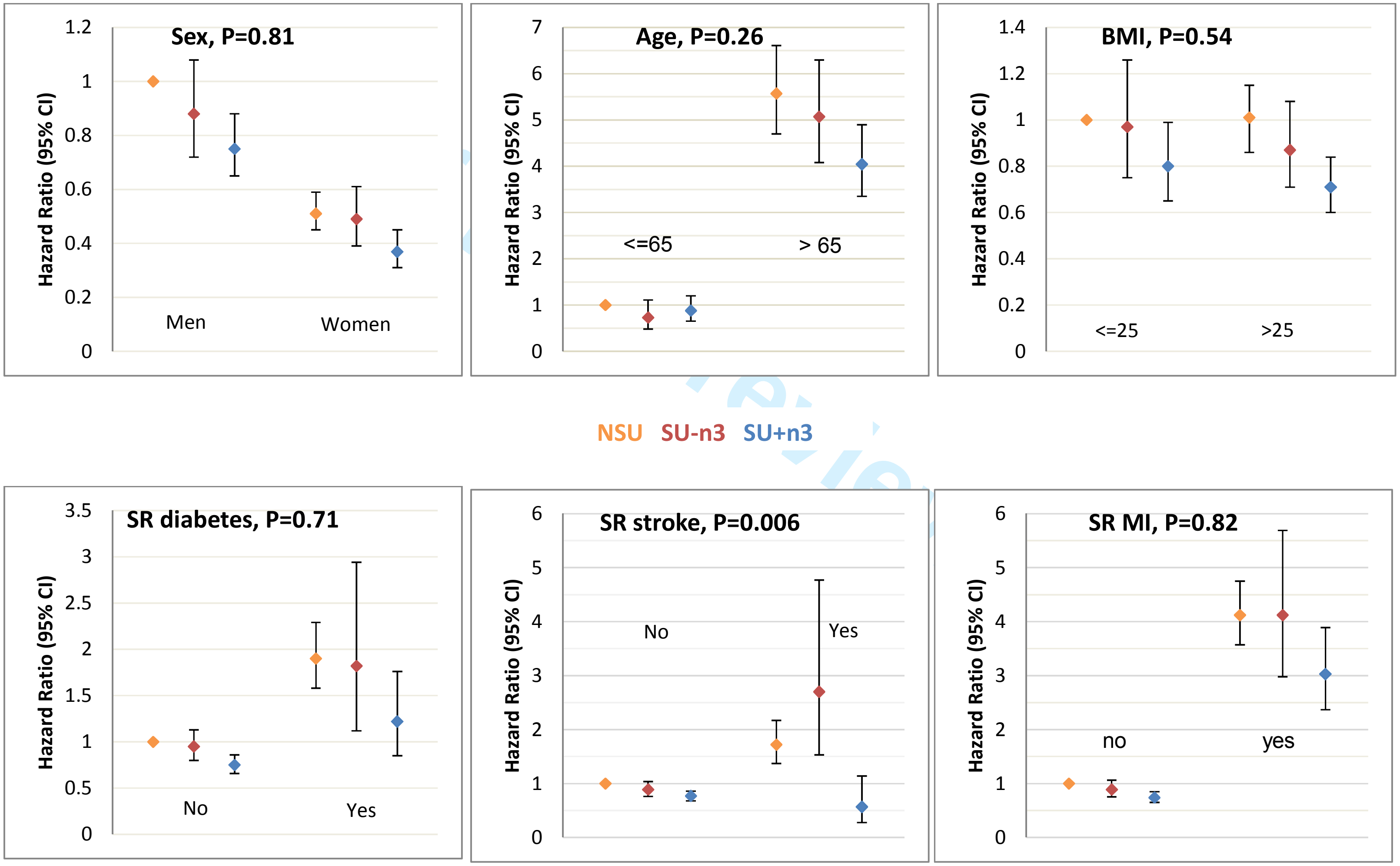

For peer review only - http://bmjopen.bmj.com/site/about/guidelines.xhtml

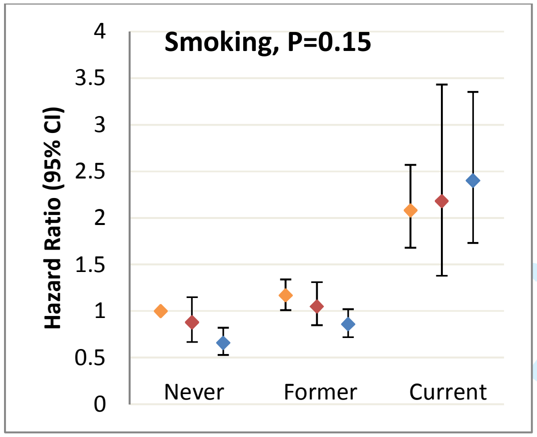

NSU SU-n3 SU+n3

BMI, body mass index (kg/m^2); SR, self-reported; MI, myocardial infarction. ***Note***: the Y-axis for each graph is different.

All Models (1640/24,329) in time-varying covariates analysis were adjusted for: time-point specific age, smoking, BMI, alcohol consumption, physical activity, season of questionnaire completion, marital status and self-report of myocardial infarction, stroke or diabetes; as well as: sex, social class and education at time of DSA1.

The P-value represents the significance of the model's improvement after including the interaction term with supplement use and the presented variable.

For peer review only - http://bmjopen.bmj.com/site/about/guidelines.xhtml

Appendix VII: Multiple Imputation

Multiple imputation was used to handle missing data in time-fixed variables and in longitudinal exposures. For the longitudinal variables, multiple imputation is a superior alternative to the last-observation carried forward approach used in the main analyses (Figure 1).

Appendix VII-Table 1 shows the number of missing values for each included variable in adjustment model 3. DSA1 had the highest number of variables with missing values, particularly for supplement use (10%), since supplement use was obtained as part of a short questionnaire on the last pages of the 7dDD booklet; participants who did not complete all seven days were most likely to skip these questions. At DSA2 and DSA3, BMI had the most missing values, 28% and 22% respectively. At DSA2, this was caused by BMI being obtained from an earlier time point than the questionnaire (with non-response for HE2, see Appendix I-Table 1); at DSA3, not all participants were seen at HE3 due to time/cost restrictions of the study.

Univariable logistic regression was applied to study associations between each variable and missingness in the supplement use variable (Appendix VII-Table 1). Only at DSA1 were significant associations observed between variables included in Model 3 of the analysis and missingness for supplement use. Being a man, an alcohol consumer, unmarried, smoker or heavier were associated with more missingness of supplement use; being older was associated with less missingness of supplement use. Dietary variables were only available at DSA1, these had strong associations with missingness for the supplement use variable

Missing supplement use at DSA1 (n=2619) was not associated with CHD mortality (HR=0.87; 95%CI: 0.74, 1.02). Only 4 and 11 participants had missing data on supplement use at DSA2 and DSA3 respectively.

Missing values in several variables were handled using multiple imputation by chained equations (MICE). We used the imputation approach described by White and Royston [71] for imputation of missing data in explanatory variables used in Cox regression, by including the event indicator and the Nelson-Aalen estimate of the cumulative hazard at the event or censoring time as predictors in all imputation models, in addition to the predictors summarised below. After the imputation, the aim is to estimate the association between

For peer review only - http://bmjopen.bmj.com/site/about/guidelines.xhtml

CLO group and the outcome (CHD mortality) using adjustment model 3. The time-fixed variables included in adjustment model 3 are sex, age, education, social class, and the time-dependent variables are supplement use, smoking, BMI, physical activity, alcohol consumption, season of questionnaire completion and marital status. Missing data in time-dependent variables was handled using a modified version of the two-fold fully conditional specification approach suggested by Nevalainen *et al.* [72]. Time-fixed variables were imputed using time-dependent variables measured at DSA1 as predictors. Time-dependent variables at DSA1 were imputed using the time-fixed variables and other time-dependent variables from DSA1 as predictors. A given time-dependent variable at DSA2 was imputed using the same variable from DSA1 as a predictor, alongside the other time-dependent variables from DSA2 and the time-fixed variables. Similarly, a given time-dependent variable at DSA3 was imputed using the same variable from DSA2 as a predictor, alongside the other time-dependent variables from DSA3 and the time-fixed variables. In summary, a given time-dependent variable was imputed using time-fixed variables, the most recent past value of the same variable, and concurrent values of other time-dependent variables, in addition to the event indicator and the Nelson-Aalen estimate of the cumulative hazard at the event or censoring time. Nevalainen *et al.* suggested including both past and future values of time-dependent variables in the imputation model for a given time-dependent variable [72]; however, this caused convergence issues in our setting and so we simplified the procedure to only use past values. The multiple imputation was performed using *mi impute* in Stata v14. Twenty imputed data sets were created (the percentage of participants with minimally one missing value was 14%, 30% and 25% at each DSA respectively) [73]. Imputed values were used in Cox regression models and the resulting estimated log hazard ratios were combined using Rubin's Rules. Cox proportional hazards analysis was applied to replicate results reported in Table 2 of the main manuscript (with CHD mortality reported on the death certificate [anywhere] as the outcome).

The results are shown in Appendix VII-Table 2. Results using the imputed dataset and results from the main manuscript had similar HRs. As expected, the confidence intervals obtained after multiple imputation were smaller than those obtained previously, and hence some borderline non-significant associations became borderline significant associations.

For peer review only - http://bmjopen.bmj.com/site/about/guidelines.xhtml

Our conclusions are therefore unchanged: supplements containing n-3 PUFA (mainly low dose cod liver oil in this cohort) were associated with a lower hazard of CHD mortality.

Appendix VII-Table 1: Number of missing values for each variable and the association (using univariable logistic regression) between this variable and missingness for the supplement use variable.

	DSA1		DSA2		DSA3	
	N	OR (95%CI)	N	OR (95%CI)	N	OR (95%CI)
Attended HE1	25,639					
Completed questionnaire			17,585		10,874	
Supplement use	2619	-	11	-	4	-
Social class	570	1.06 (0.97, 1.15)	313	1.21 (0.27, 5.43)	143	0.89 (0.08, 9.77)
Alcohol	268		156		282	
>0-14 units/wk		1.18 (1.03, 1.34)		0.48 (0.11, 2.12)		-
>14-28 units/wk		1.58 (1.34, 1.86)		2.03 (0.45, 9.08)		
>28 units/wk		1.71 (1.38, 2.14)		2.19 (0.24, 19.67)		
Smoking	220		91		4	-
current		1.95 (1.74, 2.19)		-		
former		1.06 (0.97, 1.16)		0.83 (0.12, 5.88)		
Marital status	150	1.12 (1.02, 1.24)	138	-	104	-
Seasons	132		0	-	0	-
Spring		0.97 (0.86, 1.09)				
Summer		0.90 (0.80, 1.02)				
Autumn		1.01 (0.89, 1.13)				
BMI (kg/m^2)	57	1.05 (1.04, 1.06)	4864	0.98 (0.83, 1.15)	2393	0.84 (0.60, 1.18)
Physical activity	19	0.97 (0.89, 1.06)	0	1.06 (0.31, 3.62)	1	-
Education	18	1.11 (1.02, 1.21)	6	0.33 (0.06, 1.99)	3	-
Self-reported						
MI	13	0.86 (0.67, 1.10)	0	-	0	-
diabetes	13	0.78 (0.58, 1.05)	0	1.92 (0.25, 15.4)	0	2.56 (0.32, 20.49)
stroke	13	1.06 (0.76, 1.48)	0	-	0	-
Sex	0	1.36 (1.25, 1.48)	0	1.59 (0.48, 5.20)	0	1.30 (0.18, 9.23)
Age	0	0.97 (0.97, 0.97)	0	1.04 (0.97, 1.11)	0	1.06 (0.95, 1.20)
Energy (MJ/d)	132	0.96 (0.95, 0.98)	-		-	
Foods (g/d)						
Oily fish	132	0.53 (0.44, 0.65)	-		-	
White fish	132	0.36 (0.29, 0.45)	-		-	
Red & processed meat	132	1.30 (1.19, 1.41)	-		-	
Fruit	132	0.83 (0.81, 0.86)	-		-	
Vegetables	132	0.88 (0.84, 0.92)	-		-	

For peer review only

For peer review only - http://bmjopen.bmj.com/site/about/guidelines.xhtml

Appendix VII-Table 2: The association between supplement use reported at DSA1, DSA2 and DSA3 and subsequent hazard of CHD mortality (where cause of death was mentioned anywhere on the death certificate). The follow-up time in the EPIC-Norfolk study was from 1993 to 2015. Results obtained from multiple imputation as described above (these results may be compared to Table 2 of the main manuscript).

		Two years of follow-up		Four years of follow-up		Follow-up time until next DSA*		Full follow-up time	
	Total N	CHD Events N	HR (95% CI)	CHD Events N	HR (95% CI)	CHD Events N	HR (95% CI)	CHD Events N	HR (95%CI)
DSA1 (1993-1998)	25,621	99		234		1053		**1813	
NSU			1.00		1.00		1.00		1.00
SU-n3			1.18 (0.59, 2.35)		1.24 (0.80, 1.91)		1.04 (0.84, 1.27)		0.96 (0.82, 1.12)
SU+n3			0.92 (0.53, 1.58)		1.06 (0.76, 1.48)		0.84 (0.71, 0.99)		0.92 (0.81, 1.04)
DSA2 (2002-2004)	17,585	87		196		604		832	
NSU			1.00		1.00		1.00		1.00
SU-n3			0.98 (0.51, 1.88)		1.19 (0.79, 1.81)		0.94 (0.72, 1.21)		0.83 (0.66, 1.04)
SU+n3			0.54 (0.29, 0.98)		0.67 (0.46, 0.98)		0.74 (0.61, 0.91)		0.81 (0.69, 0.96)
DSA3 (2004-2011)	10,874	65		137		261			
NSU			1.00		1.00		1.00		
SU-n3			0.60 (0.26, 1.42)		1.12 (0.69, 1.81)		1.07 (0.74, 1.53)		
SU+n3			0.45 (0.23, 0.88)		0.58 (0.37, 0.89)		0.72 (0.53, 0.97)		
Time-varying	27,796	251		567		**1918			
NSU			1.00		1.00		1.00		
SU-n3			0.93 (0.61, 1.41)		1.19 (0.92, 1.53)		0.92 (0.79, 1.06)		
SU+n3			0.63 (0.45, 0.89)		0.78 (0.63, 0.97)		0.74 (0.66, 0.84)		

All models adjusted (model 3) for time-point specific (and imputed where necessary): age, smoking, BMI, alcohol consumption, physical activity, season of questionnaire completion, marital status and self-report of myocardial infarction, stroke or diabetes; as well as: sex, social class and education.
*or in case of DSA3, the censor date was the date of administrative follow-up (31 March 2015).
**Since participants can contribute follow-up time at DSA2 and/or DSA3 only (without contributing to DSA1), the number of participants/deaths included in the time-varying analysis is larger than reported in DSA1 only.

For peer review only - http://bmjopen.bmj.com/site/about/guidelines.xhtml

BMJ Open

Longitudinal associations between marine omega-3 supplement users and Coronary Heart Disease in a UK population-based cohort

Journal:	*BMJ Open*
Manuscript ID	bmjopen-2017-017471.R1
Article Type:	Research
Date Submitted by the Author:	21-Jun-2017
Complete List of Authors:	Lentjes, Marleen; University of Cambridge, Department of Public Health & Primary Care<br>Keogh, Ruth; London School of Hygiene and Tropical Medicine, Medical Statistics<br>Welch, A. A.; University of East Anglia, Population Health & Primary Care<br>Mulligan, Angela; University of Cambridge, Public Health & Primary Care<br>Luben, Robert; University of Cambridge, Department of Public Health and Primary Care<br>Wareham, Nicholas; University of Cambridge School of Clinical Medicine, MRC Epidemiology Unit<br>Khaw, Kay-Tee; University of Cambridge, Clinical Medicine
**Primary Subject Heading**:	Epidemiology
Secondary Subject Heading:	Nutrition and metabolism, Public health, Research methods, Cardiovascular medicine
Keywords:	EPIDEMIOLOGY, Cardiac Epidemiology < CARDIOLOGY, NUTRITION & DIETETICS

SCHOLARONE™
Manuscripts

Longitudinal associations between marine omega-3 supplement users and Coronary Heart Disease in a UK population-based cohort

Marleen AH Lentjes*: Snr. Research Nutritionist, Department of Public Health and Primary Care, University of Cambridge, Strangeways Research Laboratories, Worts Causeway, CAMBRIDGE CB1 8RN, United Kingdom. Email: marleen.lentjes@phpc.cam.ac.uk. Telephone: 01223 748683

Ruth H Keogh: Associate Professor, Department of Medical Statistics, London School of Hygiene and Tropical Medicine, Keppel street, LONDON WC1E 7HT, United Kingdom.

Ailsa A Welch: Reader in Nutritional Epidemiology, Department of Population Health & Primary Care, University of East Anglia, NORWICH NR4 7TJ, United Kingdom.

Angela A Mulligan: Snr. Research Nutritionist, Department of Public Health and Primary Care, University of Cambridge, Strangeways Research Laboratories, Worts Causeway, CAMBRIDGE CB1 8RN, United Kingdom.

Robert N Luben: Head of Bioinformatics, Department of Public Health and Primary Care, University of Cambridge, Strangeways Research Laboratories, Worts Causeway, CAMBRIDGE CB1 8RN, United Kingdom.

Nicholas J Wareham: Director MRC Epidemiology Unit, University of Cambridge, Institute of Metabolic Science, CAMBRIDGE CB2 0SL, United Kingdom.

Kay-Tee Khaw: Professor of Clinical Gerontology, University of Cambridge, School of Clinical Medicine, Addenbrooke's Hospital, CAMBRIDGE CB2 2QQ, United Kingdom.

*Corresponding author

For peer review only

Contributorship statement

MAHL: wrote the manuscript, prepared & analysed both the supplement and dietary data. Developed the research question.

RHK: advised on statistical analysis, specifically time-varying covariates analysis and multiple imputation.

AAW: Managed the dietary data collection of the study. Contributed to the study protocol.

AAM: Prepared dietary data.

RNL: Manages study data. Contributed to the study protocol.

NJW: Principal investigator of the study.

KTK: Principal investigator of the study.

All authors critically read and contributed to the manuscript.

Declaration of competing interests

All authors have completed the ICMJE uniform disclosure form at http://www.icmje.org/coi_disclosure.pdf and declare: no support from any organisation for the submitted work; no financial relationships with any organisations that might have an interest in the submitted work in the previous three years, no other relationships or activities that could appear to have influenced the submitted work.

Transparency declaration

Prof. Khaw affirms that the manuscript is an honest, accurate, and transparent account of the study being reported; no important aspects of the study have been omitted; any discrepancies from the study have been explained.

All authors had full access to all of the data (including statistical reports and tables) in the study and can take responsibility for the integrity of the data and the accuracy of the data analysis.

For peer review only

For peer review only - http://bmjopen.bmj.com/site/about/guidelines.xhtml

Ethics

Study approval was obtained from the Norfolk District Health Authority Ethics Committee. All participants completed a written informed consent.

Funding

All authors report grants from Cancer Research UK programme grants (G0401527, G1000143) and grants from the Medical Research Council (MRC) programme grants (C864/A8257, C864/A14136) during the study. RHK is supported by a MRC Fellowship (MR/M014827/1).

The funders of EPIC-Norfolk had no role in the study design, data collection, data analysis, interpretation of data, writing of the report, or in the decision to submit the paper for publication.

Data sharing statement

More information on the EPIC-Norfolk study can be obtained from the website: www.epic-norfolk.org.uk. Requests for collaboration will be discussed at the EPIC-Norfolk management meetings.

Exclusive license

The Corresponding Author has the right to grant on behalf of all authors and does grant on behalf of all authors, a worldwide licence (http://www.bmj.com/sites/default/files/BMJ%20Author%20Licence%20March%202013.doc) to the Publishers and its licensees in perpetuity, in all forms, formats and media (whether known now or created in the future), to i) publish, reproduce, distribute, display and store the Contribution, ii) translate the Contribution into other languages, create adaptations, reprints, include within collections and create summaries, extracts and/or, abstracts of the Contribution and convert or allow conversion into any format including without limitation audio, iii) create any other derivative work(s) based in whole or part on the on the Contribution, iv) to exploit all subsidiary rights that currently exist or as may exist in the future in the Contribution, v) the inclusion of electronic links from the Contribution to third party material where-ever it may be located; and, vi) licence any third party to do any or all of the above.

Acknowledgements

The authors thank the EPIC-Norfolk participants and the research and administrative staff for their contributions, past & present.

For peer review only

Abstract

Objectives: Assess the association between marine omega-3 polyunsaturated fatty acid (n-3 PUFA) intake from supplements, mainly cod liver oil, and coronary heart disease (CHD) mortality.

Design: Prospective cohort study, with three exposure measurements over 22 y.

Setting: Norfolk-based European Prospective Investigation into Cancer (EPIC-Norfolk, UK).

Participants: 22,035 men and women from the general population, 39-79 y at recruitment.

Exposure: Supplement use was assessed in three questionnaires (1993-1998; 2002-2004; 2004-2011). Participants were grouped into non-supplement users (NSU), n-3 PUFA supplement users (SU+n3) and non n-3 PUFA supplement users (SU-n3). Cox regression adjusted for time-point specific variables: age, smoking, prevalent illnesses, BMI, alcohol consumption, physical activity and season and baseline assessments of sex, social class, education and dietary intake (7-day diet diary).

Primary and secondary outcome measures: During a median of 19 y follow-up, 1562 CHD deaths were registered for 22,035 included participants.

Results: Baseline supplement use was not associated with CHD mortality, but baseline food and supplement intake of n-3 PUFA was inversely associated with CHD mortality after adjustment for fish consumption. Using time-varying covariate analysis, significant associations were observed for SU+n3 (HR: 0.74, 95%CI: 0.66, 0.84), but not for SU-n3 *vs*. NSU. In further analyses, the association for SU+n3 persisted in those who did not take other supplements (HR: 0.83, 95%CI: 0.71, 0.96) and those who did (HR: 0.74, 95%CI: 0.60, 0.91). Those who became SU+n3 over time or were consistent SU+n3 *vs*. consistent NSU had a lower hazard of CHD mortality; no association with CHD was observed in those who stopped using n-3 PUFA-containing supplements.

Conclusions: Recent use of n-3 PUFA supplements was associated with a lower hazard of CHD mortality in this general population with low fish consumption. Residual confounding cannot be excluded, but the findings observed may be explained by postulated biological mechanisms and the results were specific to SU+n3.

For peer review only

For peer review only - http://bmjopen.bmj.com/site/about/guidelines.xhtml

Article summary

Strengths and limitations of this study

- The use of dietary supplements was measured three times over the course of 22 years.
- The repeated measures enabled to study change in supplement use in relation to hard endpoints.
- The cohort was population-based as opposed to trials on omega-3 supplement use which have been in high risk populations.
- Total omega-3 intake from both food (mainly fish sources) and supplement sources could be quantified; however, only for the baseline measure.
- Estimates were adjusted for socio-demographic and other behavioural risk factors, although residual confounding cannot be excluded.

For peer review only

For peer review only - http://bmjopen.bmj.com/site/about/guidelines.xhtml

Introduction

116 The traditionally high omega-3 polyunsaturated fatty acid (n-3 PUFA) intake among the Inuit has been associated with a lower risk of ischemic heart disease mortality in this population [1]. Research leading on from this observed that in a Dutch cohort, where average fish consumption was 20 g/d, higher fish consumption was associated with lower relative risk of Coronary Heart Disease (CHD) mortality between 1960-1980 [2]. A recent review of prospective cohort studies has observed a 16% (95%CI: 5-25%) lower risk of CHD mortality in those consuming one portion of fish per week *vs.* lower frequency of consumption; however, null findings among included studies were equally observed [3]. The association between fish intake and CHD has been attributed to n-3 PUFA, which are mainly derived from oily fish. According to the National Diet and Nutrition Survey in 2000/'01, less than 50% of the UK population consumed oily fish once a week [4]; however, dietary supplements may provide 50% of the n-3 PUFA intake [5].

N-3 PUFA at pharmaceutical doses reduce plasma triglycerides and possibly thrombosis; whereas lower doses (around 250 mg/d, obtainable from fish consumption and low dose supplements such as cod liver oil [CLO]) have been observed to lower the risk of sudden death or CHD death in trials and observational studies, which may be due to the influence of n-3 PUFA on heart rate and arrhythmia [6–8]. Reviews of trials on n-3 PUFA supplementation in relation to overall cardiovascular diseases (CVD) [9], or when separated into subgroups of stroke and coronary diseases [10–12], have shown no overall benefit, particularly in the most recent trials. It has been suggested that background medication, inclusion of non-fatal events as outcome measures and increasing trends in n-3 PUFA/fish consumption might underlie the null findings [13,14].

Although there have been a number of studies on the association between n-3 PUFA intake and CHD, the evidence for a protective association between n-3 PUFA supplement intake and CHD remains inconclusive [15]. Consumption of n-3 PUFA supplements is traditionally high in Northern European countries [16]. A Swedish cohort observed no association for fish oil supplements and CVD mortality [17]; whereas a study based in Iceland observed a lower risk of CHD hospitalisation with high frequency of CLO consumption [18]. Previous studies have relied on single baseline measures of supplement use and covariates [19],

For peer review only

excluded fish oil supplement users because of minimal use in the studied cohort [20] or lacked detailed data on supplement use and imputed n-3 PUFA content [21]; moreover, trials have included participants at increased risk of CHD [22–24] or included those who have already experienced a CHD event [25–28]; the effects of n-3 PUFA supplementation in a general population are less well documented, and the first primary prevention trial is ongoing [29].

The aim of this analysis was to use three repeated measures of supplement use to investigate the association between n-3 PUFA supplement intake and CHD mortality in a general population-based cohort.

Methods

An extended version of the methods has been included in Appendix I.

Study design

Recruitment for the Norfolk-based European Prospective Investigation into Cancer and Nutrition (EPIC-Norfolk) started in 1993 and was completed in 1998 [30,31]. Thirty-five general practices in the Norfolk area of East-Anglia (UK) took part in the study; 77,630 registered patients were approached, 30,445 consented. Exposures have been re- assessed over the course of the study; participants completed general Health and Lifestyle Questionnaires (HLQ) and attended three health examinations (HE) up to 2011 (Figure 1, Appendix I). Data on health outcomes and mortality were collected passively. Ethical approval for the study was given by the Norwich District Health Authority Ethics Committee. Participants gave written informed consent.

Assessment of n-3 PUFA supplement use

Dietary supplements were defined according to the EU directive 2002/46/EC [32]. Prescribed medication containing minerals and/or vitamins were not considered supplements. The sum of eicosapentaenoic acid (EPA) and docosahexaenoic acid (DHA) from supplements were the main exposure, referred to as n-3 PUFA. We used data from three dietary supplement assessments (DSA), named DSA1, DSA2, DSA3. All covered a one week recall (Appendix I).

For peer review only - http://bmjopen.bmj.com/site/about/guidelines.xhtml

At each DSA, participants were grouped by the type of supplement they consumed:

- non-supplement users (NSU);
- non n-3 PUFA supplement users (SU-n3): participants who consumed one or more supplements that did not contain n-3 PUFA;
- n-3 PUFA supplement users (SU+n3): participants who used n-3 PUFA supplements, either singly or in combination with other non n-3 PUFA supplements.

Participants were also grouped into five categories identifying change in n-3 PUFA supplement use between two consecutive DSA (Appendix I and Appendix II): 'consistent NSU', 'was SU+n3', 'became SU+n3', 'consistent SU+n3', 'other SU'.

Average daily nutrient composition from supplements was calculated using the Vitamin and Mineral Supplement (ViMiS) system [33] and added to the average daily food intake to obtain average total nutrient intake. Validation of these data with biomarkers were undertaken [5].

Dietary covariates

The analyses adjust for potential dietary confounders measured at DSA1. Diet was assessed using a 7-day diet diary (7dDD) between 1993-1998 [34]. Data were entered by trained staff into DINER [35] and checked and calculated by nutritionists using DINERMO [36]. The underlying food composition tables were derived from McCance & Widdowson's Composition of Foods and supplements [37]. Missing values for fatty acids were further completed using a recipe calculation system [38,39]. The baseline 7dDD provided data on average daily energy intake (MJ/d), proportion of energy provided by macronutrients (%En), the sum of EPA and DHA intake (g/d) referred to as 'n-3 PUFA' and consumption of the disaggregated amounts of fruit, vegetable, red meat, processed meat, white meat, and white and oily fish consumption (g/d) [36].

Assessment of other covariates

Social class was measured through occupational status (professional, managerial, skilled non-manual, skilled manual, semi-skilled, non-skilled). Highest education level achieved was divided in four categories (no qualification, O-level, A-level, degree). Both variables were

For peer review only - http://bmjopen.bmj.com/site/about/guidelines.xhtml

derived from HLQ1 (coinciding with DSA1) and are time-fixed. The remaining covariates are time-dependent and refer to measures obtained at time points corresponding to the DSA (Appendix I). We identified participants at higher risk of CHD using responses to the questions "Has the doctor ever told you that you have [myocardial infarction/diabetes/stroke]?". Participants were classified as a never smoker, former smoker or current smoker. Alcohol consumption was classified as none, >0-14, >14-28, >28 units/wk. Marital status was re-categorised into married (married/living as married) or non-married (widowed, divorced, separated, single). Participants were classified as being active, moderately active, moderately inactive or inactive. BMI (kg/m^2) was calculated from height and weight measured by a trained nurse.

Participant selection, case ascertainment and outcomes studied

Participants were eligible for analyses if they provided data on supplement use at any of the three time points (DSA1, DSA2, DSA3) and attended the corresponding HE (Figure 1); 4030 participants were not included at any time point studied. The participant's National Health Services number (a unique national patient identifier) was linked to the data from the Office of National Statistics to obtain vital status and causes of death. A similar procedure was followed for causes of hospital admissions registered by the East-Norfolk Primary Health Care Trust which records for its residents the admissions in England and Wales. The main endpoint studied was CHD mortality mentioned anywhere on the death certificate, identified with ICD9 410-414 or ICD10 I20-I25. In sensitivity analyses, we investigated the use of different definitions for the CHD endpoint: (i) CHD mortality mentioned as underlying cause of death, (ii) death due to acute myocardial infarction (ICD-codes 410 or I21), (iii) first recorded hospitalisation due to CHD.

Statistical analysis

A description of the cohort at DSA1, DSA2 and DSA3 is given. To study potential changes in supplement user characteristics over time, a multinomial logistic regression analysis was performed at each DSA with supplement use group as the outcome variables and the variables noted above as explanatory variables.

For peer review only - http://bmjopen.bmj.com/site/about/guidelines.xhtml

The main analyses used Cox regression, using the follow-up time scale. Individuals who died from causes other than the event of interest were censored at their date of death and individuals who did not die from any cause were censored at the end of follow-up (31st March 2015).

First, we modelled associations between supplement use at DSA1 and CHD mortality, with adjustment for covariates measured at DSA1. A series of *cumulative* adjustment models were used: sex and age-adjusted estimates (model 1); including smoking, BMI (kg/m^2), physical activity, alcohol intake, social class, education and season in which the questionnaire was completed (model 2); including self-reported myocardial infarction, stroke or diabetes (model 3); including energy intake (MJ/d) and disaggregated fruit (g/d), vegetable (g/d), red meat & processed meat (g/d), and white meat (g/d) (model 4); including white fish (g/d) and oily fish (g/d) (model 5). Secondly, models 1-5 were fitted using quintiles of n-3 PUFA intake from food and supplements at DSA1 as the main exposure (Appendix V).

The above analyses were repeated using time-updated measures of supplement use and covariates using model 3. First, we performed separate analyses using DSA1, DSA2 and DSA3 as the time origins. Secondly, we performed time-varying covariates modelling using the full length of follow-up as well as shorter length of follow-up time to acknowledge unobserved changes in individuals' supplement use over time. We equally applied the strategy described here using the *change* between supplement group categories *between* DSA.

We assessed interactions between supplement use and sex, age, self-reported illnesses, smoking and BMI using likelihood ratio tests.

At DSA1, participants with missing data on supplement use and covariables were excluded; at DSA2 and DSA3 missing covariates from the last observation were carried forward (Figure 1). Alternatively, multiple imputation was used to handle missing data in the Cox regression analyses (Appendix VII). The proportional hazards assumption was assessed in the time-varying covariates model, by including interactions between time and each explanatory variable. Data were analysed using Stata v14.

For peer review only - http://bmjopen.bmj.com/site/about/guidelines.xhtml

Results

Analyses were based on 22,035 participants who provided data at DSA1, 15,937 participants at DSA2 and 10,120 participants at DSA3 (Figure 1). Median (IQR) of follow-up time was 19 (17, 20) years. During this time, 1562 participants (out of 22,035) died of CHD mentioned *anywhere* on the death certificate.

Description of included participants

Table 1 describes the study participants at each DSA. Included participants had a median age of 59, 65 and 69 years at DSA1, DSA2 and DSA3 respectively and consisted of 45%, 43% and 44% men. We observed a lower smoking prevalence, fewer alcohol consumers and more physical inactivity as time progressed; however, there was still a large range in these behaviours. The proportion of NSU dropped from 61% at DSA1 to 50% at DSA3, with 24%, 31% and 34% using n-3 PUFA containing supplements at the three DSA respectively. The change in supplement use over time is depicted in Appendix II.

Multinomial logistic regression analysis assessed the relation of these characteristics to supplement use status, mutually adjusted, at DSA1, DSA2 and DSA3 (Figure 2). Having a non-manual social class and a higher education level were associated with SU-n3; whereas older participants, non-smoking, alcohol consumption and fewer self-reported illnesses were associated with SU+n3. Associations between covariates and supplement use group were similar at the three DSA.

n-3 PUFA supplement users and hazard of CHD mortality

There was no evidence that the CHD mortality of SU+n3 or SU-n3 differed from NSU when solely using baseline data (Appendix IV). Table 2 shows results from the Cox proportional hazards analyses in which the time origin was re-set at each DSA. No association was observed between supplement use at DSA1 and CHD mortality, for any amount of follow-up time. On the contrary, the HR of CHD mortality for SU+n3 relative to NSU at DSA2 and DSA3 was significantly below 1. With increasing follow-up time the HR attenuated, but remained statistically significant. We observed stronger associations, for all lengths of follow-up studied, between SU+n3 and CHD mortality in the later DSAs.

For peer review only - http://bmjopen.bmj.com/site/about/guidelines.xhtml

Using time-varying analysis, we observed a 26% lower hazard among SU+n3 compared to NSU (HR: 0.74, 95%CI: 0.66, 0.84). Significant interactions between follow-up time and age, sex and self-reported myocardial infarction were observed; however, inclusion of these interaction terms resulted in negligible differences in the HR or 95%CI of the main exposure. Results presented are therefore without these interaction terms in the regression equation.

Change in supplement use over time

Table 3 shows the results from analysis investigating changes in supplement use over time. There was no evidence to suggest that participants who *were* taking n-3 PUFA supplements at an earlier point in time, but who stopped its use, had a different hazard of CHD mortality compared to consistent NSU ($HR_{DSA1-DSA2}$ 1.24, 95%CI: 0.94, 1.64; $HR_{DSA2-DSA3}$ 1.20, 95%CI: 0.79, 1.81), therefore suggesting that there are no long term benefits of previous n-3 PUFA supplement use. Compared to consistent NSU, participants who reported n-3 PUFA supplement use at both time points, and participants who *became* a SU+n3 had a lower hazard of CHD mortality. This association was stronger in the time-varying covariates analysis where we observed a HR of 0.69 (95%CI: 0.52, 0.90) among starters of SU+n3 and a HR of 0.78 (95%CI: 0.63, 0.98) among consistent SU+n3. Associations attenuated or became non-significant as follow-up time increased.

Sensitivity analyses

Using different definitions for the endpoint (Figure 3), we observed associations of similar strength for CHD as *underlying* cause of death (HR: 0.78, 95%CI: 0.67, 0.90) and for acute fatal myocardial infarction when compared to CHD mortality mentioned *anywhere* on the death certificate. No associations were observed for first event of hospitalisation due to CHD.

Associations between SU+n3 and CHD mortality were similar for men and women, for overweight and non-overweight participants, for participants with and without self-reported myocardial infarction or diabetes and among smokers and non-smokers (Appendix VI). There was evidence of an interaction between supplement use group and prevalent stroke (P=0.006); although the SU+n3 had similar HRs by prevalent stroke group, SU-n3 had not.

For peer review only - http://bmjopen.bmj.com/site/about/guidelines.xhtml

312 The primary analyses do not distinguish between SU+n3 who do or do not use other supplements. As an additional sensitivity analysis, we adjusted for the use of supplements other than n-3 PUFA supplements (categorised as 0, 1, and 2 or more other supplements), finding that the association between n-3 PUFA supplement use and a reduced hazard persisted in those who used other supplements and those who did not. This resulted in an estimated HR of 0.83 (95%CI: 0.71, 0.96) in those who only use an n-3 PUFA containing supplement *vs.* NSU, and a HR of 0.74 (95%CI: 0.60, 0.91) for those who use an n-3 PUFA containing supplement plus one or more other supplements *vs.* NSU. There was no evidence that the reduced hazard in SU+n3 vs. NSU was different in those taking no other supplements (i.e. NSU) or in those taking other supplements.

Discussion

Statement of principal findings

We observed a protective association between n-3 PUFA supplement use and CHD mortality in this general population where fish consumption was low and the mean age was approximately 60 years at baseline (DSA1). When data from three DSA were used, lower hazards of CHD mortality were observed for SU+n3 at all three DSA, which were strongest when the follow-up time was short; however, this analysis lacked statistical power. Using a time-varying covariates analysis, we observed a 26% lower hazard of CHD mortality among SU+n3 compared to NSU.

Strengths and weaknesses of the study

A strength is the selection of participants at baseline from a general population as opposed to fish oil trials which have consisted of high risk populations [11]. Measures of covariates and supplement use were available from three time points, which enabled mitigation of potential bias due to changes in supplement use over time. We could adjust for several variables representing health behaviours, as well as co-morbidities, to account for potential confounding. The DSA had short recall periods of one week; however, we have previously shown that measures obtained from three instruments at baseline (DSA1), with recall times varying from 1 year to 1 week, had good agreement [40]. Supplement use in general is associated with health behaviours [41] and heterogeneity across supplement users exists,

For peer review only

we therefore separated participants into SU+n3 and SU-n3 and carefully assessed potential confounders at all DSA. A further strength was our use of multiple imputation to handle missing data (Appendix VII).

The EPIC-Norfolk study has limitations. The baseline response rate was 39%; however, participants were found to be representative of the Health Survey for England population regarding anthropometry, blood pressure and blood lipids [30]. There was loss to follow-up; however, outcomes were ascertained for those who no longer actively participated. Since the exact dates of supplement use were not known as were the exact dates of self-reported myocardial infarction, stroke or diabetes, we are unable to say which happened first and therefore the chronological ordering is unknown. Healthier individuals might have been more likely to continue active participation; however, we adjusted for a number of factors to mitigate potential bias arising due to this. The time intervals between DSA were relatively long, particularly between DSA1 and DSA2; therefore, misclassification of the exposure is likely to have occurred. Data on fish consumption (and hence n-3 PUFA intake from food) from 7dDD could only be estimated at baseline (Appendix V). We considered the observed association between the SU+n3 and CHD mortality unlikely to be due to the nutrient/compound content of other non n-3 PUFA supplements being used, and indeed in sensitivity analyses we found a significant association between n-3 PUFA supplement use and a reduced hazard in those using no other supplements. We also considered it unlikely that the observed associations could be due to the additional vitamins found in n-3 PUFA containing supplements, since these typically contain a 100% of the recommended nutrient intake and such doses have not been associated with cardiovascular mortality [42,43]. Although we found no association between SU-n3 and a reduced hazard for CHD mortality, we were unable to study the wide variety of individual nutrients/compounds in these supplements and we cannot rule out from these results that such supplements could be associated with CHD mortality. Identification of a specific nutrient and biological pathway, as well as the potential for a relative change in the type of supplement consumed among the SU-n3, requires additional analysis with emphasis on confounding by lifestyle and other factors, possibly particularly so if more than one non n-3 PUFA supplement was consumed. Residual confounding due to dietary differences between SU+n3, SU-n3 and NSU, particularly those occurring over time, such as differences in fruit, vegetables, and meat,

For peer review only - http://bmjopen.bmj.com/site/about/guidelines.xhtml

also cannot be excluded. Lastly, the analyses required interpretation of many models, which will have increased the role of chance findings; however, the significant findings observed may be explained by biological mechanisms, the results were specific to SU+n3 and we observed significant associations of starting or continued use of n-3 PUFA supplements with a reduced hazard for CHD mortality.

Meaning of the study: possible explanations and implications for clinicians and policymakers

The associations observed at the low dose of supplemental EPA and DHA in this cohort makes an antiarrhythmic function of n-3 PUFA a likely biological pathway [6,7]. In time-varying covariate analysis, it was observed that n-3 PUFA supplement use was associated with 26% lower hazard of CHD mortality compared to non-supplement use. A similar association has been estimated from trial and prospective cohort data [6] which concluded that 250-500 mg/d of n-3 PUFA lowered risk by 25% or more; a dose which corresponds to the intake from food and supplements of 300 mg/d observed in this cohort among SU+n3 (Table 1). We have previously reported a non-significant association between plasma n-3 PUFA and CHD incidence in a sub-cohort of the EPIC-Norfolk study [44]; however, plasma concentrations were only available at baseline and are likely to have fluctuated over time, partly considering the variability in supplement use (Appendix II). The results between the studies are consistent when only relying on supplement data from DSA1 in the current analysis (Appendix IV). The VITAL cohort study found no significant association between increasing quartiles of n-3 PUFA intake from food and supplements and CHD (P_{trend}=0.120), although a significant trend was observed for those participants free of baseline CHD (P_{trend}=0.029). When including all DSA, effect modification for prevalent myocardial infarction was not observed in EPIC-Norfolk, which might be indicative of the different motivations for CLO use in the UK *vs.* fish oil supplement use in the US [45,46]. In Europe, the highest oily fish consumption is observed in Spain and Scandinavian countries, and is approximately 2-3 fold higher than fish intake in the UK [47]. A Norwegian cohort, where 30-41% consumed two or more fish meals per week, observed no association between CLO consumption and CHD mortality [19]. Fish could therefore have exceeded the threshold for an antiarrhythmic effect in this Norwegian cohort to which CLO could not add, but might supply a considerable contribution to n-3 PUFA intake in the UK. The AGES study in Iceland,

For peer review only - http://bmjopen.bmj.com/site/about/guidelines.xhtml

402 where the majority of women consumed 2-4 fish portion per week (85% lean fish), did observe a lower risk of hospitalisation due to CHD when n-3 PUFA from cod liver oil was >=5.9 g/week, whereas no association was observed with fish consumption [18]. We did not observe any association with CHD hospitalisation; however, the n-3 PUFA dose in the AGES study was much higher and therefore efficacy might have been through atherosclerotic pathways. Fish oil supplements cannot contain all the nutrients that would be obtained when consuming fish, or indeed the other meal components consumed with it (*e.g.* vegetables) or the red meat replacement that took place [48,49], but n-3 PUFA supplements contain lower concentrations of contamination [50], and provide essential fatty acids without any coating and/or frying oils which might supply trans-fatty acids which are positively associated with CHD [11]. This is consistent with findings that broiled or baked fish have been associated with a lower risk of CHD mortality, whereas fried fish has not [51]. Moreover, from DSA1, it is known that n-3 PUFA supplements were consumed on a daily basis by approximately 95% of the participants [40]. Fish consumption has not been a daily dietary habit in the UK based on National Survey data in 2000/'01 and 2008/'09-2011/'12 [4,52]. Incorporation of n-3 PUFA into plasma, platelets and red blood cells has been shown to be enhanced with a daily *vs.* twice weekly regime of similar weekly supplement doses [53].

420 ## Unanswered questions and future research

421 Food composition tables which include contaminants (Appendix V), nutrient composition from supplements and foods and which distinguish between fish preparation methods, could clarify any association between n-3 PUFA sources and CHD to further inform public health dietary guidelines. The results from the first primary prevention trial on n-3 PUFA supplement use are to be expected [29].

Conclusion

426 Recent use of n-3 PUFA supplements was associated with a lower hazard of CHD mortality in a population-based cohort with low fish consumption. This association was specific to SU+n3 and independent of history of myocardial infarction.

For peer review only - http://bmjopen.bmj.com/site/about/guidelines.xhtml

References

1 Dyerberg J. Fatty acid composition of the plasma lipids in hypothyroid subjects. *Acta Med Scand* 1968;**184**:441–9.

2 Kromhout D, Bosschieter EB, de Lezenne Coulander C. The inverse relation between fish consumption and 20-year mortality from coronary heart disease. *N Engl J Med* 1985;**312**:1205–9.

3 Zheng J, Huang T, Yu Y, *et al.* Fish consumption and CHD mortality: an updated meta-analysis of seventeen cohort studies. *Public Health Nutr* 2012;**15**:725–37.

4 Henderson L, Gregory J, Swan G. The National Diet & Nutrition Survey: adults aged 19 to 64 years. Types and quantities of foods consumed. London: : Her Majesty's Stationery Office (HMSO) 2002.

5 Lentjes MAH, Mulligan AA, Welch AA, *et al.* Contribution of cod liver oil-related nutrients (vitamins A, D, E and eicosapentaenoic acid and docosahexaenoic acid) to daily nutrient intake and their associations with plasma concentrations in the EPIC-Norfolk cohort. *J Hum Nutr Diet* 2015;**28**:568–82. doi:10.1111/jhn.12271

6 Mozaffarian D, Rimm EB. Fish intake, contaminants, and human health: evaluating the risks and the benefits. *JAMA* 2006;**296**:1885–99.

7 London B, Albert C, Anderson ME, *et al.* Omega-3 Fatty Acids and Cardiac Arrhythmias: Prior Studies and Recommendations for Future Research. *Circulation* 2007;**116**:e320–35.

8 Chiuve SE, Rimm EB, Sandhu RK, *et al.* Dietary fat quality and risk of sudden cardiac death in women. *Am J Clin Nutr* 2012;**96**:498–507.

9 Hooper L, Thompson RL, Harrison R a, *et al.* Risks and benefits of omega 3 fats for mortality, cardiovascular disease, and cancer: systematic review. *BMJ* 2006;**332**:752–60.

10 Rizos EC, Ntzani EE, Bika E, *et al.* Association between omega-3 fatty acid supplementation and risk of major cardiovascular disease events: a systematic review and meta-analysis. *JAMA* 2012;**308**:1024–33.

11 Chowdhury R, Warnakula S, Kunutsor S, *et al.* Association of dietary, circulating, and supplement fatty acids with coronary risk: A systematic review and meta-analysis. *Ann Intern Med* 2014;**160**:398–406.

12 Mozaffarian D, Wu JHY. Omega-3 fatty acids and cardiovascular disease: effects on risk factors, molecular pathways, and clinical events. *J Am Coll Cardiol* 2011;**58**:2047–67.

13 Wu JHY, Mozaffarian D. ω-3 Fatty acids, atherosclerosis progression and cardiovascular outcomes in recent trials: new pieces in a complex puzzle. *Heart* 2014;**100**:530–3.

14 James MJ, Sullivan TR, Metcalf RG, *et al.* Pitfalls in the use of randomised controlled trials for fish oil studies with cardiac patients. *Br J Nutr* 2014;**112**:812–20.

For peer review only

For peer review only - http://bmjopen.bmj.com/site/about/guidelines.xhtml

15 Ridker PM. Fish consumption, fish oils, and cardiovascular events: still waiting for definitive evidence. *Am J Clin Nutr* 2016;:2–3.

16 Skeie G, Braaten T, Hjartåker A, *et al.* Use of dietary supplements in the European Prospective Investigation into Cancer and Nutrition calibration study. *Eur J Clin Nutr* 2009;**63**:S226–38.

17 Messerer M, Håkansson N, Wolk A, *et al.* Dietary supplement use and mortality in a cohort of Swedish men. *Br J Nutr* 2008;**99**:626–31.

18 Haraldsdottir A, Torfadottir JE, Valdimarsdottir UA, *et al.* Fish and fish-liver oil consumption in adolescence and midlife and risk of CHD in older women. *Public Health Nutr* 2016;**19**:318–25.

19 Egeland GM, Meyer HE, Selmer R, *et al.* Cod liver oil consumption, smoking, and coronary heart disease mortality: three counties, Norway. *Int J Circumpolar Health* 2001;**60**:143–9.

20 Mozaffarian D, Lemaitre RN, King IB, *et al.* Plasma phospholipid long-chain omega-3 fatty acids and total and cause-specific mortality in older adults, a cohort study. *Ann Intern Med* 2013;**158**:515–25.

21 Bell GA, Kantor ED, Lampe JW, *et al.* Intake of Long-Chain ω-3 Fatty Acids From Diet and Supplements in Relation to Mortality. *Am J Epidemiol* 2014;**179**:710–20.

22 Yokoyama M, Origasa H, Matsuzaki M, *et al.* Effects of eicosapentaenoic acid on major coronary events in hypercholesterolaemic patients (JELIS): a randomised open-label, blinded endpoint analysis. *Lancet* 2007;**369**:1090–8.

23 The Origin Trial Investigators. Basal Insulin and Cardiovascular and Other Outcomes in Dysglycemia. *N Engl J Med* 2012;**367**:319–28.

24 Risk and Prevention Study Collaborative Group, Roncaglioni MC, Tombesi M, *et al.* n-3 fatty acids in patients with multiple cardiovascular risk factors. *N Engl J Med* 2013;**368**:1800–8.

25 Burr ML. Secondary prevention of CHD in UK men: the Diet and Reinfarction Trial and its sequel. *Proc Nutr Soc* 2007;**66**:9–15.

26 GISSI-Prevenzione Investigators. Dietary supplementation with n-3 polyunsaturated fatty acids and vitamin E after myocardial infarction: results of the GISSI-Prevenzione trial. Gruppo Italiano per lo Studio della Sopravvivenza nell'Infarto miocardico. *Lancet* 1999;**354**:447–55.

27 Rauch B, Schiele R, Schneider S, *et al.* OMEGA, a Randomized, Placebo-Controlled Trial to Test the Effect of Highly Purified Omega-3 Fatty Acids on Top of Modern Guideline-Adjusted Therapy After Myocardial Infarction. *Circulation* 2010;**122**:2152–9.

28 Galan P, Kesse-Guyot E, Czernichow S, *et al.* Effects of B vitamins and omega 3 fatty acids on cardiovascular diseases: a randomised placebo controlled trial. *BMJ* 2010;**341**:c6273.

29 Manson JE, Bassuk SS, Lee I-M, *et al.* The VITamin D and OmegA-3 TriaL (VITAL):

For peer review only - http://bmjopen.bmj.com/site/about/guidelines.xhtml

Rationale and design of a large randomized controlled trial of vitamin D and marine omega-3 fatty acid supplements for the primary prevention of cancer and cardiovascular disease. Contemp. Clin. Trials. 2012;**33**:159–71.

30 Day N, Oakes S, Luben R, *et al.* EPIC-Norfolk: study design and characteristics of the cohort. European Prospective Investigation of Cancer. *Br J Cancer* 1999;**80 Suppl 1**:95–103.

31 Hayat SA, Luben R, Keevil VL, *et al.* Cohort profile: A prospective cohort study of objective physical and cognitive capability and visual health in an ageing population of men and women in Norfolk (EPIC-Norfolk 3). *Int J Epidemiol* 2014;**43**:1063–72.

32 European Commission. Directive 2002/46/EC of the European Parliament and of the Council of 10 June 2002 on the approximation of the laws of the Member States relating to food supplements. *Off J Eur Communities* 2002;**L183/51**.

33 Lentjes MAH, Bhaniani A, Mulligan A a, *et al.* Developing a database of vitamin and mineral supplements (ViMiS) for the Norfolk arm of the European Prospective Investigation into Cancer (EPIC-Norfolk). *Public Health Nutr* 2011;**14**:459–71.

34 Bingham SA, Welch AA, McTaggart A, *et al.* Nutritional methods in the European Prospective Investigation of Cancer in Norfolk. *Public Health Nutr* 2001;**4**:847–58.

35 Welch AA, McTaggart A, Mulligan AA, *et al.* DINER (Data Into Nutrients for Epidemiological Research) - a new data-entry program for nutritional analysis in the EPIC-Norfolk cohort and the 7-day diary method. *Public Heal Nutr* 2001;**4**:1253–65.

36 Lentjes MAH, McTaggart A, Mulligan AA, *et al.* Dietary intake measurement using 7 d diet diaries in British men and women in the European Prospective Investigation into Cancer-Norfolk study: a focus on methodological issues. *Br J Nutr* 2014;**111**:516–26.

37 Holland B, Welch AA, Unwin D, *et al. McCance and Widdowson's The Composition of Foods*. Cambridge: : Royal Society of Chemistry (RSC) 1991.

38 Mulligan AA, Kuhnle GGC, Lentjes MAH, *et al.* Intakes and sources of isoflavones, lignans, enterolignans, coumestrol and soya-containing foods in the Norfolk arm of the European Prospective Investigation into Cancer and Nutrition (EPIC-Norfolk), from 7 d food diaries, using a newly updated database. *Public Health Nutr* 2013;**16**:1454–62.

39 Welch AAA, Shakya-Shrestha S, Lentjes MAH, *et al.* Dietary intake and status of n-3 polyunsaturated fatty acids in a population of fish-eating and non-fish-eating meat-eaters, vegetarians, and vegans and the product-precursor ratio [corrected] of α-linolenic acid to long-chain n-3 polyunsaturated fatty ac. *Am J Clin Nutr* 2010;**92**:1040–51.

40 Lentjes MAH, Welch AA, Luben RN, *et al.* Differences in dietary supplement use and secular and seasonal trends assessed using three different instruments in the EPIC-Norfolk population study. *J Diet Suppl* 2013;**10**:142–51.

41 Lentjes MAH, Welch AA, Mulligan AA, *et al.* Cod liver oil supplement consumption and health: cross-sectional results from the EPIC-Norfolk cohort study. *Nutrients* 2014;**6**:4320–37.

For peer review only

For peer review only - http://bmjopen.bmj.com/site/about/guidelines.xhtml

548 42 Macpherson H, Pipingas A, Pase MP. Multivitamin-multimineral supplementation and mortality: a meta-analysis of randomized controlled trials. *Am J Clin Nutr* 2013;**97**:437–44.

551 43 Myung S-K, Ju W, Cho B, *et al.* Efficacy of vitamin and antioxidant supplements in prevention of cardiovascular disease: systematic review and meta-analysis of randomised controlled trials. *BMJ* 2013;**346**:f10.

554 44 Khaw K-T, Friesen MD, Riboli E, *et al.* Plasma phospholipid fatty acid concentration and incident coronary heart disease in men and women: the EPIC-Norfolk prospective study. *PLoS Med* 2012;**9**:e1001255.

557 45 Kim HJ, Giovannucci E, Rosner B, *et al.* Longitudinal and secular trends in dietary supplement use: Nurses' health study and health professionals follow-up study, 1986-2006. *J Acad Nutr Diet* 2014;**114**:436–43.

560 46 Rajakumar K. Vitamin D, cod-liver oil, sunlight, and rickets: a historical perspective. *Pediatrics* 2003;**112**:e132-5.

562 47 Welch A, Lund E, Amiano P, *et al.* Variability of fish consumption within the 10 European countries participating in the European Investigation into Cancer and Nutrition (EPIC) study. *Public Health Nutr* 2002;**5**:1273–85.

565 48 Kiefte-de Jong JC, Chowdhury R, Franco OH. Fish intake or omega-3 fatty acids: greater than the sum of all parts? *Eur J Epidemiol* 2012;**27**:891–4.

567 49 He K. Fish, Long-Chain Omega-3 Polyunsaturated Fatty Acids and Prevention of Cardiovascular Disease-Eat Fish or Take Fish Oil Supplement? *Prog Cardiovasc Dis* 2009;**52**:95–114.

570 50 Bourdon JA, Bazinet TM, Arnason TT, *et al.* Polychlorinated biphenyls (PCBs) contamination and aryl hydrocarbon receptor (AhR) agonist activity of Omega-3 polyunsaturated fatty acid supplements: implications for daily intake of dioxins and PCBs. *Food Chem Toxicol* 2010;**48**:3093–7.

574 51 Mozaffarian D, Lemaitre RN, Kuller LH, *et al.* Cardiac benefits of fish consumption may depend on the type of fish meal consumed: The cardiovascular health study. *Circulation* 2003;**107**:1372–7.

577 52 Bates B, Lennox A, Prentice A, *et al.*, editors. *National Diet and Nutrition Survey Results from Years 1 , 2 , 3 and 4 (combined) of the Rolling Programme (2008/2009-2011/2012)*. London: : Public Health England 2014.

580 53 Browning LM, Walker CG, Mander AP, *et al.* Compared with daily, weekly n-3 PUFA intake affects the incorporation of eicosapentaenoic acid and docosahexaenoic acid into platelets and mononuclear cells in humans. *J Nutr* 2014;**144**:667–72.

583

For peer review only - http://bmjopen.bmj.com/site/about/guidelines.xhtml

For peer review only

Tables

Table 1: Characteristics of the EPIC-Norfolk cohort at time of DSA1 (1993-1998), DSA2 (2002-2004) and DSA3 (2004-2011). Values are % (n) for categorical variables and median (IQR) for continuous variables.

	DSA1 N=22,035	DSA2 N=15,937	DSA3 N=10,120
CHD mortality events from DSA onwards (n)	1562	742	241
Total person years at risk (years)	383,444	173,239	63,535
Crude cumulative rate (per 1000 person years)	4.074	4.283	3.793
Supplement use			
NSU	61 (13444)	52 (8353)	50 (5029)
SU-n3	15 (3263)	17 (2665)	16 (1610)
SU+n3	24 (5328)	31 (4919)	34 (3481)
n-3 PUFA (g/d)*			
From food only	0.12 (0.06, 0.34)	-	-
From food and supplements combined	0.16 (0.07, 0.40)	-	-
among SU+n3 only	0.30 (0.17, 0.72)	-	-
Sex			
Men	45 (9890)	43 (6923)	44 (4418)
Women	55 (12,145)	57 (9014)	56 (5702)
Age (years)	59 (51, 67)	65 (58, 72)	69 (63, 76)
BMI (kg/m^2)	25.8 (23.7, 28.3)	26.1 (24.0, 28.7)	26.3 (24.0, 29.0)
Smoking status			
Current	11 (2395)	7 (1149)	5 (456)
Former	43 (9426)	50 (7982)	46 (4671)
Never	46 (10,214)	43 (6806)	49 (4993)
Social class			
Professional	7 (1531)	8 (1203)	8 (852)
Managerial	37 (8048)	39 (6189)	40 (4047)
Skilled non-manual	17 (3715)	16 (2585)	16 (1658)
Skilled manual	23 (5055)	22 (3483)	21 (2131)
Semi-skilled	13 (2910)	13 (2007)	12 (1163)
Non-skilled	4 (776)	3 (470)	3 (269)
Marital status			
Married	82 (18,127)	78 (12394)	76 (7708)
Not married	18 (3908)	22 (3543)	24 (2412)
Education level			
No qualification	36 (7999)	32 (5106)	28 (2851)
O-level	10 (2282)	11 (1757)	12 (1184)
A-level	41 (8955)	42 (6720)	44 (4427)
Degree	13 (2799)	15 (2354)	16 (1658)
Season of 7dDD			
Spring	26 (5817)	23 (3645)	27 (2761)
Summer	25 (5473)	21 (3295)	30 (3008)
Autumn	26 (5661)	32 (5150)	25 (2513)
Winter	23 (5084)	24 (3847)	18 (1838)

For peer review only

	DSA1 N=22,035	DSA2 N=15,937	DSA3 N=10,120
Physical activity			
Inactive	30 (6592)	36 (5809)	41 (4106)
Moderately inactive	29 (6389)	27 (4302)	28 (2792)
Moderately active	23 (5040)	20 (3151)	17 (1709)
Active	18 (4014)	17 (2675)	15 (1513)
Alcohol intake (HLQ)			
None	13 (2930)	32 (5042)	33 (3312)
>0-14 units/wk	72 (15,880)	52 (8336)	56 (5704)
>14-28 units/wk	11 (2418)	12 (1950)	9 (869)
>28 units/wk	4 (807)	4 (609)	2 (235)
Self-reported illness			
Myocardial infarction	3 (704)	5 (717)	3 (330)
Stroke	1 (304)	2 (359)	2 (164)
Diabetes	2 (510)	5 (745)	3 (338)
Energy – 7dDD (MJ/d)*	8.00 (6.71, 9.56)	-	-
Protein (en%)	15.2 (13.6, 16.9)	-	-
Fat (en%)	33.4 (29.8, 36.8)	-	-
Saturated fat (en%)	12.7 (10.9, 14.6)	-	-
Carbohydrates (en%)	47.6 (43.4, 51.5)	-	-
Alcohol (en%)	2.0 (0, 5.9)	-	-
Food intake – 7dDD (g/d)*			
Fruit	153 (82, 242)	-	-
Vegetables	142 (102, 190)	-	-
Red & processed meat	53 (32, 76)	-	-
White meat	21 (6, 37)	-	-
Oily fish	4 (0, 19)	-	-
Consumers only (54%)	17 (10, 30)	-	-
White fish	13 (0, 23)	-	-
Consumers only (66%)	19 (13, 29)	-	-
Total fish	23 (11, 40)	-	-
Consumers only (84%)	28 (17, 44)	-	-

7dDD, 7-day diet diary; HLQ, health and lifestyle questionnaire; DSA, dietary supplement assessment; BMI, body mass index; n-3 PUFA, omega-3 polyunsaturated fatty acids (sum of eicosapentaenoic acid and docosahexaenoic acid); SU, supplement user; NSU, non-supplement user.
*Data only available from DSA1, see also Appendix I; for more information on diet at DSA1 see Appendix III.

For peer review only

Table 2: The association between supplement use reported at DSA1, DSA2 and DSA3 and subsequent hazard of CHD mortality (where cause of death was mentioned anywhere on the death certificate) adjusted using model 3*. The follow-up time in the EPIC-Norfolk study was from 1993 to 2015. The analysis was performed in two ways.**

		**Two years of follow-up**		**Four years of follow-up**		**Follow-up time until next DSA*****		**Full follow-up time**	
	Total N	CHD Events N	HR (95% CI)	CHD Events N	HR (95% CI)	CHD Events N	HR (95% CI)	CHD Events N	HR (95% CI)
DSA1 (1993-1998)	22,035	71		174		872		1562	
NSU	13,444	47	1.00	113	1.00	580	1.00	1012	1.00
SU-n3	3263	9	1.24 (0.60, 2.56)	20	1.17 (0.73, 1.90)	101	1.06 (0.85, 1.31)	178	0.96 (0.82, 1.13)
SU+n3	5328	15	0.95 (0.53, 1.72)	41	1.05 (0.73, 1.52)	191	0.86 (0.73, 1.02)	372	0.94 (0.83, 1.06)
DSA2 (2002-2004)	15,937	75		171		527		742	
NSU	8353	53	1.00	115	1.00	346	1.00	475	1.00
SU-n3	2665	10	1.01 (0.51, 2.02)	25	1.24 (0.79, 1.93)	61	0.94 (0.71, 1.24)	81	0.84 (0.66, 1.07)
SU+n3	4919	12	0.55 (0.29, 1.04)	31	0.64 (0.43, 0.96)	120	0.75 (0.61, 0.93)	186	0.81 (0.68, 0.97)
DSA3 (2004-2011)	10,120	59		125		241			
NSU	5029	44	1.00	82	1.00	148	1.00		
SU-n3	1610	6	0.60 (0.25, 1.42)	19	1.01 (0.61, 1.67)	35	1.00 (0.69, 1.45)		
SU+n3	3481	9	0.38 (0.18, 0.78)	24	0.52 (0.33, 0.83)	58	0.70 (0.52, 0.95)		
Time-varying	24,330	205		470		1640			
NSU		144	1.00	310	1.00	1074	1.00		
SU-n3		25	0.93 (0.61, 1.43)	64	1.13 (0.86, 1.49)	197	0.91 (0.78, 1.06)		
SU+n3		36	0.59 (0.41, 0.85)	96	0.73 (0.58, 0.92)	369	0.74 (0.66, 0.84)		

DSA, Dietary Supplement Assessment; HR, hazard ratio; 95%CI, 95% confidence interval; NSU, non-supplement users; SU-n3, non-n-3 PUFA supplement users; SU+n3, n-3 PUFA supplement users (mainly cod liver oil).

* Using adjustment Model 3: time-point specific age, smoking, BMI, alcohol consumption, physical activity, season of questionnaire completion, marital status and self-report of myocardial infarction, stroke or diabetes; as well as: sex, social class and education measured at DSA1.

** We performed the analysis in two ways. First, in separate analyses using DSA1, DSA2 and DSA3 as the time origins, such that a single DSA was used to predict future CHD mortality (top three sections). Secondly, we performed an analysis which combines the three DSA into a single analysis. In more detail, we used the most up-to-date exposure and covariate measures for each individual at each time they were at risk, *i.e.* time-varying covariates modelling. In the 'time-varying' approach, the follow-up time is divided by the dates of the respective DSA measures. Each participant is allocated to an exposure group ('NSU', 'SU-n3' or 'SU+n3'), but only for that section of the follow-up time in which they belonged to that group. If they changed supplement use (i.e. 'varied' by stopping a supplement or changing the type of supplement) the next

For peer review only - http://bmjopen.bmj.com/site/about/guidelines.xhtml

section of the follow-up time (until the next DSA) was allocated to the exposure group they changed to. This type of analysis reduces misclassification of the exposure and any other confounders over time in a single analysis. Please note, the variables in this analysis do not explain what the associated risk is when changing from a specific supplement user category to another, this is shown in Table 3. The re-shaped dataset for time-varying analyses contains a larger number of participants (N=24,330) than available at DSA1 alone since 2295 participants did not complete DSA1, but did complete DSA2 and/or DSA3; equally, some participants were excluded from DSA1 due to missing covariate data when these covariates were available at DSA2 and/or DSA3 and so the participant contributed follow-up time from DSA2 and/or DSA3 only.
*** or in case of DSA3, the censor date was the date of administrative follow-up (31 March 2015).

For peer review only - http://bmjopen.bmj.com/site/about/guidelines.xhtml

Table 3: The association between change in supplement use and subsequent hazard of CHD mortality (where cause of death was mentioned anywhere on the death certificate). The follow-up time in the EPIC-Norfolk study was from 2002 for DSA1-DSA2 and from 2004 for DSA2-DSA3 (until 2015).

		Two years of follow-up		Four years of follow-up		Follow-up time until next DSA*		Full follow-up time	
	Total N	Events N	HR (95% CI)	Events N	HR (95% CI)	Events N	HR (95% CI)	Events N	HR (95% CI)
Change between DSA1-DSA2	14,283	71		162		477		672	
Consistent NSU	5861	42	1.00	89	1.00	240	1.00	335	1.00
Were SU+n3	1297	9	1.03 (0.50, 2.12)	21	1.13 (0.70, 1.82)	64	1.24 (0.94, 1.64)	81	1.12 (0.88, 1.43)
Became SU+n3	2318	6	0.58 (0.25, 1.38)	18	0.79 (0.47, 1.31)	46	0.69 (0.50, 0.94)	75	0.77 (0.60, 1.00)
Consistent SU+n3	2213	6	0.51 (0.22, 1.21)	13	0.50 (0.28, 0.90)	65	0.87 (0.66, 1.15)	96	0.88 (0.70, 1.11)
Other type of SU	2594	8	0.71 (0.33, 1.53)	21	0.90 (0.56, 1.46)	62	0.93 (0.70, 1.24)	85	0.86 (0.67, 1.09)
Change between DSA2-DSA3	9221	58		118		218			
Consistent NSU	3427	37	1.00	59	1.00	107	1.00		
Were SU+n3	932	5	0.58 (0.23, 1.49)	17	1.22 (0.71, 2.11)	29	1.20 (0.79, 1.81)		
Became SU+n3	1173	2	0.22 (0.05, 0.94)	8	0.54 (0.25, 1.12)	17	0.63 (0.38, 1.06)		
Consistent SU+n3	2037	7	0.41 (0.18, 0.93)	15	0.54 (0.31, 0.96)	37	0.75 (0.52, 1.10)		
Other type of SU	1652	7	0.57 (0.25, 1.29)	19	0.97 (0.58, 1.65)	28	0.79 (0.52, 1.21)		
*Time-varying***	15,356	129		280		695			
Consistent NSU		79	1.00	148	1.00	347	1.00		
Were SU+n3		14	0.81 (0.46, 1.44)	38	1.18 (0.82, 1.69)	93	1.25 (0.99, 1.57)		
Became SU+n3		8	0.42 (0.20, 0.88)	26	0.70 (0.46, 1.07)	63	0.69 (0.52, 0.90)		
Consistent SU+n3		13	0.46 (0.26, 0.84)	28	0.53 (0.35, 0.80)	102	0.78 (0.63, 0.98)		
Other type of SU		15	0.64 (0.37, 1.13)	40	0.92 (0.65, 1.32)	90	0.85 (0.68, 1.08)		

DSA, Dietary Supplement Assessment; HR, hazard ratio; 95%CI, 95% confidence interval; NSU, non-supplement users; SU-n3, non-n-3 PUFA supplement users; SU+n3, n-3 PUFA supplement users (mainly cod liver oil).

Using adjustment Model 3: covariates are from the latest considered time-point (*e.g.* DSA2, when analysing change between DSA1 and DSA2): age, smoking, BMI, alcohol consumption, physical activity, season of questionnaire completion, marital status and self-report of myocardial infarction, stroke or diabetes; as well as: sex, social class and education measured at DSA1.

* Or in case of DSA3, the censor date was the date of administrative follow-up (31 March 2015).

** The re-shaped dataset contains a larger number of participants (N=15,356), since participants did not complete DSA1, but did complete DSA2 and/or DSA3; equally, some participants were excluded from DSA1 due to missing covariate data when these covariates were available at DSA2 and/or DSA3 and so the participant contributed follow-up time from DSA2 and/or DSA3 only.

For peer review only - http://bmjopen.bmj.com/site/about/guidelines.xhtml

Figures

Figure 1: Number of participants included at each DSA time point studied, with different scenarios of exclusion due to missing data.

DSA, dietary supplement assessment; HLQ, health and lifestyle questionnaire; HE, health examination.
* Social class, education, smoking status, physical activity, BMI, marital status and alcohol consumption.
The numbers in the orange and green boxes relate to the number of participants available when the two consecutive DSA were considered in the analysis. The grey area in the timeline represents unobserved time.
For more information regarding data availability see Appendix I.

Figure 2: Characteristics of SU-n3 vs. NSU and SU+n3 vs. NSU measured at three time points (DSA1, DSA2, DSA3) in the EPIC-Norfolk study.

Three analyses from multinomial logistic regression at three time points: **DSA1 (N=22,035)**, **DSA2 (N=12,333)**, **DSA3 (N=8126)**. The ORs are mutually adjusted for all variables shown. DSA, dietary supplement assessment; NSU, non-supplement user; SU+n3, n-3 PUFA supplement user; SU-n3; supplement user without n-3 PUFA.

Figure 3: Time-varying covariate analysis of the association between supplement use and hazard of CHD in the EPIC-Norfolk study (follow-up time from 1993 to 2015).

NSU, non-supplement users; SU-n3, non-N-3 PUFA supplement users; SU+n3, N-3 PUFA supplement users (mainly cod liver oil); MI, myocardial infarction.

(i) CHD mentioned *anywhere* on the death certificate (n=1640/24,330, as Table 2).
(ii) CHD mentioned as *underlying* cause of death on death certificate (n=1084/24,330).
(iii) *Acute myocardial infarction* as underlying cause of death on death certificate (n=411/24,330).
(iv) *Hospitalisation* due to CHD (n=4087/24,217).

The re-shaped dataset for mortality analysis contains a larger number of participants (N=24,330) than available at DSA1 alone and therefore more events, since participants did not complete DSA1, but did complete DSA2 and/or DSA3; equally, some participants were excluded from DSA1 due to missing covariate data whereas these covariates were available at DSA2 and/or DSA3 and so the participant contributed follow-up time from DSA2 and/or DSA3 only.

The re-shaped dataset for hospitalisation analysis contains a smaller number of participants (N=24,216) than the mortality analysis, since participants who did not complete DSA1, but completed DSA2 or DSA3 -however had a non-fatal event before DSA2 or DSA3 respectively- were excluded.

Using adjustment Model 3: time-point specific age, smoking, BMI, alcohol consumption, physical activity, season of questionnaire completion, marital status and self-report of myocardial infarction, stroke or diabetes; as well as: sex, social class and education.

For peer review only

For peer review only - http://bmjopen.bmj.com/site/about/guidelines.xhtml

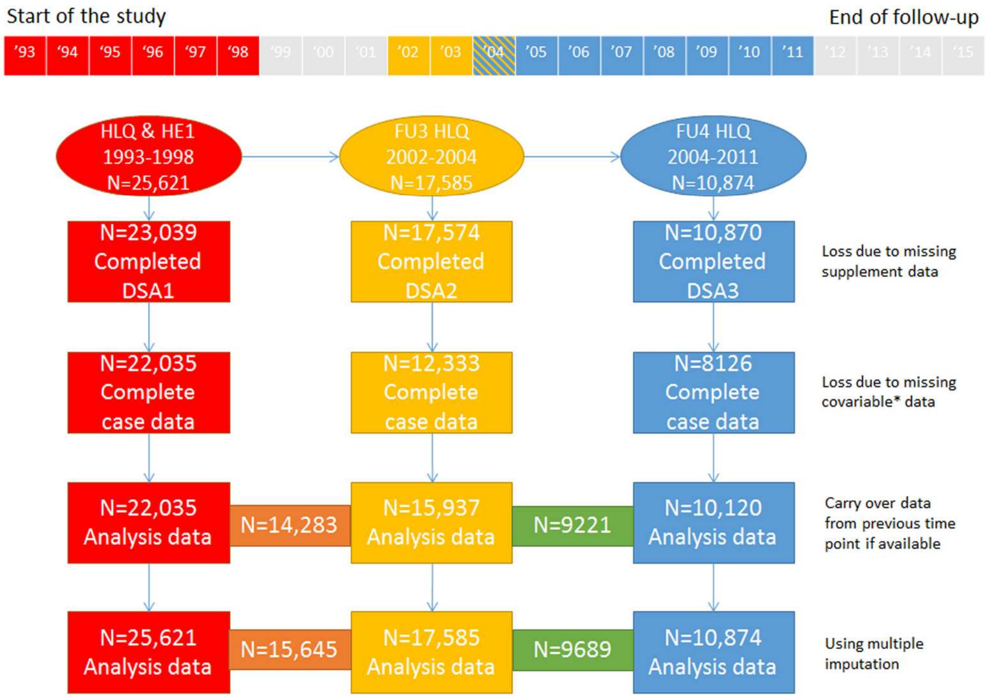

Number of participants included at each DSA time point studied, with different scenarios of exclusion due to missing data.
Footnote: DSA, dietary supplement assessment; HLQ, health and lifestyle questionnaire; HE, health examination.
* Social class, education, smoking status, physical activity, BMI, marital status and alcohol consumption. The numbers in the orange and green boxes relate to the number of participants available when the two consecutive DSA were considered in the analysis. The grey area in the timeline represents unobserved time. For more information regarding data availability see Appendix I

248x174mm (300 x 300 DPI)

only

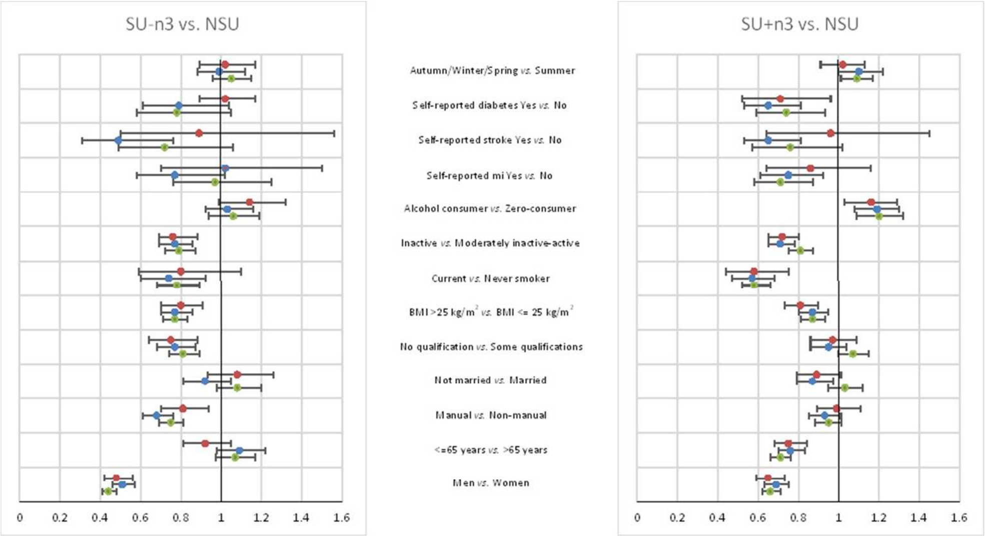

Characteristics of SU-n3 vs. NSU and SU+n3 vs. NSU measured at three time points (DSA1, DSA2, DSA3) in the EPIC-Norfolk study.
Footnote: Three analyses from multinomial logistic regression at three time points: DSA1 (N=22,035), DSA2 (N=12,333), DSA3 (N=8126). The ORs are mutually adjusted for all variables shown. DSA, dietary supplement assessment; NSU, non-supplement user; SU+n3, n3 PUFA supplement user; SU n3; supplement user without n3 PUFA.

83x48mm (300 x 300 DPI)

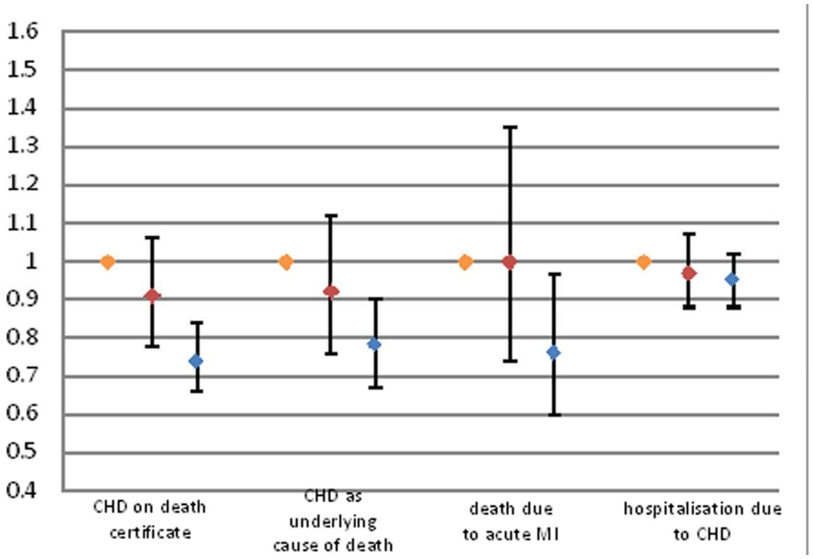

Time-varying covariate analysis of the association between supplement use and hazard of CHD in the EPIC-Norfolk study (follow-up time from 1993 to 2015).
Footnote: NSU, non-supplement users; SU-n3, non-N-3 PUFA supplement users; SU+n3, N-3 PUFA supplement users (mainly cod liver oil); MI, myocardial infarction.

(i) CHD mentioned anywhere on the death certificate (n=1640/24,330, as Table 2).
(ii) CHD mentioned as underlying cause of death on death certificate (n=1084/24,330).
(iii) Acute myocardial infarction as underlying cause of death on death certificate (n=411/24,330).
(iv) Hospitalisation due to CHD (n=4087/24,2176).

The re-shaped dataset for mortality analysis contains a larger number of participants (N=24,330) than available at DSA1 alone and therefore more events, since participants did not complete DSA1, but did complete DSA2 and/or DSA3; equally, some participants were excluded from DSA1 due to missing covariate data whereas these covariates were available at DSA2 and/or DSA3 and so the participant contributed follow-up time from DSA2 and/or DSA3 only.

The re-shaped dataset for hospitalisation analysis contains a smaller number of participants (N=24,216) than the mortality analysis, since participants who did not complete DSA1, but completed DSA2 or DSA3 - however had a non-fatal event before DSA2 or DSA3 respectively- were excluded.

Using adjustment Model 3: time-point specific age, smoking, BMI, alcohol consumption, physical activity, season of questionnaire completion, marital status and self-report of myocardial infarction, stroke or diabetes; as well as: sex, social class and education.

145x80mm (300 x 300 DPI)

Longitudinal associations between marine omega-3 supplement users and Coronary Heart Disease in a population-based cohort

STROBE Statement—checklist of items that should be included in reports of observational studies

Line numbers correspond when using track change.

Line		Item No	Recommendation
1-4	**Title and abstract**	1	(*a*) Indicate the study's design with a commonly used term in the title or the abstract
79-108			(*b*) Provide in the abstract an informative and balanced summary of what was done and what was found
	**Introduction**		
132-154	Background/rationale	2	Explain the scientific background and rationale for the investigation being reported
155-157	Objectives	3	State specific objectives, including any prespecified hypotheses
	**Methods**		
160-168; Appendix I	Study design	4	Present key elements of study design early in the paper
160-168	Setting	5	Describe the setting, locations, and relevant dates, including periods of recruitment, exposure, follow-up, and data collection
215-226	Participants	6	(*a*) *Cohort study*—Give the eligibility criteria, and the sources and methods of selection of participants. Describe methods of follow-up<br>*Case-control study*—Give the eligibility criteria, and the sources and methods of case ascertainment and control selection. Give the rationale for the choice of cases and controls<br>*Cross-sectional study*—Give the eligibility criteria, and the sources and methods of selection of participants
-			(*b*) *Cohort study*—For matched studies, give matching criteria and number of exposed and unexposed<br>*Case-control study*—For matched studies, give matching criteria and the number of controls per case
170-213; Appendix I	Variables	7	Clearly define all outcomes, exposures, predictors, potential confounders, and effect modifiers. Give diagnostic criteria, if applicable
170-213; Appendix I	Data sources/ measurement	8*	For each variable of interest, give sources of data and details of methods of assessment (measurement). Describe comparability of

For peer review only

			assessment methods if there is more than one group
217-221; 257-260	Bias	9	Describe any efforts to address potential sources of bias
Figure 1	Study size	10	Explain how the study size was arrived at
170-213; Appendix I	Quantitative variables	11	Explain how quantitative variables were handled in the analyses. If applicable, describe which groupings were chosen and why
228-260	Statistical methods	12	(*a*) Describe all statistical methods, including those used to control for confounding
228-231; 253-254			(*b*) Describe any methods used to examine subgroups and interactions
257-260; Figure 1; Appendix VII			(*c*) Explain how missing data were addressed
			(*d*) *Cohort study*—If applicable, explain how loss to follow-up was addressed<br>*Case-control study*—If applicable, explain how matching of cases and controls was addressed<br>*Cross-sectional study*—If applicable, describe analytical methods taking account of sampling strategy
			(*e*) Describe any sensitivity analyses
	**Results**		
261-264; Figure 1	Participants	13*	(a) Report numbers of individuals at each stage of study—eg numbers potentially eligible, examined for eligibility, confirmed eligible, included in the study, completing follow-up, and analysed
Appendix I			(b) Give reasons for non-participation at each stage
Figure 1			(c) Consider use of a flow diagram
266-278; Figure 2; Appendix III + V	Descriptive data	14*	(a) Give characteristics of study participants (eg demographic, clinical, social) and information on exposures and potential confounders
Appendix I + VII			(b) Indicate number of participants with missing data for each variable of interest
261-264			(c) *Cohort study*—Summarise follow-up time (eg, average and total amount)
261-264	Outcome data	15*	*Cohort study*—Report numbers of outcome events or summary measures over time
			Case-control study—Report numbers in each exposure category, or summary measures of exposure
			Cross-sectional study—Report numbers of outcome events or summary measures
Tables 2-3; Appendix V;	Main results	16	(*a*) Give unadjusted estimates and, if applicable, confounder-adjusted estimates and their precision (eg, 95% confidence interval). Make clear which confounders were adjusted for and why they were included

For peer review only

For peer review only - http://bmjopen.bmj.com/site/about/guidelines.xhtml

Appendix V

			(*b*) Report category boundaries when continuous variables were categorized
			(*c*) If relevant, consider translating estimates of relative risk into absolute risk for a meaningful time period
317-327	Other analyses	17	Report other analyses done—eg analyses of subgroups and interactions, and sensitivity analyses
	**Discussion**		
329-335	Key results	18	Summarise key results with reference to study objectives
350-385	Limitations	19	Discuss limitations of the study, taking into account sources of potential bias or imprecision. Discuss both direction and magnitude of any potential bias
388-429	Interpretation	20	Give a cautious overall interpretation of results considering objectives, limitations, multiplicity of analyses, results from similar studies, and other relevant evidence
337-338; 350-353	Generalisability	21	Discuss the generalisability (external validity) of the study results
	**Other information**		
52-58	Funding	22	Give the source of funding and the role of the funders for the present study and, if applicable, for the original study on which the present article is based

*Give information separately for cases and controls in case-control studies and, if applicable, for exposed and unexposed groups in cohort and cross-sectional studies.

Note: An Explanation and Elaboration article discusses each checklist item and gives methodological background and published examples of transparent reporting. The STROBE checklist is best used in conjunction with this article (freely available on the Web sites of PLoS Medicine at http://www.plosmedicine.org/, Annals of Internal Medicine at http://www.annals.org/, and Epidemiology at http://www.epidem.com/). Information on the STROBE Initiative is available at www.strobe-statement.org.

For peer review only

For peer review only - http://bmjopen.bmj.com/site/about/guidelines.xhtml
